# Mechanosensitive Stanniocalcin-1 Suppresses Smooth Muscle Cell Proliferation and Attenuates Experimental Pulmonary Hypertension

**DOI:** 10.1161/CIRCRESAHA.125.327869

**Published:** 2026-09-09

**Authors:** Mariko Kogami, Yuko Kato, Satoko Ito, Keiko Uchida, Hana Inoue, Yuko Hidaka, Shota Tanifuji, Mayumi Yokotsuka, Yoshinari Yamamoto, Toshitaka Nagao, Shinji Abe, Roger R. Reddel, Kazufumi Nakamura, Utako Yokoyama

**Affiliations:** 1 Department of Physiology (M.K., Y.K., S.I., K.U., H.I., Y.H., U.Y.), Tokyo Medical University 13112 , Japan.; 2 Department of Respiratory Medicine (M.K., S.A.), Tokyo Medical University 13112 , Japan.; 3 Department of Anatomy (S.T.), Tokyo Medical University 13112 , Japan.; 4 Department of Anatomic Pathology (M.Y., Y.Y., T.N.), Tokyo Medical University 13112 , Japan.; 5 Department of Advanced Medical Sciences, Faculty of Medicine, Oita University, Japan (Y.K.).; 6 Cancer Research Unit, Children’s Medical Research Institute, Faculty of Medicine and Health, University of Sydney, Australia (R.R.R.); 7 Center for Advanced Heart Failure, Okayama University Hospital, Japan (K.N.).; 8 Division of Cardiology, Shimane University Faculty of Medicine, Izumo, Japan (K.N.).

**Keywords:** cell proliferation, mechanotransduction, cellular, pulmonary arterial hypertension, stanniocalcin-1

## Abstract

**BACKGROUND:**

Idiopathic pulmonary arterial hypertension (IPAH) is driven by progressive vascular remodeling, particularly smooth muscle cell (SMC) proliferation. Current combination vasodilator therapies have markedly improved outcomes; however, prognosis remains poor in subgroups such as patients with respiratory comorbidities. Elevation of intravascular hydrostatic pressure is a hallmark of IPAH, yet its direct role in pulmonary artery SMCs remains unclear. We aimed to identify pressure-responsive mediators using a newly developed hydrostatic pressurization system to model hypertensive hemodynamics.

**METHODS:**

Pulmonary artery SMCs from 4 patients with IPAH were exposed to high hydrostatic pressure (70/40 mm Hg, 60 bpm). Transcriptomic profiling identified differentially expressed genes, which were validated by quantitative polymerase chain reaction. Functional studies included PIEZO1 (piezo type mechanosensitive ion channel component 1) modulation, rhSTC1 (recombinant human stanniocalcin-1) treatment, bromodeoxyuridine incorporation, and Western blotting for cell-cycle regulators. Chronic hypoxia–induced pulmonary hypertension was assessed in wild-type and *Stc1*^–/–^ mice by hemodynamic and histological analyses, with or without intratracheal rhSTC1 administration.

**RESULTS:**

RNA sequencing revealed *STC1* to be a pressure-induced gene in IPAH SMCs. PIEZO1 activation upregulated *STC1*, whereas knockdown blunted this response. *STC1* was upregulated in IPAH lungs, while rhSTC1 reduced pulmonary arterial SMC proliferation and increased p-p53, p21, and p27 expression. *Stc1*^–/–^ mice under hypoxia exhibited significantly higher right ventricular systolic pressure and greater pulmonary arterial medial thickness than wild-type mice. CD68-positive macrophages were increased in *Stc1*^–/–^ mice under normoxia and further elevated with hypoxia. Intratracheal administration of rhSTC1 attenuated PAH in wild-type and *Stc1*^–/–^ mice.

**CONCLUSIONS:**

Elevated hydrostatic pressure drives *STC1* expression via PIEZO1, suggesting an adaptive but insufficient protective response in IPAH. Modulation of STC1 (stanniocalcin-1) may represent a potential therapeutic approach.

**Novelty and Significance**

**What Is Known?**

- Idiopathic pulmonary arterial hypertension is characterized by progressive pulmonary vascular remodeling driven by excessive proliferation of pulmonary arterial smooth muscle cells (PASMCs).
- Elevation of intravascular hydrostatic pressure is a hemodynamic hallmark of idiopathic pulmonary arterial hypertension, but its direct impact on PASMC pathobiology remains unclear.
- Current vasodilator-based therapies improve hemodynamics and symptoms but do not sufficiently prevent the progression of vascular remodeling.

**What New Information Does This Article Contribute?**

- We developed a novel cyclic hydrostatic pressurization culture system to model hypertensive hemodynamic stress and identified mechanoresponsive molecules derived from PASMCs of patients with idiopathic pulmonary arterial hypertension, which provides an understanding of vascular remodeling under hypertensive hemodynamics.
- Intratracheal administration of STC1 (stanniocalcin-1) attenuated remodeling of the pulmonary arteries in a mouse pulmonary arterial hypertension model and may provide a new therapeutic strategy for pulmonary arterial hypertension.

Idiopathic pulmonary arterial hypertension is a fatal disease characterized by abnormal proliferation and inflammation of PASMCs, yet the contribution of mechanical stress to disease progression remains poorly understood. We developed a novel cyclic hydrostatic pressurization system to reproduce hypertensive hemodynamic stress and identify pressure-responsive mediators. Transcriptomic analysis revealed STC1 as a major hydrostatic pressure–induced gene, whose expression is regulated by activation of the mechanosensitive ion channel PIEZO1 (piezo type mechanosensitive ion channel component 1). STC1 activates the p38–p53–p21 pathway, inducing cell-cycle arrest and suppressing PASMC proliferation. In chronic hypoxia–induced PH, *Stc1*^−^^/−^ mice exhibited aggravated vascular remodeling and elevated right ventricular pressure, whereas intratracheal administration of rhSTC1 (recombinant human STC1) significantly suppressed PASMC proliferation and inflammation, leading to marked improvement in pulmonary vascular remodeling. These findings identify STC1 as a mechanosensitive, pressure-responsive protective mediator that counteracts pulmonary vascular remodeling and suggest that STC1 supplementation represents a novel, disease-modifying therapeutic strategy that extends beyond conventional vasodilator approaches.

**Meet the First Author, see p e000771**

**Editorial, see Article by Virk and Bryant**

Pulmonary arterial hypertension (PAH) is a progressive and life-threatening disease characterized by elevated pulmonary arterial pressure, right ventricular hypertrophy, and extensive pulmonary vascular remodeling.^1^ Among the various cell types implicated in PAH pathogenesis, pulmonary arterial smooth muscle cells (PASMCs) play an essential role by undergoing phenotypic modulation and hyperproliferation, ultimately contributing to vascular occlusion and increased pulmonary vascular resistance.^2^ Although vasodilator therapies have advanced, current treatments largely target vasoconstriction and endothelial dysfunction. Efforts to develop therapies that address structural vascular remodeling are ongoing; however, overcoming this aspect of the disease remains a significant challenge.

Mechanical stress has emerged as a critical regulator of vascular cell behavior and remodeling in cardiovascular diseases.^3^ Although the effects of cyclic stretch and shear stress have been extensively studied in the pulmonary circulation,^3^ the contribution of intraluminal hydrostatic pressure—defined as the force exerted perpendicularly on the vessel wall—remains poorly understood. Hydrostatic pressure has been shown to influence endothelial cell morphology, proliferation, calcium signaling, extracellular matrix composition, adhesion molecule expression, and barrier permeability,^4^ all of which are implicated in endothelial dysfunction—a common feature of hypertension, atherosclerosis, stroke, and pathological angiogenesis. However, despite its direct impact on the vascular wall, the effect of hydrostatic pressure on vascular smooth muscle cells—the most abundant structural component of the vascular wall—has received little attention. Notably, vascular smooth muscle cells are highly responsive to their local microenvironment, integrating mechanical cues, soluble factors, cell-matrix interactions, and inflammatory mediators.^5^ They exhibit remarkable phenotypic plasticity, shifting from a quiescent, contractile state to a synthetic, proliferative phenotype under pathological conditions.^6^ Based on these characteristics, we hypothesized that vascular smooth muscle cells may act as central mediators of hydrostatic pressure–induced remodeling in PAH.

We previously developed a custom-built culture system capable of applying cyclic hydrostatic pressure to cells.^7,8^ For the present study, we modified the system to reproduce both the magnitude and frequency of cyclic hydrostatic pressure characteristic of PAH, thereby enabling more physiologically relevant modeling of the disease condition. Using this refined system, we exposed PASMCs derived from patients with idiopathic pulmonary arterial hypertension (IPAH) to pressure cycles that mimic hypertensive hemodynamic stress. Subsequently, transcriptomic profiling was performed to identify pressure-sensitive genes selectively upregulated in IPAH PASMCs. Functional analyses were conducted to determine the roles of these genes in driving vascular remodeling. Our findings uncovered a previously unrecognized pressure-responsive mechanotransduction pathway in human PASMCs.

## Methods

### Data Availability

RNA sequencing data have been deposited in the Gene Expression Omnibus under accession number GSE317316 and will be made publicly available upon publication. All other data supporting the findings of this study are available from the corresponding author upon reasonable request. The full experimental details are presented in the Supplemental Material.

### Study Design

This preclinical study used patient-derived PASMCs and mouse models of PH. In vitro, gene expression changes under mechanical stimulation were assessed using a custom-built hydrostatic pressure apparatus. Pressure-responsive molecules identified in vitro were further evaluated in vivo using PASMCs from patients with IPAH, a hypoxia-induced PH mouse model, and genetically modified mice.

### Human Samples

PASMCs and lung tissues from patients with IPAH (n=11) were obtained at Okayama University with institutional review board approval and written informed consent (reference number: 1511-017). Non-PAH controls (n=13) without lung cancer were obtained at Tokyo Medical University with institutional review board approval. Subject demographics, including age and sex, are summarized in Major Resources Table. In addition, commercially available non-PAH control cells were used (CC-2581; Lonza). Primary human PASMCs at passages 3 to 10 were used for all experiments. Additional details, including cell source and passage numbers, are provided in the Major Resources Table. Details of the samples used for each figure are summarized in Table S1.

### Hydrostatic Pressure Stimulation

The hydrostatic pressure apparatus consisted of a pressure chamber, regulator, and compressor (Figure S1A through S1C). Cell culture dishes were placed inside the pressure chamber, and air drawn from a CO_2_ incubator was periodically introduced into the chamber via the regulator to apply cyclic hydrostatic pressure to the cells. PASMCs were seeded at 1.5×10^5^ cells per 35-mm dish and placed in the pressure chamber. Cyclic hydrostatic pressure of 70/40 mm Hg (mean 50 mm Hg) at 60 bpm was applied for 72 hours. Pressure waveforms were verified using a pressure transducer (Figure S1B). Atmospheric pressure control cells were cultured under atmospheric pressure in the same CO_2_ incubator.

### Measurement of Dissolved Oxygen in Culture Medium

Dissolved oxygen in cell-free culture medium was measured using a platinum electrode (Po_2_-150S; Bio Research Center Co., Ltd., Japan). After insertion of the electrode into the medium, it was carefully positioned to avoid contact with the dish surface and allowed to equilibrate for 30 minutes at 37 °C under 5% CO_2_ to ensure stabilization of oxygen levels. The measured oxygen values were corrected to a reference partial pressure of 140 mm Hg, corresponding to air-equilibrated conditions at 37 °C under 5% CO_2_. Dissolved oxygen was measured under atmospheric pressure or cyclic hydrostatic pressure (70/40 mm Hg, 60 bpm). Dissolved oxygen levels were summarized in Table S2.

### RNA Sequencing

Total RNA was extracted from cultured PASMCs using the RNeasy Mini Kit (Qiagen, Hilden, Germany, Cat. No. 74104) according to the manufacturer’s instructions, and RNA integrity was confirmed (RNA integrity number >7.0). Library preparation, sequencing, and primary processing were performed by Rhelixa (Tokyo, Japan). Poly(A)-selected libraries were sequenced on an Illumina NovaSeq X Plus platform to generate paired-end 150-bp reads at a depth of ≈26.7 million reads/sample. Reads were quality-checked, adapter-trimmed, and aligned to the GRCh38 reference genome. Differential expression analysis was performed using DESeq2, and mean-average plots were generated using the plotMA function to visualize log2-fold changes versus mean normalized counts. As no genes met the predefined statistical significance threshold in the paired differential expression analysis, annotated genes with a baseMean ≥50 were used for subsequent exploratory analyses to reduce the influence of unstable fold changes derived from low-abundance transcripts. Gene set enrichment analysis was performed using these genes, ranked by log2-fold change, and implemented using fgsea. Transcription factor enrichment analysis was performed with iRegulon using analyzed upregulated (log2-fold change >0.25, Table S3) and downregulated (log2-fold change <−0.25, Table S4) gene sets.

### Mouse Models

Wild-type (WT) and stanniocalcin-1 (*Stc1*)^−/−^ mice on a C57BL/6J background (male and female, 3 months old)^9^ were maintained under normoxic (21% O_2_) or hypoxic (10% O_2_) conditions for 4 weeks. For therapeutic studies, rhSTC1 (recombinant human stanniocalcin-1; R&D Systems, Minneapolis, MN, Cat. No. 9400-SO) was administered intratracheally (2 µg/50 µL PBS, every 72 hours for 4 weeks) based on previous reports^10^ and pharmacokinetic pilot data (Figure S2).

No formal a priori sample size calculation was performed. Sample sizes were initially informed by previous studies using comparable hypoxia-induced PH mouse models which typically included 6 to 12 animals per group.^11–13^ To quantitatively evaluate the adequacy of the study design, a post hoc design-sensitivity analysis was performed for right ventricular systolic pressure. Based on previous studies,^11–13^ a biologically meaningful between-group difference of 10 mm Hg and a conservative SD of 5 mm Hg in all groups were assumed. For the PAH model, 10 animals per group provided 98.8% power at an α level of 0.05. For the treatment model, 6 animals per group provided 87.6% power at a 2-sided α level of 0.05. Because 5 animals per group provided <80% power, the minimum required sample size under these assumptions was 6 animals per group.

### Hemodynamics and Morphometry

Echocardiography and left/right heart catheterization were performed to assess hemodynamics. Fulton index (right ventricular/[left ventricular + septum]) was calculated from heart weights. Paraffin-embedded lung sections were stained with Elastica-Masson-Goldner to quantify medial wall thickness: ([external elastic lamina diameter – internal elastic lamina diameter]/external elastic lamina diameter)×100%. Medial wall thickness was calculated for each pulmonary artery, and the average of 15 vessels with an external diameter of 15 to 100 µm accompanying noncartilaginous bronchioles and sharing a common adventitial sheath was calculated per sample.

### Molecular Assays

Quantitative polymerase chain reaction (SYBR), Western blot, ELISA, 5-bromo-2′-deoxyuridine (BrdU) incorporation assay, immunohistochemistry, immunofluorescence, and terminal deoxynucleotidyl transferase dUTP nick end labeling staining were performed as described previously.^14–16^ Primer/probe sequences and antibody details are provided in the Major Resources Table and Supplemental Material.

### Statistics

Analyses were performed using GraphPad Prism (v9.2.0). Two-group comparisons were performed using the Mann-Whitney *U* test, whereas multiple-group comparisons were assessed using the Kruskal-Wallis test followed by post hoc Mann-Whitney *U* tests. For experiments involving repeated measurements from the same cell lot, 2-group comparisons were performed using a linear mixed-effects model, whereas multiple-group comparisons were performed using a linear mixed-effects model followed by pairwise comparisons with Tukey adjustment using the emmeans package in R (v4.2.2). Data are expressed as mean ± SEM. *P* values are indicated in the figures.

### Study Approval

Human studies were approved by the Ethical Guidelines Committee of Tokyo Medical University and Okayama University (T2023-0084, T2026-0008, and 1511-017, respectively) and adhered to the principles outlined in the Declaration of Helsinki (10th version, Fortaleza, 2013). Animal studies were approved by the Tokyo Medical University Institutional Animal Care and Use Committee (R7-058) and were conducted in accordance with the Animal Research: Reporting of In Vivo Experiments guidelines.

## Results

### Cyclic Hydrostatic Pressurization Induces Expression of Genes Associated With Proliferation and Dedifferentiation in IPAH-Derived PASMCs

To examine how hydrostatic pressure affects gene expression, PASMCs derived from patients with IPAH were subjected to cyclic hydrostatic pressurization (70/40 mm Hg, 60 bpm) for 72 hours, followed by RNA sequencing (Figure 1A).

Gene set enrichment analysis using Gene Ontology Biological Process databases revealed significant enrichment of proliferation-related pathways such as G2M checkpoint and E2F targets under cyclic hydrostatic pressure (normalized enrichment score >2.0; Figure 1B). To gain a comprehensive understanding of the biological processes involved, we conducted Gene Ontology Biological Process enrichment analysis. Consistently, the Gene Ontology Biological Process enrichment network map revealed that clusters of cell cycle–related gene sets were predominantly upregulated, which are highlighted in red for visualization (Figure 1C).

**Figure 1.**
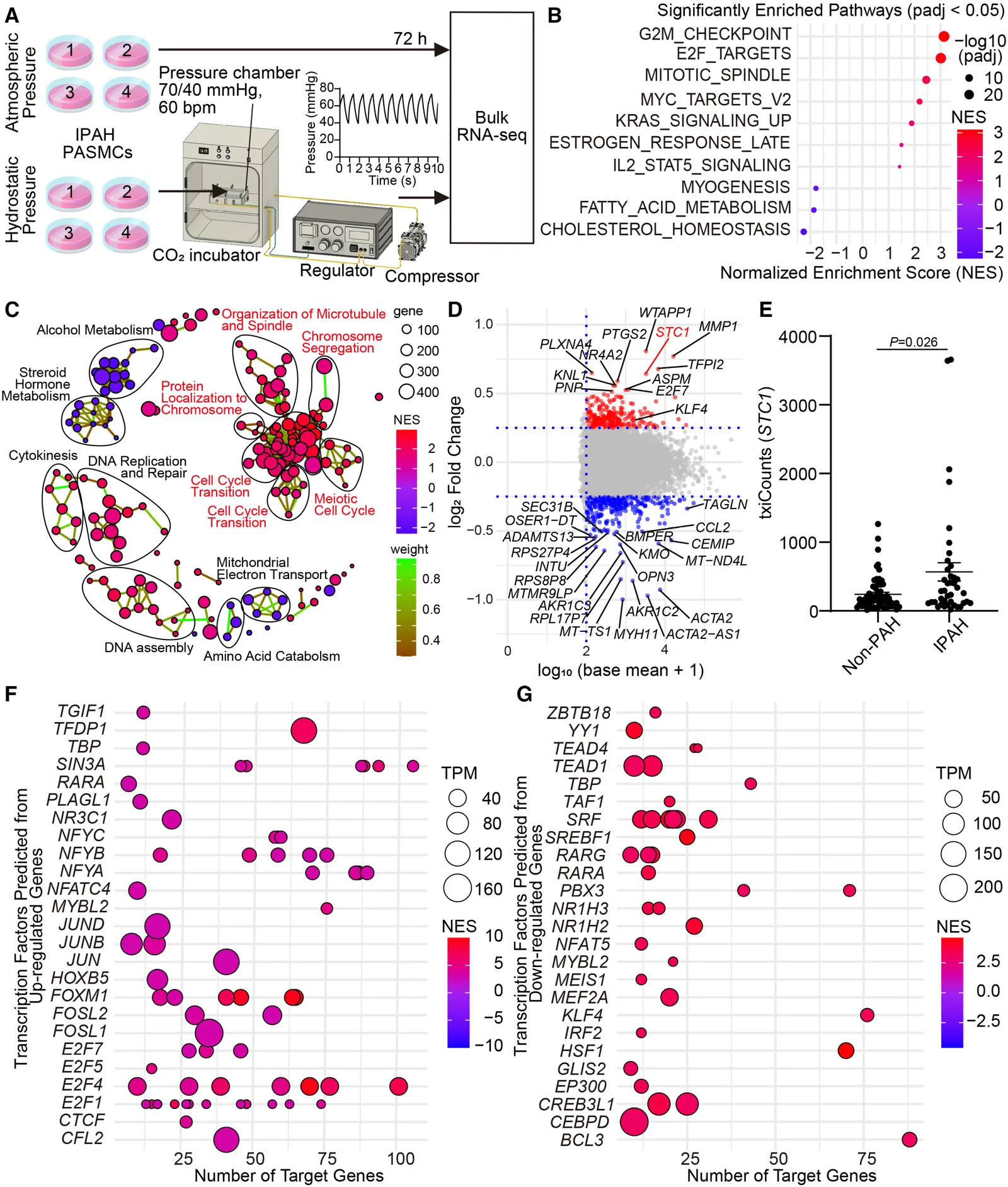
**Transcriptomic changes in pulmonary artery smooth muscle cells (PASMCs) subjected to cyclic hydrostatic pressure. A**, Experimental protocol for cyclic hydrostatic pressure loading. PASMCs derived from patients with idiopathic pulmonary arterial hypertension (IPAH; n=4) were cultured to ≈80% confluence and then subjected to cyclic hydrostatic pressure (70/40 mm Hg, 60 bpm) for 72 h using a custom-designed system. Cells were subsequently harvested for RNA sequencing (RNA-seq). **B**, Dot plots show the top 10 enriched gene sets with adjusted *P* value (*P*_adj_)<0.05 for the Kyoto Encyclopedia of Genes and Genomes. Circle size represents the number of genes in each gene set, and normalized enrichment score (NES) indicates the degree of enrichment. **C**, Enrichment map generated from Gene Ontology Biological Process gene set enrichment analysis comparing hydrostatic pressure and atmospheric pressure. Clusters with ≥3 nodes were annotated. Edge color represents the similarity weight between connected gene sets based on pairwise term similarity. **D**, MA (log ratio [M] versus mean average [A]) plot of RNA-seq data, showing log_2_-fold change vs log_10_(base mean + 1) expression levels. Genes with log_2_-fold change >0.5 (red) or <–0.5 (blue) and log_10_(base mean + 1)≥2 are highlighted. **E**, STC1 (stanniocalcin-1) expression in human lung RNA-seq data sets. Transcript-level counts (txiCounts) of STC1 were extracted from a previously published RNA-seq data set comparing non-PAH lungs (n=40) and IPAH lungs (n=52). Data are presented as individual samples. Data are presented as mean ± SEM. Statistical significance was determined using the Mann-Whitney *U* test. *P* values are indicated in the figure. **F** and **G**, Dot plots show candidate upstream transcription factors identified by iRegulon analyses. **F**, Candidate genes potentially regulating the upregulated gene set; **G**, candidate genes potentially regulating the downregulated gene set. Some genes have multiple results because iRegulon identifies multiple significantly enriched DNA-binding motifs corresponding to the same transcription factors and each motif is displayed separately. TPM indicates transcripts per million.

Mean-average plot analysis identified 250 upregulated and 291 downregulated genes with log_2_-fold change >0.25 or <–0.25 and log_10_(base mean + 1) >2.0 (Figure 1D; Tables S3 and S4). Among the upregulated genes, *STC1* was one of the most strongly induced transcripts (Figure 1D). Consistently, analysis of a previously published lung RNA sequencing dataset^17^ demonstrated that *STC1* expression was significantly increased in IPAH lungs compared with that in failed donor controls (Figure 1E; Figure S3). Expression of the key contractile phenotype markers *MYH11* (myosin heavy chain 11), *ACTA2* (smooth muscle α-actin), and *TAGLN* (transgelin) was consistently downregulated. Concomitantly, *KLF4* (Krüppel-like factor 4), a transcription factor recognized as a canonical marker of dedifferentiation,^18^ was upregulated. Transcription factor analysis using iRegulon identified transcription factors potentially regulating the upregulated (Figure 1F) and downregulated (Figure 1G) genes. Consistent with the enrichment of cell cycle–related pathways, iRegulon analysis identified several genes known to positively regulate cell cycle progression, including *TFDP1*, *FOSL1*, and *E2Fs*, as candidate upstream regulators of the upregulated gene set (Figure 1F).^19–21^ iRegulon analysis identified KLF4 as a candidate upstream regulator of the downregulated gene set (Figure 1G). KLF4 has been reported to repress smooth muscle cell marker genes, including *ACTA2*, *TAGLN*, and *MYH11*, by interfering with serum response factor-dependent transcription.^22^ Consistent with this, *KLF4* was upregulated, whereas *ACTA2*, *TAGLN*, and *MYH11* were downregulated in our transcriptomic analysis (Figure 1D). These data suggest that elevating intravascular pressure directly promotes a phenotypic shift toward dedifferentiation in PASMCs. To explore a regulator of pressure-induced cell proliferation, we focused on the glycoprotein STC1 (stanniocalcin-1), which was strongly induced and has been implicated in paracrine/autocrine regulation of cell proliferation.^6,23,24^

### STC1 Expression Is Elevated in Patients With IPAH and Hypoxia-Induced PH Model Mice

Immunohistochemistry of human lung tissues demonstrated markedly increased expression of STC1 in pulmonary arteries of IPAH patients compared with non-PAH controls (Figure 2A; Figure S4), which was confirmed by semiquantitative analysis showing a significant increase (Figure 2B). Consistently, Western blot analysis of culture supernatants showed significantly higher levels of secreted STC1 in PASMCs from patients with IPAH than in those from non-PAH controls (Figure 2C and 2D). Basal mRNA expression analysis also demonstrated elevated *STC1* levels in IPAH-derived PASMCs compared with non-PAH PASMCs under unstimulated conditions (Figure 2E). Exposure to cyclic hydrostatic pressure for 72 hours did not change *STC1* expression levels in the non-PAH PASMCs; however, PASMCs from patients with IPAH exhibited a significant pressure-induced upregulation of *STC1* mRNA (Figure 2F).

**Figure 2.**
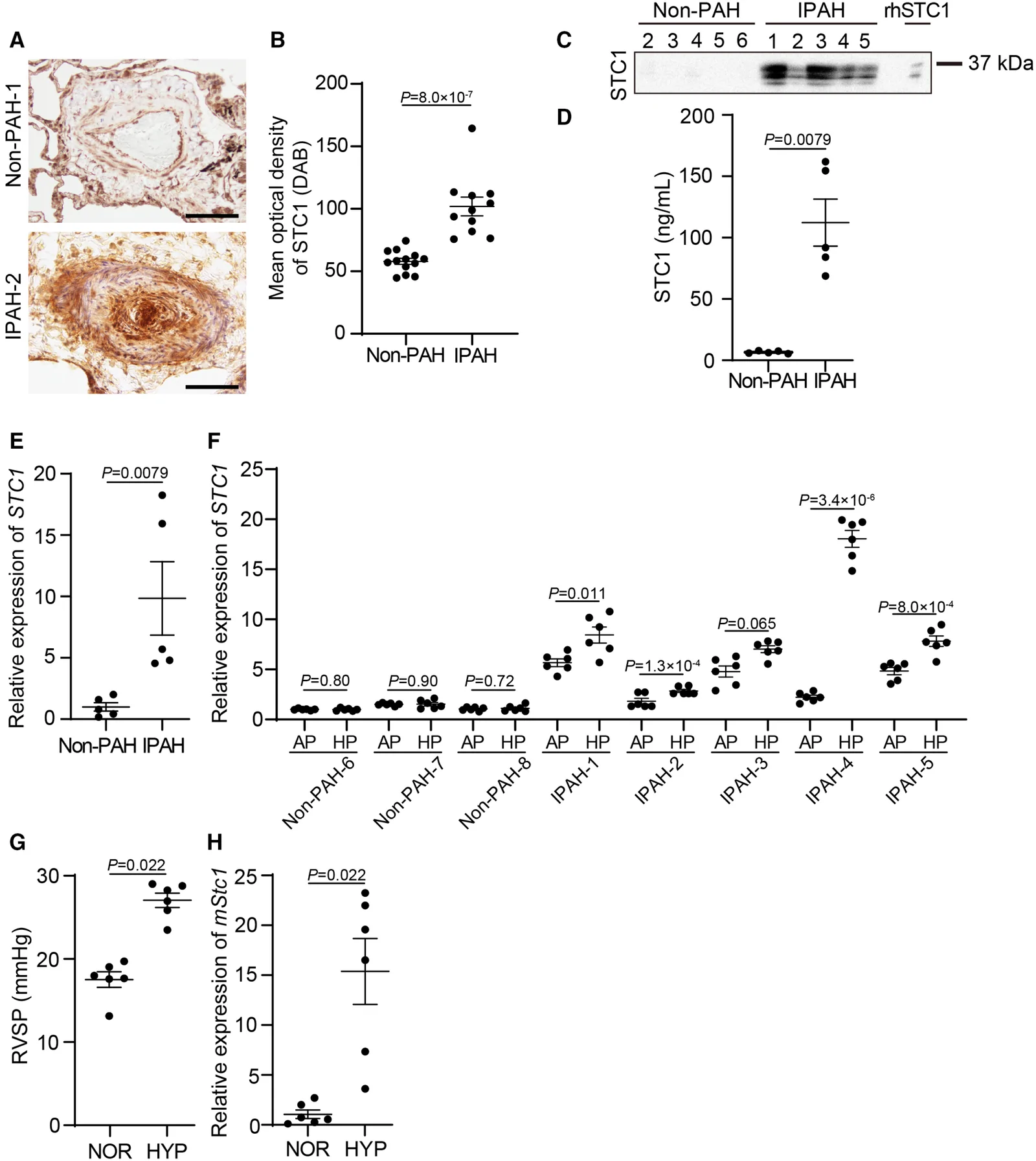
**Expression of STC1 (stanniocalcin-1) in pulmonary artery smooth muscle cells (PASMCs) from patients with idiopathic pulmonary arterial hypertension (IPAH) and lungs from hypoxia-induced pulmonary arterial hypertension (PAH) model mice. A**, Representative immunohistochemistry images of STC1 expression in pulmonary arteries from non-PAH donors and patients with IPAH. Scale bars, 100 µm. **B**, Semiquantitative analysis of STC1 expression in pulmonary arteries from non-PAH (n=13) and IPAH patients (n=11). Quantified as mean optical density (DAB). **C** and **D**, Western blot analysis of STC1 in the culture supernatant of PASMCs from non-PAH controls (n=5) and patients with IPAH (n=5). PASMCs were grown in serum-free DMEM for 24 h. The supernatant was collected and concentrated 22-fold using a column concentrator. Representative blot (**C**) and quantification of STC1 concentrations based on band density measurements using reference bands generated from rhSTC1 (recombinant human STC1) at 500 µg/mL (**D**). **E**, Basal expression of *STC1* mRNA in PASMCs under unstimulated conditions. PASMCs derived from non-PAH controls (n=5) and IPAH patients (n=5) were analyzed by quantitative polymerase chain reaction. **F**, Relative expression of *STC1* mRNA in PASMCs derived from patients with non-PAH and patients with IPAH after exposure to cyclic hydrostatic pressure for 72 h. Right ventricular systolic pressure (RVSP, **G**) and relative expression of *Stc1* mRNA in lung (**H**) in hypoxia-induced PAH model mice (C57BL/6J; fraction of inspired oxygen 10% for 4 weeks; n=6; NOR, normoxia; HYP, hypoxia). Data are presented as mean ± SEM. Statistical significance was determined using the Mann-Whitney *U* test for **B**, **D**, **E**, **G**, and **H**, and a linear mixed-effects model for **F**. For **F**, post hoc pairwise comparisons were performed using emmeans with Tukey adjustment. *P* values are indicated in the figure. AP indicates atmospheric pressure; and HP, hydrostatic pressure.

Similarly, in a mouse model of hypoxia-induced pulmonary hypertension (PH), characterized by significantly elevated right ventricular systolic pressure (Figure 2G), pulmonary *Stc1* mRNA levels were markedly increased (Figure 2H).

### Mechanotransduction via PIEZO1 and HIF-1α Signaling Regulates STC1 Expression

To investigate the mechanism by which hydrostatic pressure induces *STC1* expression, IPAH-derived PASMCs were stimulated with agonists for mechanosensitive channels: TRPV4 (transient receptor potential vanilloid 4; GSK1016790A), TRPM7 (transient receptor potential melastatin 7; naltriben), and PIEZO1 (piezo type mechanosensitive ion channel component 1; Yoda1). Among these, PIEZO1 activation significantly increased *STC1* expression in PASMCs from 3 independent non-PAH patients and 3 independent IPAH patients (Figure 3A; Figure S5A), with a greater magnitude of induction observed in IPAH cells.

**Figure 3.**
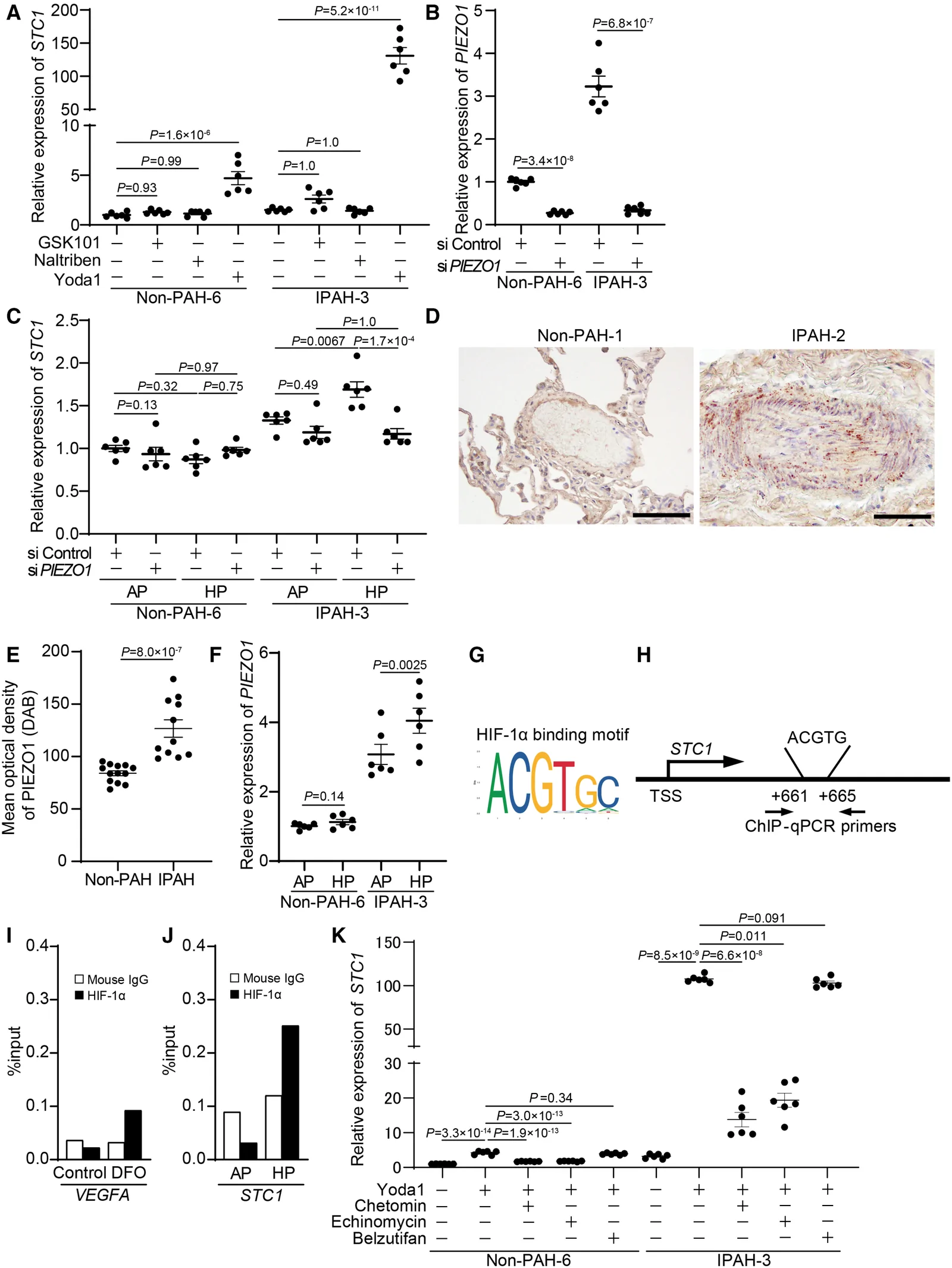
**Regulation of STC1 (stanniocalcin-1) expression by mechanotransduction. A**, Effects of induction of *STC1* expression by mechanoreceptor activators. Relative expression of *STC1* mRNA following stimulation of mechanosensitive receptors in pulmonary artery smooth muscle cells (PASMCs) from nonpulmonary arterial hypertension (non-PAH-6) and patients with idiopathic pulmonary arterial hypertension (IPAH-3). Cells were stimulated with GSK1016790A (GSK101, 10 nmol/L), an agonist of TRPV4 (transient receptor potential vanilloid 4); naltriben (20 μmol/L), an agonist of TRPM7 (transient receptor potential melastatin 7); or Yoda1 (20 μmol/L), an agonist of PIEZO1 (piezo type mechanosensitive ion channel component 1). *STC1* mRNA expression was quantified using quantitative polymerase chain reaction (qPCR). **B**, Efficiency of *PIEZO1* knockdown. PASMCs were transfected with small interfering RNA (siRNA) targeting *PIEZO1* (si *PIEZO1*) or control siRNA (si Control), and knockdown efficiency was assessed by qPCR. Relative expression of *PIEZO1* mRNA in non-PAH-6 and IPAH-3. **C**, Effect of *PIEZO1* knockdown on *STC1* expression under cyclic hydrostatic pressure. Relative expression of *STC1* mRNA in PASMCs from non-PAH and IPAH patients (non-PAH-6, IPAH-3) following exposure to cyclic hydrostatic pressure for 72 h with or without *PIEZO1* knockdown. **D**, Representative immunohistochemistry image of PIEZO1 expression in pulmonary arteries from non-PAH donors and patients with IPAH. Scale bars, 100 µm. **E**, Semiquantitative analysis of PIEZO1 expression in pulmonary arteries from non-PAH (n=13) and IPAH patients (n=11), quantified as mean optical density (DAB). **F**, Changes in *PIEZO1* expression in PASMCs following exposure to cyclic hydrostatic pressure. PASMCs from non-PAH (Non-PAH-6) and IPAH patients (IPAH-3) were exposed to cyclic hydrostatic pressure for 72 h. Relative expression of *PIEZO1* was assessed by qPCR. **G**, HIF-1α (hypoxia-inducible factor-1 alpha) binding motif identified from the JASPAR database. **H**, Schematic representation of chromatin immunoprecipitation (ChIP)-qPCR primer design for the *STC1* promoter region, including the predicted HIF-1α binding site. **I**, ChIP-qPCR analysis of HIF-1α binding to the *VEGFA* promoter region as a positive control. PASMCs (IPAH-1) were treated with deferoxamine (DFO) for 6 h to induce HIF-1α stabilization. **J**, ChIP-qPCR analysis of HIF-1α binding to the *STC1* promoter region. PASMCs (IPAH-1) were exposed to cyclic hydrostatic pressure for 3 h. Mouse IgG was used as a negative control. **K**, Effect of HIF (hypoxia-inducible factor) inhibition on PIEZO1-mediated *STC1* upregulation. Relative expression of *STC1* mRNA in PASMCs from IPAH patients (non-PAH-6, IPAH-3) after treatment with HIF inhibitors during PIEZO1-mediated upregulation of STC1. HIF-1α was inhibited using chetomin (100 nmol/L) or echinomycin (30 nmol/L), and HIF-2α was inhibited using belzutifan (10 nmol/L). PIEZO1 was activated by Yoda1. Expression levels were quantified by qPCR. Data are presented as mean ± SEM (n=6). Statistical significance for panels **A–C**, **F**, and **K** was determined using linear mixed-effects models. For multiple-group comparisons, post hoc pairwise comparisons were performed using emmeans with Tukey adjustment. Statistical significance for **E** was determined using the Mann-Whitney *U* test. *P* values are indicated in the figure. AP indicates atmospheric pressure; and HP, hydrostatic pressure.

Small interfering RNA-targeted PIEZO1 significantly decreased *PIEZO1* mRNA in non-PAH and IPAH PASMCs (Figure 3B; Figure S5B). PASMCs from non-PAH donors (n=3 independent lines) did not exhibit pressure-induced STC1 upregulation (Figure 3C; Figure S5C). In contrast, small interfering RNA-mediated knockdown of *PIEZO1* effectively suppressed the hydrostatic pressure–induced upregulation of *STC1* in PASMCs from patients with IPAH (n=3 independent lines; Figure 3C; Figure S5C). Immunohistochemistry of human lung tissues demonstrated that *PIEZO1* expression was increased in patients with IPAH compared with non-PAH controls (Figure 3D; Figure S6A and S6B), which was confirmed by semiquantitative analysis showing a significant increase (Figure 3E). These findings are consistent with a previous report.^25^ Cyclic hydrostatic pressure did not induce *PIEZO1* expression in PASMCs from non-PAH donors, whereas a significant induction was observed in PASMCs from patients with IPAH (Figure 3F; Figure S6C).

Previous studies demonstrated that PIEZO1 activation increases HIF-1α (hypoxia-inducible factor-1α) expression.^26–28^ Notably, a putative HIF-1α binding site is present in the promoter region of STC1 (Figure 3G and 3H). To determine whether hydrostatic pressure induces STC1 via HIF-1α, we first assessed HIF-1α nuclear translocation using immunocytochemistry. Cyclic hydrostatic pressure for 3 hours significantly increased nuclear localization of HIF-1α in PASMCs from patients with IPAH; this effect was attenuated by silencing of *PIEZO1*, whereas no increase was observed in non-PAH cells (Figure S7A through S7G).

To further examine whether HIF-1α binds to the *STC1* promoter, we performed chromatin immunoprecipitation followed by quantitative polymerase chain reaction. As a positive control for HIF-1α binding, cells were treated with deferoxamine to mimic hypoxia, which increased HIF-1α binding to the *VEGFA* promoter region (Figure 3I). Under cyclic hydrostatic pressure for 3 hours, corresponding to the condition that induced HIF-1α nuclear translocation, HIF-1α binding to the STC1 promoter region was increased in PASMCs from patients with IPAH (Figure 3J).

To investigate whether PIEZO1-mediated induction of *STC1* depends on HIF signaling, cells were treated with the HIF-1α inhibitors chetomin and echinomycin combined with PIEZO1 stimulation. These treatments canceled PIEZO1-induced *STC1* expression (Figure 3K; Figure S7H). To further assess the specificity of HIF-1α involvement, we examined the HIF-2α (hypoxia-inducible factor-2α) inhibitor belzutifan, as HIF-2α activation is known to worsen PH in vivo.^29^ PIEZO1-induced *STC1* expression was unaffected by HIF-2α inhibition (Figure 3K; Figure S7H), suggesting that hydrostatic pressure induces *STC1* expression via the PIEZO1-HIF-1α signaling axis.

### STC1 Inhibits PASMC Proliferation

To assess the effect of STC1 on cell proliferation in the pulmonary arteries, immunohistochemistry for Ki-67 was performed. Ki-67-positive cells were increased in the pulmonary arteries of IPAH lungs compared with those of non-PAH controls (Figure 4A and 4B; Figure S8).

**Figure 4.**
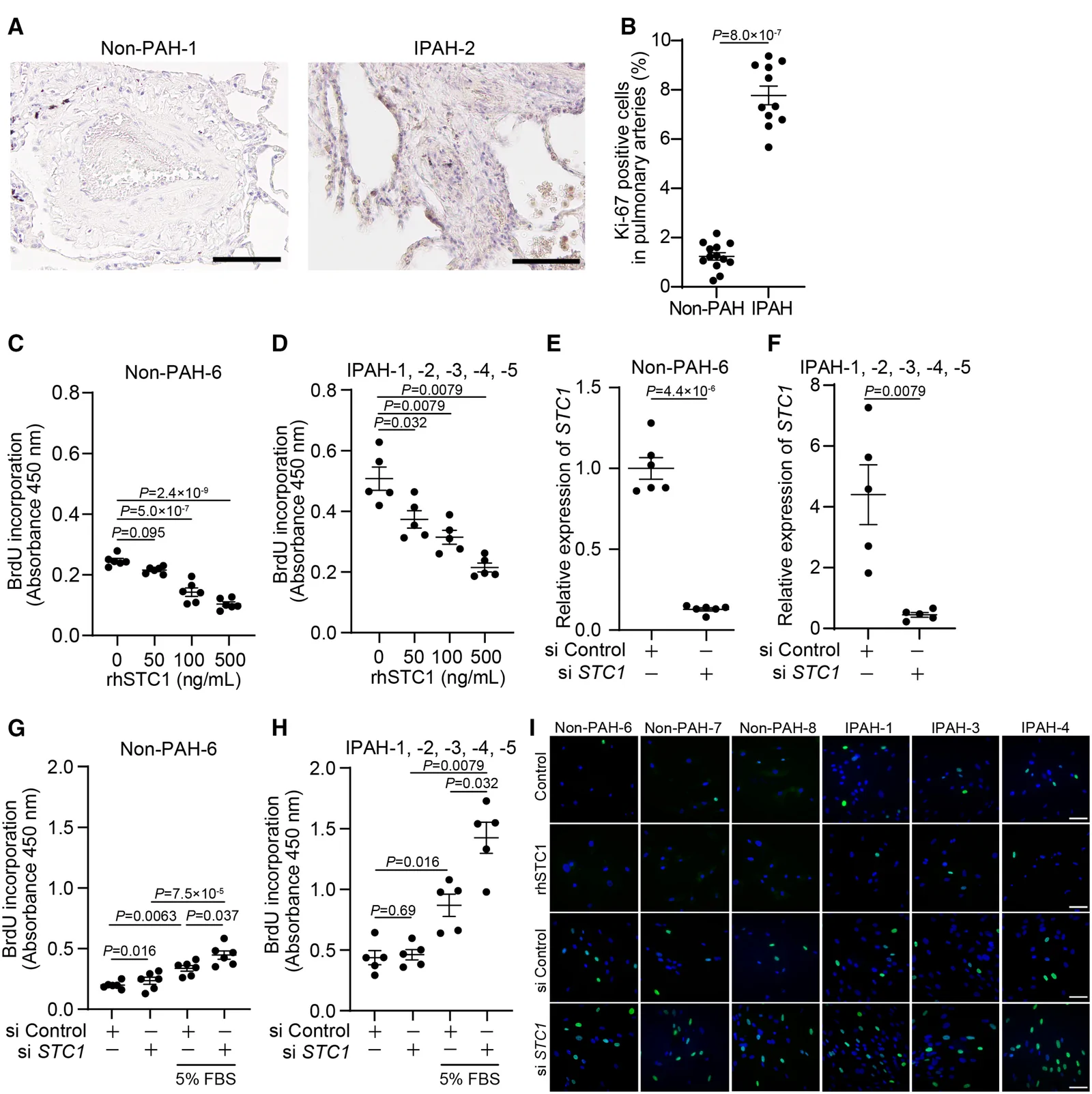
**STC1 (stanniocalcin-1) regulates cell proliferation in pulmonary artery smooth muscle cells (PASMCs). A**, Representative immunohistochemistry image of Ki-67 expression in the pulmonary arteries from nonpulmonary arterial hypertension (non-PAH) donors and patients with idiopathic pulmonary arterial hypertension (IPAH). Scale bars, 100 µm. **B**, Quantification of Ki-67-positive PASMCs in the pulmonary arteries from non-PAH (n=13) and IPAH patients (n=11). The percentage of Ki-67-positive PASMCs was calculated as the mean value from 5 randomly selected fields per section. **C** and **D**, Effect of rhSTC1 (recombinant human STC1) on cell proliferation. Non-PAH-6 PASMCs (**C**) and IPAH-derived PASMCs (**D**). Proliferation was assessed by 5-bromo-2′-deoxyuridine (BrdU) incorporation assay and measured by ELISA. Donor-derived PASMCs were tested in a single lot with 6 replicates (n=6), and IPAH-derived PASMCs represent the mean value from each of 5 independent patient-derived PASMC lines (n=5). Each dot represents an individual replicate (non-PAH-6) or a patient-derived PASMC line (IPAH). **E** and **F**, PASMCs were transfected with small interfering RNA (siRNA) targeting *STC1* (si *STC1*) or control siRNA (si Control), and knockdown efficiency was assessed by quantitative polymerase chain reaction (qPCR). Relative expression of *STC1* mRNA in PASMCs from non-PAH-6 (**E**) and IPAH patients (n=5 independent patient-derived PASMC lines; **F**) is shown. Each dot represents the mean value of a single donor- or patient-derived PASMC line. STC1 expression was quantified by qPCR. **G** and **H**, Proliferation following siRNA-mediated knockdown of *STC1*, measured by BrdU incorporation assay with ELISA readout in donor- and IPAH-derived PASMCs. Each dot represents data from an individual replicate of non-PAH-6 (**G**) or IPAH-derived PASMC lines (**H**). **I**, Representative 5-ethynyl-2′-deoxyuridine (EdU) incorporation images under the indicated conditions. PASMCs from non-PAH (n=3 independent donor-derived lines) and IPAH patients (n=3 independent patient-derived lines) were analyzed. Cells were treated under control conditions, with recombinant human STC1 (rhSTC1; 100 ng/mL), or transfected with control siRNA (si Control) or *STC1*-targeting siRNA (si *STC1*). EdU (green) labels proliferating cells, and nuclei were counterstained with Hoechst (blue). Scale bars, 50 µm. Data are expressed as mean ± SEM. Panels **B**, **D**, **F**, and **H** were analyzed using the Mann-Whitney *U* test (2-group comparisons) or the Kruskal-Wallis test followed by post hoc Mann-Whitney *U* tests (multiple-group comparisons), as appropriate. **C**, **E**, and **G** were analyzed using linear mixed-effects models, with post hoc pairwise comparisons performed using emmeans with Tukey adjustment for multiple-group comparisons. *P* values are indicated in the figure.

To evaluate the functional role of STC1, rhSTC1 was administered to PASMCs derived from patients with non-PAH and IPAH. BrdU incorporation assays revealed dose-dependent suppression of proliferation in both cell types following rhSTC1 treatment (Figure 4C and 4D; Figure S9A and S9B), suggesting that STC1 negatively regulates PASMC proliferation. Furthermore, small interfering RNA-mediated knockdown of *STC1* markedly increased BrdU incorporation (Figure 4E through 4H; Figure S9C through S9E), indicating enhanced proliferative activity in the absence of STC1. In addition, representative EdU incorporation images were obtained, and similar trends in proliferation were observed across 3 independent non-PAH and 3 independent IPAH PASMC lines (Figure 4I). Taken together, these results demonstrate that STC1 expression is upregulated in IPAH and acts as an endogenous negative regulator of PASMC proliferation.

### STC1 Increases Expression of Cell Cycle Arrest–Related Proteins

To elucidate the underlying molecular mechanisms of STC1-mediated inhibition of cell proliferation, rhSTC1 was administered to IPAH-derived PASMCs, and the expression of cell cycle arrest–related proteins was assessed by Western blot analysis. First, we examined the time course of p38 phosphorylation, which is known to respond relatively early among cell cycle regulatory molecules.^30–32^ rhSTC1 stimulation promoted phosphorylation of p38, with the maximal response observed at 2 hours after stimulation (Figure 5A), showing a significant increase (Figure 5B and 5C). Next, we analyzed the p53-p21 pathway, a reported downstream signal of p38,^33^ in samples collected 12 hours after rhSTC1 administration. rhSTC1 stimulation increased the expression of phospho-p38, phospho-p53, p21, and p27, while reducing CDK2 (cyclin-dependent kinase 2) expression, indicating activation of cell cycle inhibitory pathways (Figure 5B and 5C).^34,35^ Furthermore, pharmacological inhibition of p38 (SB203580), p53 (pifithrin-α), or p21 (UC2288) attenuated the rhSTC1-mediated reduction in BrdU incorporation, indicating that the p38-p53-p21 axis contributes to the antiproliferative effects of STC1 (Figure 5D through 5F).

**Figure 5.**
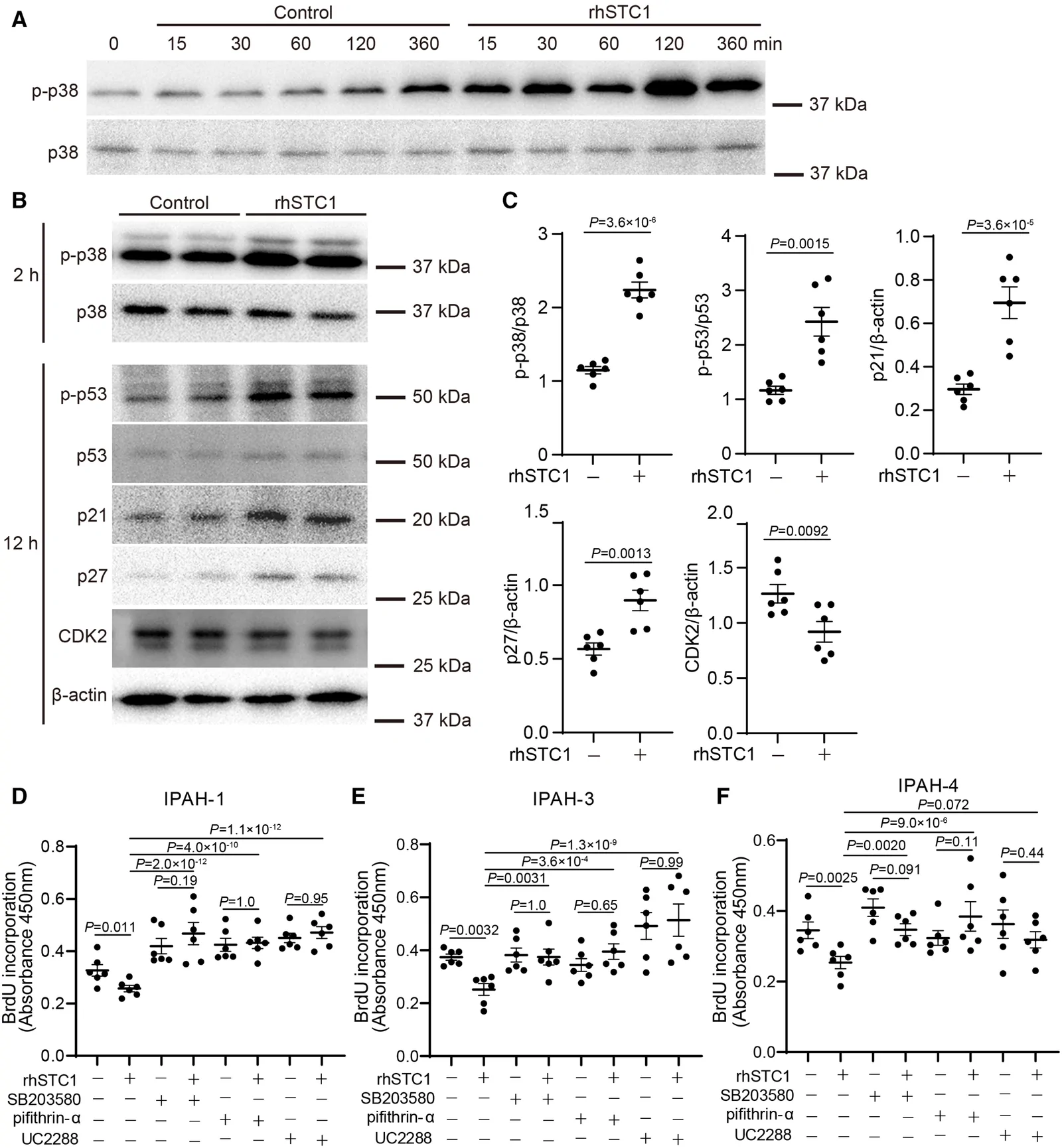
**Effect of STC1 (stanniocalcin-1) on cell cycle regulatory proteins in pulmonary artery smooth muscle cells (PASMCs).** PASMCs derived from a patient with idiopathic pulmonary arterial hypertension (IPAH-4) were treated with rhSTC1 (recombinant human stanniocalcin-1; 100 ng/mL) and collected at predetermined time points for Western blot analysis. **A**, Time course of phospho-p38 (p-p38) and total p38 expression at 0, 15, 30, 60, 120, and 360 min after rhSTC1 treatment. **B**, Representative Western blot images showing expression of p-p38, total p38, phospho-p53 (p-p53), total p53, p21, p27, CDK2, and β-actin at the indicated time points (p38 and p-p38: 2 h; other proteins: 12 h). **C**, Quantitative analysis of Western blot data. Levels of p-p38 were normalized to that of total p38, p-p53 to total p53, and other proteins to β-actin. **D** and **F**, Effects of pathway inhibition on STC1-mediated suppression of cell proliferation. PASMCs from patients with IPAH (**D**; IPAH-1, **E**; IPAH-3, and **F**; IPAH-4) were analyzed. Cell proliferation was assessed by 5-bromo-2′-deoxyuridine (BrdU) incorporation assay with ELISA. Cells were treated with rhSTC1 in the presence or absence of the p38 inhibitor SB203580, the p53 inhibitor pifithrin-α, or the p21 inhibitor UC2288. Data are presented as mean ± SEM (n=6). Comparisons between 2 groups and among multiple groups were performed using linear mixed-effects models. For multiple-group comparisons, post hoc pairwise comparisons were conducted using the emmeans with Tukey adjustment. *P* values are indicated in the figure.

### Deficiency of STC1 Exacerbates Hypoxia-Induced PH

To determine the in vivo role of STC1, we analyzed *Stc1*^−/−^ mice exposed to chronic hypoxia for 4 weeks (Figure 6A). Representative genotyping data confirming deletion of the *Stc1* allele are shown in Figure S10A. In addition, *Stc1* expression was assessed by quantitative polymerase chain reaction in lung tissues and primary PASMCs isolated from WT and *Stc1*^−/−^ mice. *Stc1* mRNA expression was markedly reduced in *Stc1*^−/−^ lung tissues and was nearly undetectable in *Stc1*^−/−^ PASMCs (Figure S10B). Compared with WT controls, *Stc1*^−/−^ mice exhibited more severe PH, as indicated by increased right ventricular systolic pressure (Figure 6B and 6C) and enhanced pulmonary arterial medial wall thickness (Figure 6D through 6F). Consistently, immunostaining revealed an increased α-SMA (alpha-smooth muscle actin)-positive area under hypoxia, which was further augmented in *Stc1*^−/−^ mice (Figure S11). Staining for von Willebrand factor, an endothelial marker, showed no overt change under conditions of hypoxia or *Stc1* deficiency (Figure S11). These differences occurred despite comparable age, body weight, and tibia length (Table S5). In this model, hypoxia increased the Fulton index (right ventricular/[left ventricular + septum]) in both genotypes; however, no significant difference was observed between WT and *Stc1*^−/−^ mice (Figure 6G). Systemic arterial pressure, left ventricular systolic pressure, and left ventricular ejection fraction were unaffected by either hypoxia or *Stc1* deficiency (Figure 6H through 6J). The same trends were observed when the data were analyzed separately in males and females (Figure S12). Consistent with this, 2-way ANOVA revealed no significant main effect of sex and no significant interaction effects between sex and genotype or oxygen condition (Table S6).

**Figure 6.**
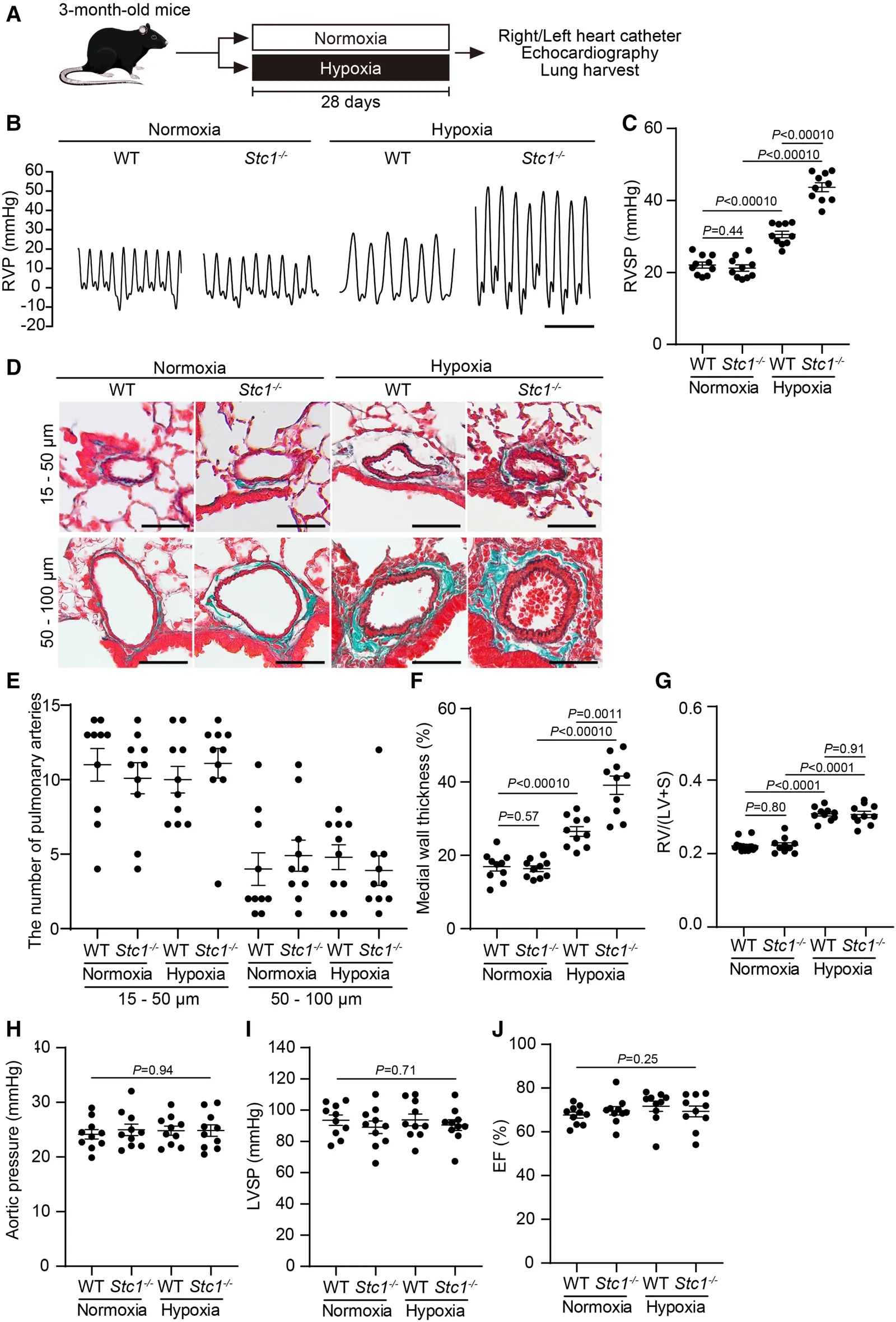
**Loss of STC1 (stanniocalcin-1) promotes the progression of hypoxia-induced pulmonary hypertension in mice. A**, Experimental protocol for generating the hypoxia-induced pulmonary hypertension (PH) model. Wild-type (WT) and *Stc1*-knockout (*Stc1*^−/−^) mice were housed in normoxic (fraction of inspired oxygen [FiO_2_] 21%) or hypoxic (FiO_2_ 10%) chambers for 28 days, followed by physiological and histological assessments. **B**, Representative right ventricular pressure (RVP) waveforms from WT and *Stc1*^−/−^ mice under normoxic or hypoxic conditions. Scale bar, 5 s. **C**, Right ventricular systolic pressure (RVSP) in WT and *Stc1*^−/−^ mice under normoxic or hypoxic conditions (total n=10 per group; 5 males and 5 females). **D**, Representative Elastica-Masson-Goldner-stained lung sections of muscularized pulmonary arteries with external diameters of 15 to 50 µm (**upper**) and 50 to 100 µm (**lower**). Scale bars, 50 µm. **E**, The number of pulmonary arteries included in the quantitative analysis according to vessel size (15–50 µm and 50–100 µm in external diameter). **F**, Quantification of pulmonary vascular remodeling, expressed as the percentage of medial wall thickness relative to total vessel diameter (total n=10 per group; 5 males and 5 females). Right ventricular hypertrophy index (calculated as RV/[LV+S], **G**), aortic pressure (**H**), left ventricular systolic pressure (LVSP, **I**), and left ventricular ejection fraction (EF, **J**) in WT and *Stc1*^−/−^ mice exposed to normoxic or hypoxic conditions (total n=10 per group; 5 males and 5 females). Data are presented as mean ± SEM. Statistical analysis was performed using the Kruskal-Wallis test followed by the Mann-Whitney *U* test. *P* values are indicated in the figure.

To determine whether *Stc1* deficiency promotes cell proliferation in the pulmonary arteries in vivo, the proportion of Ki-67-positive cells in the pulmonary arteries was significantly increased in *Stc1*^−/−^ mice compared with WT controls (Figure 7A and 7B). We next evaluated perivascular macrophage infiltration, which has been reported to increase in PH.^36,37^ CD68-and CD86-positive macrophages, M1 macrophage markers, were increased in *Stc1*^−/−^ mice, even under normoxia, and were further elevated under hypoxia (Figure 7C through 7F). In contrast, CD206-positive macrophages, an M2 macrophage marker, were increased under hypoxia in both genotypes without significant differences between groups (Figure 7G and 7H). Apoptosis was unaffected by either hypoxia or *Stc1* deficiency (Figure S13). Collectively, these findings demonstrate that loss of *Stc1* promotes cell proliferation in the pulmonary arteries and exacerbates the progression of hypoxia-induced PH.

**Figure 7.**
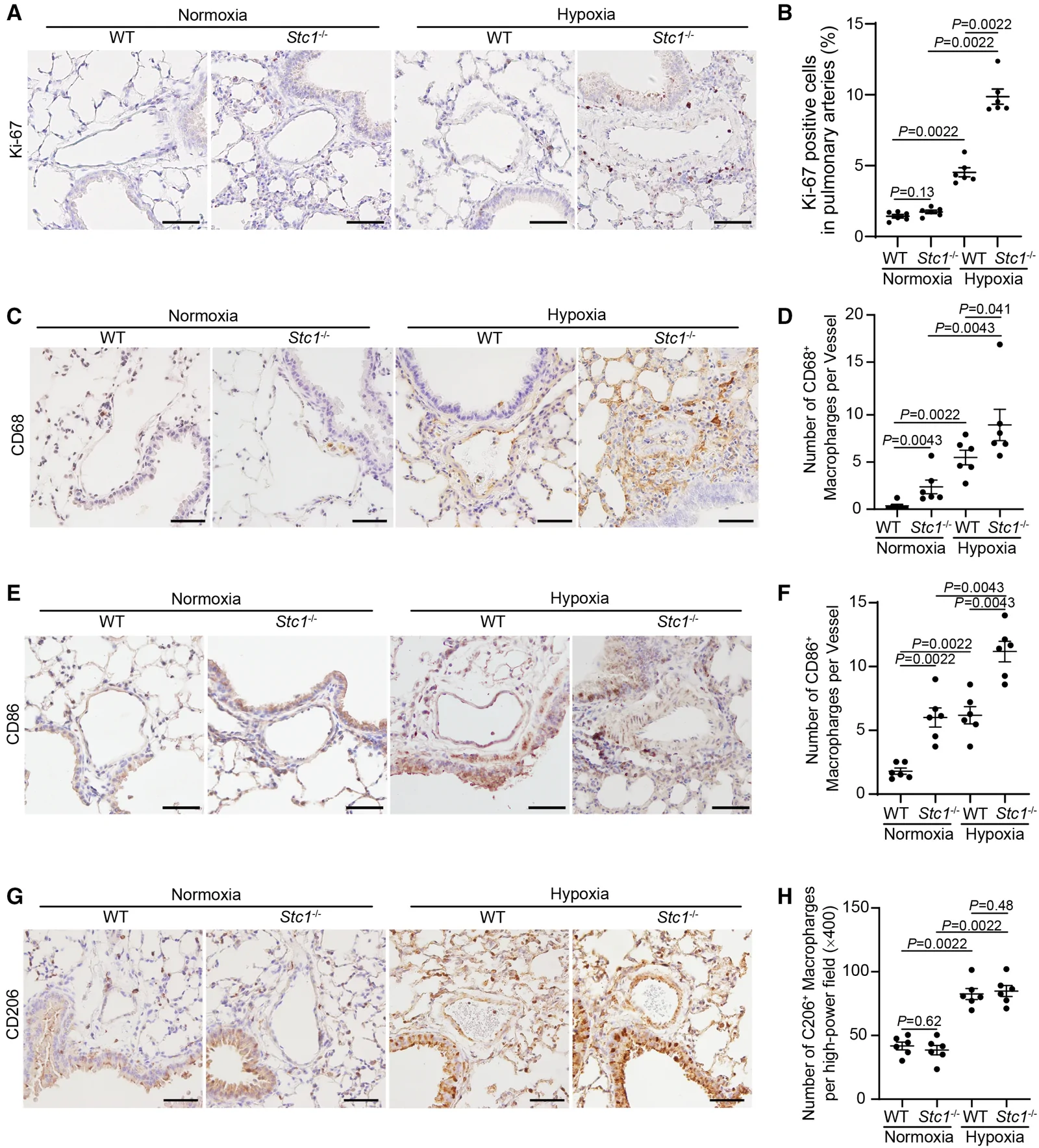
**Proliferative and macrophage marker profiles in wild-type (WT) and *Stc1*^−/−^ mouse lungs under normoxic or hypoxic conditions. A**, Representative immunohistochemistry images of Ki-67 expression in WT and *Stc1*^−/−^ mice exposed to normoxia or hypoxia. **B**, Quantification of Ki-67-positive cells in the pulmonary arteries. **C**, Representative immunohistochemistry staining for CD68 in WT and *Stc1*^−/−^ mice exposed to normoxia or hypoxia. **D**, Quantification of CD68-positive cells per pulmonary artery. **E**, Representative immunohistochemistry staining for CD86 in WT and *Stc1*^−/−^ mice exposed to normoxia or hypoxia. **F**, Quantification of CD86-positive cells per pulmonary artery. **G**, Representative immunohistochemistry staining for CD206 in WT and *Stc1*^−/−^ mice exposed to normoxia or hypoxia. **H**, Quantification of CD206-positive cells per high-power field. Data are presented as mean ± SEM. Statistical significance was determined using the Kruskal-Wallis test followed by the Mann-Whitney *U* test. *P* values are indicated in the figure. Scale bars, 50 µm.

### Recombinant STC1 Attenuates the Progression of Hypoxia-Induced PH

To further evaluate the therapeutic potential of STC1 in PH, rhSTC1 was administered via inhalation every 3 days throughout the hypoxic exposure period in a murine model of hypoxia-induced PH (Figure 8A). Repeated administration of rhSTC1 significantly reduced right ventricular systolic pressure in both WT and *Stc1*^−/−^ mice compared with vehicle controls (Figure 8B and 8C), whereas right ventricular/(left ventricular + septum) was not significantly altered by rhSTC1 treatment (Figure 8D). Consistent with the hemodynamic improvement, pulmonary arterial medial wall thickening was markedly attenuated in both genotypes following rhSTC1 treatment (Figure 8E through 8G), indicating a reduction in vascular remodeling.

**Figure 8.**
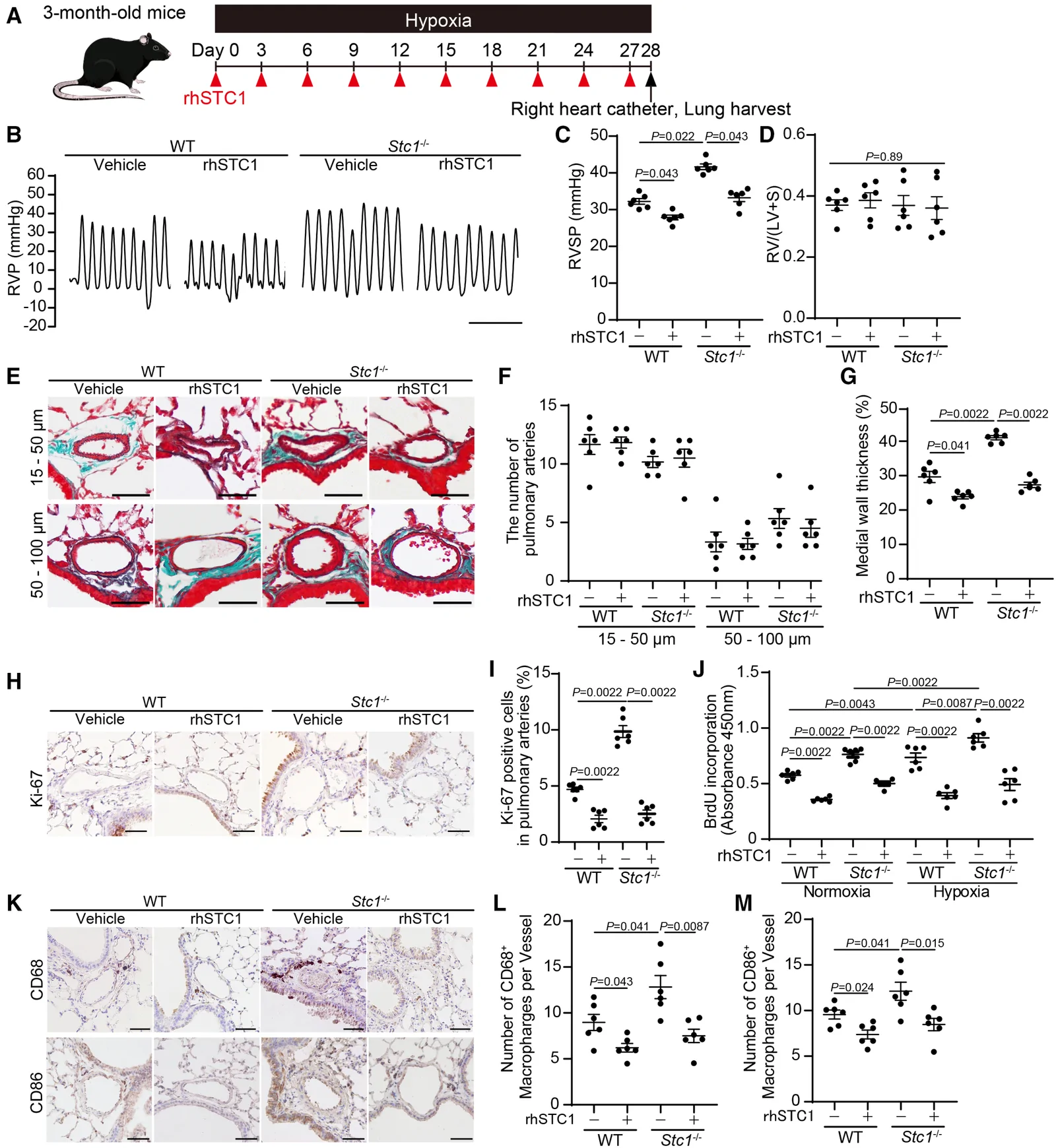
**Recombinant STC1 (stanniocalcin-1) attenuates the pathological features of hypoxia-induced pulmonary hypertension in mice. A**, Experimental protocol for generating the hypoxia-induced pulmonary hypertension (PH) model mice and administering rhSTC1 (recombinant human STC1). Wild-type (WT) and *Stc1*^−/−^ mice were exposed to hypoxic conditions (fraction of inspired oxygen [FiO_2_] 10%) for 28 days. During this period, mice received either rhSTC1 (2 µg/50 µL PBS) or vehicle via inhalation every 3 days. Physiological and histological analyses were subsequently performed. **B**, Representative right ventricular pressure waveforms from WT and *Stc1*^−/−^ mice treated with rhSTC1 or vehicle. Scale bar: 5 s. **C**, Right ventricular systolic pressure (RVSP) in WT and *Stc1*^−/−^ mice treated with rhSTC1 or vehicle (n=6). **D**, Right ventricular hypertrophy index (calculated as RV/[LV+S]) in WT and *Stc1*^−/−^ mice treated with rhSTC1 or vehicle (n=6). **E**, Representative Elastica-Masson-Goldner-stained lung sections of WT and *Stc1*^−/−^ mice treated with rhSTC1 or vehicle. The pulmonary arteries with external diameters of 15–50 µm (**upper**) and 50–100 µm (**lower**) are shown. Scale bars, 50 µm. **F**, The number of pulmonary arteries included in the quantitative analysis according to vessel size (15–50 µm and 50–100 µm in external diameter). **G**, Quantification of pulmonary vascular remodeling by calculating the percentage of medial wall thickness relative to total vessel diameter in WT and *Stc1*^−/−^ mice treated with rhSTC1 or vehicle (n=6). **H**, Representative immunohistochemistry images of Ki-67 expression in WT and *Stc1*^−/−^ mice treated with rhSTC1 or vehicle. **I**, Quantification of Ki-67-positive cells in the pulmonary arteries. **J**, 5-Bromo-2′-deoxyuridine (BrdU) incorporation in cultured primary PASMCs isolated from hypoxia-induced PH model mice following treatment with or without rhSTC1 (100 ng/mL). **K**, Representative immunohistochemistry staining for CD68 and CD86 in WT and *Stc1*^−/−^ mice treated with rhSTC1 or vehicle. **L**, Quantification of CD68-positive cells per pulmonary artery. **M**, Quantification of CD86-positive cells per pulmonary artery. Data are presented as mean ± SEM. Statistical analysis was performed using Kruskal-Wallis test followed by Mann-Whitney *U* test. *P* values are indicated in the figure. Scale bars, 50 µm.

To investigate whether the observed structural improvement was accompanied by suppression of cell proliferation, the proportion of Ki-67-positive cells in the pulmonary arteries was significantly decreased in rhSTC1-treated groups in both WT and *Stc1*^−/−^ mice (Figure 8H and 8I), demonstrating that exogenous rhSTC1 effectively suppresses proliferative activity under hypoxic conditions. Consistently, treatment with rhSTC1 reduced BrdU incorporation in primary PASMCs isolated from WT and *Stc1*^−/−^ mice under both normoxic and hypoxic conditions (Figure 8J).

The inflammatory component of pulmonary vascular remodeling was examined by assessing the perivascular infiltration of CD68- or CD86-positive macrophages. rhSTC1 administration markedly reduced macrophage accumulation in both WT and *Stc1*^−/−^ lungs (Figure 8K through 8M), suggesting that STC1 exerts anti-inflammatory effects in addition to its antiproliferative action. Collectively, these findings demonstrate that exogenous rhSTC1 inhalation ameliorates the hypoxia-induced PH phenotype in *Stc1*^−/−^ mice and alleviates pulmonary vascular remodeling and right ventricular pressure overload in WT mice.

## Discussion

In the present study, we applied a custom-designed hydrostatic pressure apparatus capable of reproducing the pressure oscillations observed in the pulmonary arteries of patients with PAH. Transcriptomic analysis of IPAH-derived PASMCs subjected to this system revealed significant enrichment of multiple cell cycle–related gene sets by gene set enrichment analysis. These findings confirm that our apparatus is a platform that, at least in part, recapitulates the mechanical environment of PAH. Using this platform, we identified STC1 as a mechanosensitive molecule induced via PIEZO1-HIF-1α signaling, which exerted antiproliferative and protective effects in IPAH PASMCs. Furthermore, in a hypoxia-induced PAH model, *Stc1* deficiency aggravated disease severity, whereas supplementation ameliorated pathological remodeling, suggesting that STC1 may represent a novel therapeutic target in PAH.

Previous studies have established PIEZO1 as a critical mechanosensor in vascular cells,^38^ capable of transducing mechanical stimuli into downstream signals such as Ca^2+^ influx and HIF-1α activation.^25,26^ However, the downstream effectors of PIEZO1 in PAH have remained largely undefined. In this study, we identified STC1 as a novel effector induced in a PIEZO1-HIF-1α–dependent manner in PASMCs derived from patients with IPAH. STC1 has been implicated in cell proliferation, oxidative stress regulation, and mitochondrial homeostasis in cancer cells and renal cells,^23,39^ but its role in vascular smooth muscle cells or in PH has not been previously elucidated. Importantly, the absence of STC1 induction by hydrostatic pressure in non-PAH PASMCs suggests that disease-specific contexts may amplify mechanosensitive transcriptional responses. In particular, the upregulation of PIEZO1 observed in IPAH^40,41^ may potentiate exaggerated responses to hydrostatic stress. Collectively, these findings extend our understanding of hydrostatic pressure-responsive signaling in PAH and establish STC1 as a previously unrecognized protective mediator.

The finding that rhSTC1 induced cell cycle arrest and suppressed pulmonary vascular remodeling supports the concept that increased STC1 may function as a protective mechanism against pathological remodeling. In human lung tissues, STC1 expression was elevated in IPAH compared with donors; however, the proliferation marker Ki-67 was also increased. Consistent with these observations, multiple lines of evidence from the present study support the notion that STC1 upregulation represents an adaptive response rather than a maladaptive driver of disease progression. Genetic deletion of *Stc1* exacerbated disease severity in vivo, whereas exogenous STC1 supplementation ameliorated pulmonary vascular remodeling. In addition, *Stc1* expression was increased in the experimental PAH model, and small interfering RNA-mediated knockdown of *STC1* enhanced PASMC proliferation, indicating that endogenous STC1 functions to restrain cell growth. Collectively, these findings support a model in which STC1 is induced in response to pathological pressure overload as a compensatory, protective mechanism. Indeed, circulating STC1 levels have been recently reported to predict adverse outcomes in patients with IPAH.^42^ Taken together, these findings indicate that STC1 is increased as a reflection of disease progression but exerts protective effects against pathological remodeling, and that exogenous supplementation of STC1 may represent a novel therapeutic strategy for PAH.

STC1 suppressed PASMC proliferation and attenuated PAH progression in vivo, indicating its protective role in pulmonary vascular remodeling. Therapeutic enhancement of STC1 signaling could be achieved either by supplementation of STC1 or by upstream modulation of PIEZO1-HIF-1α activity. However, PIEZO1 and HIF-1α pathways exert pleiotropic functions in vivo,^43–45^ particularly in the vasculature, where they promote remodeling to maintain oxygen delivery and vascular tone.^45^ Supplementation of STC1, a downstream effector, may represent a more specific strategy. Our in vitro data demonstrate a direct antiproliferative effect of STC1 on PASMCs isolated from a mouse model of hypoxia-induced PH. These findings support the beneficial role of STC1 in PASMCs; however, the in vivo phenotypes of global STC1 deficiency likely reflect integrated effects involving multiple vascular and nonvascular cell types. For instance, STC1 has been shown to be abundantly expressed in endothelial cells.^46^ The contribution of cell types other than PASMCs cannot be excluded as targets of STC1 supplementation.

IGF2R (insulin-like growth factor 2 receptor) has been suggested as a binding partner, but it primarily functions in IGF2 (insulin-like growth factor 2) clearance and TGF-β (transforming growth factor β) regulation and is not directly implicated in the control of vascular tone.^47^ Therefore, STC1 is more likely to target proliferative and remodeling processes in PAH, potentially complementing existing vasodilator therapies. However, the precise receptors and downstream signaling network of STC1 remain incompletely defined, particularly in the context of mechanically stressed PASMCs, and warrant further investigation.

Il-6 (Interleukin-6), a central inflammatory cytokine in PAH,^48^ is produced by M1 macrophages, which have been reported to be increased in PAH.^36,37^ STC1 has been shown to exert inhibitory effects on IL-6 upregulation^49^ and, in the present study, STC1 suppressed perivascular infiltration of M1 macrophages. Furthermore, STC1 has been reported to suppress fibrosis induced by TGF-β, a major driver of vascular remodeling.^39^ Taken together, these findings suggest that STC1 functions not only as an antiproliferative factor in PASMCs but also as a regulator of anti-inflammatory and antifibrotic pathways, highlighting its multifaceted therapeutic potential in PAH.

Several limitations warrant consideration. First, although we demonstrated that PIEZO1-HIF-1α signaling mediates STC1 induction, the full transcriptional network upstream of STC1 remains to be elucidated. Second, the number of donor-derived PASMC lines was small, and interpatient variability may influence mechanosensitive transcriptional responses. In this cohort, samples were selected to minimize sex imbalance between groups (non-PAH: 7 male and 6 female; IPAH: 6 male and 5 female). In addition, noncancerous lung tissues were used as non-PAH controls to better reflect physiological conditions and to minimize potential confounding effects associated with malignancy. However, strict age matching was not feasible due to the relatively younger age of the IPAH cohort and the limited availability of appropriate non-PAH control samples. Third, our hydrostatic pressure apparatus and hypoxia-induced PH model capture many aspects of PAH but do not fully replicate the complex hemodynamic and molecular environment of human IPAH. The hydrostatic pressure conditions used in this study fall within the range of pulmonary arterial pressures reported in patients with IPAH,^50^ supporting the physiological relevance of our experimental system. However, it should be noted that the in vivo pulmonary arterial pressure waveform is more complex, and our model does not fully recapitulate dynamic factors such as vascular stretch, shear stress, or spatial heterogeneity of pressure. In addition, the chronic hypoxia model used in this study represents a relatively mild form of PH that does not develop plexiform lesions. Previous studies have shown that 4 weeks of hypoxia induces a moderate increase in pulmonary arterial pressure but does not always result in marked right ventricular hypertrophy. Consistent with this, although an increase in right ventricular pressure was observed in our model, the magnitude and duration of hypoxic exposure may not have been sufficient to produce a significant difference in right ventricular/(left ventricular + septum). In addition, the potential development of antibodies against rhSTC1 during repeated administration cannot be excluded and may influence treatment efficacy in vivo. Finally, mechanosensitive channels other than PIEZO1 or integrin-mediated pathways may contribute to STC1 induction.

In conclusion, we identified the PIEZO1-HIF-1α–STC1 axis as a novel mechanosensitive pathway in IPAH PASMCs. Using a custom hydrostatic pressure apparatus that replicates disease-relevant mechanical stress, we demonstrated that STC1 is upregulated in response to pathological pressure and exerts antiproliferative, anti-inflammatory, and protective effects against pulmonary vascular remodeling. These findings provide new mechanistic insight into how abnormal hemodynamic forces contribute to PAH and highlight STC1 as a promising therapeutic target.

## Article Information

### Acknowledgments

The authors thank Ms Yuka Sawada and Dr Tomoko Furuta (Department of Physiology, Tokyo Medical University) for their technical assistance. The authors are also deeply grateful to Drs Hiromu Tanaka, Hideto Iizuka, and Koichi Fukunaga (Department of Respiratory Medicine, Keio University School of Medicine) for providing expert instruction on intratracheal drug administration in mice. The generation of the custom-built periodic hydrostatic pressure culture system was aided by Koganei Co., Ltd (Tokyo, Japan).

### Author Contributions

Study conception and design: U. Yokoyama and M. Kogami. Data acquisition: M. Kogami, Y. Kato, S. Ito, H. Inoue, Y. Hidaka, S. Tanifuji, M. Yokotsuka, and Y. Yamamoto. Analysis and interpretation: M. Kogami, U. Yokoyama, K. Uchida, T. Nagao, S. Abe, R.R. Reddel, and K. Nakamura. Drafting of manuscript: M. Kogami and U. Yokoyama. Critical revision: U. Yokoyama. Final approval of the version to be published: All authors.

### Disclosures

None.

### Supplemental Material

Supplemental Methods

Tables S1–S6

Figures S1–S13

## Supplementary Material
