# Supplementary material for: Mechanosensitive Stanniocalcin-1 Suppresses Smooth Muscle Cell Proliferation and Attenuates Experimental Pulmonary Hypertension
Source: Circ Res. 2026 Sep 9;139(9):e327869. doi: 10.1161/CIRCRESAHA.125.327869 (PMC13649279; doi:10.1161/CIRCRESAHA.125.327869)
Supplement: Supplementary file 1 [file res-139-e327869-s001.pdf]

Supplemental Methods

Human Samples and Cell Culture

Pulmonary arterial smooth muscle cells (PASMCs, n=4) and lung tissues (n=11 [6 male, 5 female]) were isolated from intrapulmonary arteries of explanted lungs from patients with severe idiopathic pulmonary arterial hypertension (IPAH) undergoing lung transplantation at Okayama University (Major Resources Table). Non-PAH lung tissues without lung cancer were obtained from Tokyo Medical University (n=13 [7 male, 6 female]). Non-PAH PASMCs were obtained from surplus lung tissue during surgery in non-PAH patients at Okayama University (Major Resources Table). Non-PAH-6, -7, -8 PASMCs (CC-2581) were purchased from Lonza (Basel, Switzerland). PASMCs from Okayama University were cultured in Dulbecco's modified Eagle medium (DMEM; Nacalai Tesque, Kyoto, Japan, Cat. No. 08458-45) containing 10% fetal bovine serum (FBS; Gibco/Thermo Fisher Scientific, Waltham, MA, USA, Cat. No. A5256701) and 1% penicillin–streptomycin (Nacalai Tesque, Cat. No. 26253-84). Non-PAH-6, -7, -8 PASMCs were cultured in Smooth Muscle Growth Medium-2 (SmGM-2; Lonza) containing 5% FBS and 1% penicillin–streptomycin. All cells were incubated at 37°C in a humidified atmosphere containing 5% CO_2, and passages 3 to10 were used for

experiments. Donor age, sex, diagnosis, sample type (tissue or cells), vendor/source, and passage numbers are summarized in the Major Resources Table.

RNA Sequencing

Total RNA was extracted from IPAH-PASMCs using the RNeasy Mini Kit (Qiagen, Hilden, Germany, Cat. No. 74104) following the manufacturer's protocol. RNA concentration and purity were assessed with a NanoDrop 2000 spectrophotometer (Thermo Fisher Scientific, Cat. No. ND-2000). From RNA integrity assessment onward, library preparation, sequencing, and primary data processing were performed by Rhelixa Inc. (Tokyo, Japan). RNA integrity was evaluated using an Agilent 2100 Bioanalyzer (Agilent Technologies, Santa Clara, CA, USA, Cat. No. G2939BA), and samples with RNA integrity number (RIN) ≥7.0 were selected for sequencing. mRNA was enriched using poly(A) selection (TruSeq Stranded mRNA Library Prep Kit; Illumina, San Diego, CA, USA, Cat. No. RS-122-2102). Libraries were indexed and quantified with quantitative PCR, pooled, and sequenced on an Illumina NovaSeq X Plus platform (Illumina, Cat. No. 20084804) to generate paired-end 150-bp reads. The target sequencing depth was 26.7 million reads per sample.

Base calling and demultiplexing were performed with bcl2fastq (Illumina). Raw FASTQ files underwent quality control using FastQC (v0.11.7; Babraham Bioinformatics, Cambridge, UK) and adapter trimming with Trimmomatic (v0.38; Usadel Lab, Aachen, Germany). Reads were aligned to the GRCh38 reference genome using HISAT2 (v2.1.0) with default parameters, and gene-level quantification was performed with featureCounts (v1.6.3).

Differential expression analysis was carried out in R (v4.2.2; R Foundation for Statistical Computing, Vienna, Austria) using DESeq2 (Bioconductor). MA plots were generated using the plotMA function in DESeq2 to visualize log2 fold changes versus mean normalized counts. As no genes met the predefined statistical significance threshold in the paired differential expression analysis, among annotated genes with a baseMean ≥ 50 were used for subsequent exploratory analyses to reduce the influence of unstable fold changes derived from low-abundance transcripts. Gene set enrichment analysis was performed using these genes, ranked by log2 fold change, with fgsea using gene sets from the Molecular Signatures Database (MSigDB, Broad Institute, Cambridge, MA, USA). Enrichment results were visualized and functionally clustered using clusterProfiler and enrichplot, followed by automated term summarization with

dplyr and tidytext. Transcription factor enrichment analysis was conducted with the iRegulon plugin in Cytoscape (v3.10.3; National Institute of General Medical Sciences, Bethesda, MD, USA) using default motif collection settings. Upregulated (log2 fold change > 0.25) and downregulated (log2 fold change < −0.25) gene sets (highlighted in Figure 1D; listed in Tables S3 and S4) were analyzed. Significant regulators were defined as those with a normalized enrichment score (NES) >3 and area under the curve (AUC) >0.03.

Animals

Mice were housed under controlled environmental conditions (22 ± 2°C, 12-h light/dark cycle) with free access to food and water. Generation of *Stc1*$^{-/-}$ mice by homologous recombination has been described previously.[9] All mice were littermates derived from heterozygote crosses on a C57BL/6J genetic background.

Genotyping was performed by polymerase chain reaction (PCR) using a wild-type (WT)-specific forward primer 5′-AGCGCACGAGGCGGAACAAA-3′, WT-specific reverse primer 5′-AGAGAGCCGCTGTGAGGCGT-3′, mutant-specific forward primer 5′-AAAAGCCAGAGGTGCAAGAA-3′, and mutant-specific reverse primer 5′-

TATGATCGGAATTCCTCGAC-3′. The WT STC1 allele yielded a 403-bp amplicon, and the mutant allele yielded a 316-bp amplicon. PCR cycling conditions were as follows: 30 cycles of 94°C for 45 s, 58°C for 30 s, and 72°C for 60 s, using a thermal cycler (Applied Biosystems, Foster City, CA, USA).

Hypoxia-Induced PH Model Mice

WT and *Stc1*$^{-/-}$ mice were maintained under normoxic conditions (21% O_2) or exposed to hypoxia (10% O_2) in a hypoxic chamber (ProOx360; BioSpherix, Parish, NY, USA) for 4 weeks, with free access to food and water. The age, body weight, and tibia length of the mice used in this study are summarized in Table S5.

Hypoxia-Induced PH Model With rhSTC1 Administration

Intratracheal inhalation was performed via 24G Surflo catheter intubation (Terumo, Tokyo, Japan, SR-OX2419CA). The rhSTC1 (R&D Systems, Minneapolis, MN, USA; Cat. No. 9400-SO) dose (2 μg/50 μL PBS) was determined based on previous reports of intratracheal administration.[43] In a pilot pharmacokinetic study, *Stc1*$^{-/-}$ mice received a single intratracheal dose of rhSTC1, and whole lungs were collected at predetermined

time points (12, 48, 96, and 168 h) after administration. Lungs were perfused with 2.5 mL carbonate buffer, and lung tissue STC1 concentrations were quantified by ELISA (R&D Systems, Cat. No. DY2958) according to the manufacturer's instructions. The elimination half-life was estimated from a regression curve, and the dosing interval was set at every 72 h (Figure S2).

For the therapeutic protocol, WT and *Stc1*$^{-/-}$ mice were administered either rhSTC1 or vehicle control (50 μL PBS) and placed in a hypoxic chamber (10% O_2) for 4 weeks, with free access to food and water. During hypoxia exposure, rhSTC1 was administered every 72 h.

Hemodynamics Measurements

Echocardiography was performed using an ultrasonography system (SSA-700A Diagnostic Ultrasound System; Toshiba, Tokyo, Japan) under 2% isoflurane anesthesia with mice placed on a heating pad. Heart rates were maintained at 400–600 bpm. Short-axis views were captured using B-mode imaging, and M-mode recordings were obtained with a two-dimensional reference sector for left ventricular (LV) dimensions. Ultrasound image analysis was performed using the Pombo method with ImageJ software (National

Institutes of Health, Bethesda, MD, USA). All measurements were averaged over 3 cardiac cycles.

On the following day, invasive left and right heart catheterization was performed under 2% isoflurane anesthesia. Body temperature was maintained using a heating pad, and heart rate was monitored and kept between 400 and 600 bpm. The surgical field was sterilized with 70% ethanol. A pressure catheter (SPR-1000; Millar Instruments, Houston, TX, USA) was introduced into the right ventricle (RV) via the right jugular vein and right atrium to measure RV pressure (RVP). The right common carotid artery was then exposed, and the same catheter was advanced retrogradely into the LV to record aortic and LV pressures (LVP). Pressure signals were recorded using a PowerLab 8/35 data acquisition system (ADInstruments, Colorado Springs, CO, USA). Hemodynamic parameters were averaged over 5 consecutive cardiac cycles. Animals in which catheterization failed or hemodynamic recordings were unstable were excluded from further analysis.

Tissue Collection

After completion of hemodynamic measurements, mice were euthanized by

exsanguination. Lungs were perfused via the RV with 10 mL of cold PBS to collect tissue for RNA extraction. For immunohistochemistry, lungs were further perfused via the RV with 5 mL of 10% neutral-buffered formalin (Nacalai Tesque, Cat. No. 37152-51). After complete perfusion, 10% neutral-buffered formalin was instilled intratracheally at a pressure equivalent to 25 cm H_2O, and the trachea was ligated. Lungs were fixed overnight in formalin, transferred to 70% ethanol, and paraffin-embedded. Hearts were dissected free of atria and great vessels, and RV was separated from the left ventricle plus septum (LV+S) to calculate the Fulton index (RV/[LV+S]).

Tissue Staining and Evaluation of Medial Wall Thickness of Pulmonary Arteries

Paraffin-embedded lung tissue blocks were sectioned at 4 μm thickness and mounted on MAS-coated glass slides (Matsunami Glass Ind., Osaka, Japan, Cat. No. MAS-04). To visualize the structural characteristics of the vessel wall, paraffin-embedded lung sections were stained using the Elastica–Masson–Goldner (EMG) method. Briefly, sections were deparaffinized in xylene and rehydrated through a graded ethanol series to distilled water, followed by incubation in 3% potassium dichromate (FUJIFILM Wako Pure Chemical, Osaka, Japan, Cat. No. 166-03655) overnight. Elastic fibers were

stained with Weigert's resorcin–fuchsin solution (Muto Pure Chemicals, Tokyo, Japan, Cat. No. 41081) for 60 min, followed by differentiation in 70% ethanol containing 1% hydrochloric acid until the background became clear. Nuclei were stained with Carazzi's hematoxylin (Muto Pure Chemicals, Cat. No. 30022) for 5 min, and then rinsed in running tap water for 5 min. Cytoplasm and muscle fibers were stained with ponceau–acid fuchsin solution (Muto Pure Chemicals, Cat. No. 18461) for 15 min, followed by brief differentiation in 0.2% acetic acid solution. Sections were then treated with phosphotungstic acid–orange G solution (Muto Pure Chemicals, Cat. No. 20951) for 15 min as a mordant, after which collagen fibers were stained with light green solution (Muto Pure Chemicals, Cat. no. 16732) for 7 min, followed by brief differentiation in 0.2% acetic acid solution. Finally, sections were dehydrated through graded ethanol, cleared in xylene, and coverslipped with mounting medium (PathoMount; FUJIFILM Wako Pure Chemical Corporation, Cat. No. 168-28495).

Medial wall thickness was evaluated from EMG-stained images by measuring 15 small pulmonary arteries with an external diameter 15–100 μm, accompanying non-cartilaginous bronchioles and sharing a common adventitial sheath, per sample. The external and internal elastic lamina diameters were measured using ImageJ software

(v1.53; National Institutes of Health), and medial wall thickness was calculated as (external elastic lamina diameter − internal elastic lamina diameter) / external elastic lamina diameter × 100 (%). The average value of 15 pulmonary arteries per sample was used as the representative measurement for statistical analysis. All measurements were performed in a blinded manner, without knowledge of the group allocation of each mouse.

Immunohistochemistry of Human Lung Tissue

Immunohistochemical staining for STC1 and PIEZO1 was performed on paraffin-embedded human lung tissue sections. Sections were deparaffinized in xylene, rehydrated through a graded ethanol series, and incubated in 0.3% hydrogen peroxide solution (prepared from hydrogen peroxide, FUJIFILM Wako Pure Chemical Corporation, Cat. No. 081-04215) for 30 min to inactivate endogenous peroxidase activity. Antigen retrieval was performed by incubating the sections in 1 mM EDTA buffer (pH 9.0) at 95°C for 30 min. After cooling to room temperature, nonspecific binding was blocked by incubating the sections with 1.5% normal serum from the VECTASTAIN Elite ABC-HRP Kits (Vector Laboratories, Burlingame, CA, USA; Cat. Nos. PK-6101, PK-

6104, and PK-6105) were used according to the species of the primary antibodies for 30 min. Sections were then incubated with primary antibodies against STC1 (Abcam, Cambridge, UK; Cat. No. ab229477), PIEZO1 (Proteintech, Rosemont, IL, USA; Cat. No. 15939-1-AP), or Ki-67 (Abcam, Cat. No. ab16667) at 4°C overnight (antibody details are provided in Major Resources Table). Negative control sections were processed in parallel using the corresponding secondary antibodies alone, with omission of the primary antibodies.

Following 3 washes in 0.1 M PBS for 5 min each, the sections were incubated with biotinylated anti-rabbit IgG secondary antibody (Vector Laboratories, Cat. No. PK-6101) for 30 min at room temperature, followed by incubation with avidin-biotin-peroxidase complex from the same kit for an additional 30 min. Immunoreactivity was visualized using 3,3'-diaminobenzidine (DAB) solution from the Peroxidase Stain DAB Kit (Nacalai Tesque, Cat. No. 25985-50). After color development, the sections were counterstained with Mayer's hematoxylin (FUJIFILM Wako Pure Chemical Corp., Cat. No. 131-09665) for 1 min, rinsed in running tap water, dehydrated through graded ethanol, cleared in xylene, and coverslipped with mounting medium.

Quantification of immunostaining was performed using ImageJ software (NIH). Bright-

field images were acquired under identical exposure settings. The medial layer of pulmonary arteries was defined as the region of interest (ROI). DAB signals were extracted using the Color Deconvolution plugin, and staining intensity was quantified as mean optical density (OD). Five pulmonary arteries per section were analyzed, and the values were averaged for each sample.

Immunohistochemistry of Mouse Lung Tissue

Immunohistochemical staining for α-smooth muscle actin (α-SMA; Sigma-Aldrich, St. Louis, MO, USA; Cat. No. A2547), von Willebrand factor (vWF; Dako, Glostrup, Denmark; Cat. No. A0082), CD68 (Bio-Rad, Hercules, CA, USA; Cat. No. MCA1957), CD86 (R&D Systems, Minneapolis, MN, USA; Cat. No. AF-141-NA), CD206 (Abcam, Cambridge, UK; Cat. No. ab64693), Ki-67 (Abcam, Cat. No. ab16667) was performed on paraffin-embedded mouse lung tissue sections. Sections were deparaffinized in xylene, rehydrated through a graded ethanol series, and incubated in 0.3% hydrogen peroxide solution for 30 min to inactivate endogenous peroxidase activity.

For antigen retrieval, sections for vWF and CD68 staining were incubated in 20 μg/mL proteinase K (TaKaRa Bio Inc., Cat. No. 9034) for 10 min at 37°C, while sections

for α-SMA and CD206 staining were incubated in 1 mM EDTA buffer (pH 9.0) for 30 min at 95°C. After cooling to room temperature, nonspecific binding was blocked with 1.5% normal serum from the Vectastain Elite ABC IgG Kit for 30 min. Sections were then incubated overnight at 4°C with primary antibodies (details in Major Resources Table). Negative control sections were processed in parallel using the corresponding secondary antibodies alone, with omission of the primary antibodies.

Following 3 washes in 0.1 M PBS for 5 min each, the sections were incubated with biotinylated anti-mouse, anti-rabbit, or anti-rat IgG secondary antibodies for 30 min at room temperature, followed by avidin–biotin–peroxidase complex from the same kit for an additional 30 min. Immunoreactivity was visualized using DAB solution from the Peroxidase Stain DAB Kit. After color development, the sections were counterstained with Mayer's hematoxylin for 1 min, rinsed in running tap water, dehydrated through graded ethanol, cleared in xylene, and coverslipped with mounting medium.

Evaluation of CD68, CD86 and CD206 Expression

The expression level of CD68 and CD86 in lung tissue was quantified from immunohistochemical staining images by counting positive cells surrounding 15

pulmonary arteries (external diameter 15–100 μm) per sample. The mean value of these counts was used as the representative value for each sample.

For CD206 expression, positively stained cells were counted at ×400 magnification in 1 alveolar field and 4 perivascular fields surrounding small pulmonary arteries per sample, for a total of 5 fields. The average number of CD206-positive cells across these fields was taken as the representative value for each sample.

Quantification of Ki-67-Positive Cells in the Pulmonary Arteries

The percentage of Ki-67-positive cells in pulmonary arteries was determined from immunohistochemical staining of lung sections. For mouse samples, 15 pulmonary arteries (external diameter 15–100 μm) were analyzed per sample. For human samples, 5 pulmonary arteries (external diameter <300 μm) were analyzed per sample. Ki-67-positive nuclei within the pulmonary arterial wall were counted, and the percentage of Ki-67-positive cells was calculated by dividing the number of Ki-67-positive nuclei by the total number of nuclei in the corresponding pulmonary arterial wall and multiplying by 100.

Immunocytochemistry for HIF-1α

PASMCs were seeded on glass coverslips and cultured under the indicated conditions. Cells were fixed with 10% neutral buffered formalin, permeabilized with 0.1% Triton X-100, and blocked with 3% bovine serum albumin (BSA). Cells were then incubated with anti-HIF-1α primary antibody (Novus Biologicals, Centennial, CO, USA, Cat. No. NB100-105) overnight at 4°C, followed by incubation with a fluorescently labeled secondary antibody (Alexa Fluor 488 anti-rabbit IgG; Invitrogen, Cat. No. A-11008). Nuclei were counterstained with Hoechst 33258. Negative control sections were processed in parallel using the corresponding secondary antibodies alone, with omission of the primary antibodies.

Fluorescence images were acquired under identical imaging settings. For quantification, nuclear and cytoplasmic regions were defined using ImageJ software. The cytoplasmic region was determined by subtracting the nuclear area from the total cell area. The nuclear-to-cytoplasmic ratio of HIF-1α fluorescence intensity was calculated. Five fields per sample were analyzed, and the values were averaged for each sample.

TUNEL Staining

TdT-mediated dUTP nick end labeling (TUNEL) staining was performed on mouse lung tissue to evaluate apoptotic cells using the TUNEL kit (Promega Corporation, Madison, WI, USA, Cat. No. G7130). All procedures were conducted according to the manufacturer's instructions.

RNA Extraction

RNA extraction from PASMCs or lung tissues was performed to quantify RNA levels. PASMCs were seeded in 35-mm dishes at a density of 1.5×10^5 cells/dish. For knockdown of STC1 or PIEZO1, the medium was replaced the following day with antibiotic-free medium, and cells were transfected with 25 nM siRNA. Control siRNA was used for the control group. After 12 h, the medium was replaced, and cells were harvested at 72 h to confirm knockdown efficiency. For pressure stimulation after knockdown, dishes were placed in the pressure chamber 12 h after transfection and harvested at 72 h. Prior to drug stimulation, cells were serum-starved in DMEM without FBS for 24 h. Cells were then treated with GSK1016790A (10 nmol/L; Tocris Bioscience, Bristol, UK, Cat. No. 2939), naltriben (20 μmol/L; Sigma-Aldrich, Cat. No. SML0882), Yoda1 (20 μmol/L; Cayman Chemical, Ann Arbor, MI, USA, Cat. No. 21904), chetomin

(100 nmol/L; Cayman Chemical, Cat. No. 9004661) + Yoda1 (20 μmol/L), echinomycin (30 nmol/L; Cayman Chemical, Cat. No. 11450) + Yoda1 (20 μmol/L), or belzutifan (10 nmol/L; Cayman Chemical, Cat. No. 28899) + Yoda1 (20 μmol/L) for 24 h; control cells received DMSO at the same concentration as the drugs. The concentrations of each drug were determined based on their reported EC50 values. Cells were collected using RNAisoPlus (Takara Bio, Cat. No. 9109). For lung tissues, samples were snap-frozen in liquid nitrogen, mechanically pulverized, and collected in RNAisoPlus, followed by further disruption using a Bioruptor (Diagenode, Liège, Belgium). RNA extraction was performed according to the manufacturer’s instructions.

Quantitative Reverse Transcription-PCR (RT-PCR)

RT-PCR was performed to quantify mRNA expression levels. Reverse transcription was carried out using the PrimeScript RT Reagent Kit (TaKaRa Bio, Cat. No. RR037A). RT-PCR was performed with TB Green FAST qPCR Mix (TaKaRa Bio, Cat. No. RR430A) on a thermal cycler, with each cycle consisting of denaturation at 95°C for 5 s and annealing/extension at 60°C for 10 s, for a total of 40 cycles. Gene expression levels were normalized to 18S ribosomal RNA using the ΔΔCT method. Primer sequences are

listed in Major Resources Table.

Chromatin Immunoprecipitation (ChIP)-qPCR

ChIP assays were performed using Protein G magnetic beads (Dynabeads; Invitrogen, Cat. No. 10004D) according to the manufacturer's instructions. PASMCs were subjected to cyclic hydrostatic pressure for 3 h. For positive control experiments, cells were treated with deferoxamine (DFO, 100 μmol/L; Sigma-Aldrich, Cat. No. D9533) for 6 h. Cells were crosslinked with 1% formaldehyde for 10 min at room temperature, and the reaction was quenched by replacing the medium with cold serum-containing medium. Cells were harvested and lysed to isolate nuclei. Chromatin was then sheared using a Covaris sonicator (Covaris, Woburn, MA, USA) to an average fragment size of 200–500 bp (peak power 105 W, duty factor 5%, 200 cycles per burst, duration 300 s). Immunoprecipitation was performed using an anti–HIF-1α antibody, with normal mouse IgG2b isotype control (R&D Systems, Cat. No. MAB0041) used as a negative control. Immune complexes were captured using Protein G magnetic beads. After washing, chromatin was eluted and crosslinks were reversed by incubation at 65°C overnight, followed by DNA purification. The enriched DNA fragments were analyzed by qPCR

using primers targeting the STC1 promoter. The VEGFA promoter was used as a positive control. Data were normalized to input DNA.

Western Blotting

Western blotting was performed to assess the basal secretion levels of STC1 and to investigate the effects of STC1 on cell cycle regulation. For secretion analysis, PASMCs were plated in 35-mm dishes at a density of 1.5×10^5 cells/well. When the cultures reached approximately 80% confluency, the cells were serum-starved in DMEM without FBS for 24 h, and the culture supernatants were collected. Supernatants (2 mL) were concentrated to ≤50 μL using a 10-K Amicon Ultra-2 mL Centrifugal Filter (Millipore, Burlington, MA, USA, Cat. No. UFC201024).

For cell cycle analysis, PASMCs were plated under the same conditions, and when ~80% confluency was achieved, rhSTC1 (100 ng/mL) was added; the concentration was selected based on endogenous STC1 levels measured in the culture supernatant of IPAH-derived PASMCs to approximate physiologically relevant conditions (Figure 2C-D). Cells were harvested in carbonate buffer at the designated time points (0, 15, 30, 60, 120, and 360 min, and 12 h).

Western blotting was performed as previously described.[44] Proteins were separated by SDS-PAGE and transferred to a polyvinylidene difluoride membrane by electroblotting. Membranes were blocked in TBS–0.1% Tween containing 3% BSA and incubated overnight at 4°C with primary antibodies. For secretion experiments, an anti-STC1 antibody was used; for phosphorylation time-course experiments (0–360 min), phospho-p38 (Cell Signaling Technology, Danvers, MA, USA; Cat. No. 4511) and total p38 antibodies (Cell Signaling Technology, Cat. No. 8690) were used; and for 12 h samples, antibodies against phospho-p53 (Cell Signaling Technology, Cat. No. 9284), total p53 (Cell Signaling Technology, Cat. No. 2524), p21 (Cell Signaling Technology, Cat. No. 2947), p27(Cell Signaling Technology, Cat. No. 3686), CDK2 (Cell Signaling Technology, Cat. No. 2546), and β-actin (Cell Signaling Technology, Cat. No. 4970) were used. Antibody information and concentrations are provided in Major Resources Table. Membranes were then incubated with peroxidase-linked secondary antibodies (1:5000; Cell Signaling Technology, Cat. Nos. 7074 and 7076) for 1 h at room temperature. Detection was performed with ECL reagent (Thermo Fisher Scientific, Cat. No. 34580), and signals were visualized using a LuminoGraph II imaging system (ATTO, Tokyo, Japan). Band intensities were quantified using ImageJ software (National Institutes of

Health).

Proliferation Assay

Proliferation of PASMCs was assessed using a BrdU colorimetric cell proliferation ELISA kit (Roche Diagnostics, Basel, Switzerland, Cat. No. 11647229001). Cells were seeded in 96-well plates at an initial density of 4×10^3 cells/well in DMEM containing 10% FBS and cultured for 48 h. After 12 h of serum starvation, the medium was replaced with either DMEM containing 5% FBS or serum-free DMEM. BrdU (10 μM) was then added, and cells were incubated for an additional 24 h. BrdU incorporation was quantified according to the manufacturer's instructions.

In parallel, cell proliferation was also evaluated by a 5-ethynyl-2′-deoxyuridine (EdU) incorporation assay. PASMCs were cultured under the indicated conditions in the presence of EdU for 24 h. Incorporated EdU was detected using a Click-iT EdU Imaging Kit (Thermo Fisher Scientific, Cat. No. C10425) according to the manufacturer's instructions. Nuclei were counterstained with DAPI, and fluorescence images were acquired under identical imaging settings.

Isolation and Culture of Mouse PASMCs

Mouse PASMCs were isolated from pulmonary arteries of WT and *Stc1*$^{-/-}$ mice exposed to chronic hypoxia (10% O_2 for 4 weeks). Briefly, distal pulmonary arteries beyond the main branches were dissected from excised lungs under a stereomicroscope. The adventitia and endothelium were carefully removed, and the medial layer was minced into small fragments. Tissue fragments were placed on culture dishes and secured with glass coverslips, and cultured in DMEM with 20% FBS and 1% penicillin/streptomycin at 37°C in a humidified atmosphere with 5% CO_2. Cells at passage 3 were used for experiments. PASMC identity was confirmed by positive staining for α-smooth muscle actin (α-SMA, data not shown). Cells were obtained from multiple independent mice and pooled for use.

Stc1 expression was confirmed by quantitative qPCR. PASMCs were treated with recombinant human STC1 (rhSTC1, 100 ng/mL) for 24 h, and cell proliferation was assessed using a BrdU incorporation assay.

Statistical Analysis

Statistical analyses were performed using GraphPad Prism (v9.2.0, GraphPad Software,

San Diego, CA, USA). Comparisons between 2 groups were conducted using the Mann–Whitney U test. For comparisons among multiple groups, the Kruskal–Wallis test was applied, followed by *post hoc* pairwise comparisons using the Mann–Whitney U test. For experiments involving repeated measurements from the same cell lot, two-group comparisons were performed using a linear mixed-effects model, whereas multiple-group comparisons were performed using a linear mixed-effects model followed by pairwise comparisons with Tukey adjustment using the *emmeans* package in R (v4.2.2). Data are expressed as mean ± SEM. *P* values are indicated in the figures.

Data Availability

RNA sequencing data have been deposited in the Gene Expression Omnibus (GEO) under accession number GSE317316 and will be made publicly available upon publication. All other data supporting the findings of this study are available from the corresponding author upon reasonable request.

Study Approval

This study was conducted in accordance with relevant ethical guidelines. For the human

study component, approval was obtained from the Institutional Review Boards of Tokyo Medical University (T2023-0084, T2026-0008) and Okayama University (1511-017), and written informed consent was obtained from all participants. For the animal experiments, approval was granted by the Institutional Animal Care and Use Committee of Tokyo Medical University (R7-058), and all procedures were performed in accordance with the principles of the 3Rs (Replacement, Reduction, and Refinement) and complied with the ARRIVE (Animal Research: Reporting of In Vivo Experiments) guidelines.

Supplemental Tables

Table S1. Summary of biological samples used in each experiment

Figure	Panel	Experiment	Sample Type	Origin	Sample IDs
1	A-D, F-G	RNA-seq	PASMCs	Human	IPAH-1-4
2	A	Immunostaining	Lung tissue	Human	Non-PAH-1, IPAH-2
2	B	Immunostaining	Lung tissue	Human	Non-PAH-1, 9-20, IPAH-2, 3, 6-14
2	C-D	STC1 western blot	PASMCs	Human	Non-PAH-2-6, IPAH-1-5
2	E	qPCR	PASMCs	Human	Non-PAH-2-6, IPAH-1-5
2	F	qPCR	PASMCs	Human	Non-PAH-6-8, IPAH-1-5
2	G	RVSP	Mouse	Mouse	-
2	H	qPCR	Lung tissue	Mouse	-
3	A-C, F, K	qPCR	PASMCs	Human	Non-PAH-6, IPAH-3
3	D-E	Immunostaining	Lung tissue	Human	Non-PAH-1, 9-20, IPAH-2, 3, 6-14
3	I-J	ChIP-PCR	PASMCs	Human	IPAH-1
4	A	Immunostaining	Lung tissue	Human	Non-PAH-1, IPAH-2
4	B	Immunostaining	Lung tissue	Human	Non-PAH-1, 9-20, IPAH-2, 3, 6-14
4	C, E, G	BrdU assay, qPCR	PASMCs	Human	Non-PAH-6
4	D, F, H	BrdU assay, qPCR	PASMCs	Human	IPAH-1-5

4	I	EdU imaging	PASMCs	Human	Non-PAH-6-8, IPAH-1, 3, 4
5	A-C	Western blot	PASMCs	Human	IPAH-3
5	D-F	BrdU assay	PASMCs	Human	IPAH-1, 3, 4
6	B-C, G-J	PH model	Mouse	Mouse	-
6	D-F	EMG staining	Lung tissue	Mouse	-
7	A-H	Immunostaining	Lung tissue	Mouse	-
8	B-D	PH model	Mouse	Mouse	-
8	E-G	EMG staining	Lung tissue	Mouse	-
8	H, I, K-M	Immunostaining	Lung tissue	Mouse	-
8	J	BrdU assay	PASMCs	Mouse	-
S4	A-B	Immunostaining	Lung tissue	Human	Non-PAH-9-20, IPAH3, 6-14
S5	A-C	qPCR	PASMCs	Human	Non-PAH-7, 8, IPAH-1, 4
S6	A-B	Immunostaining	Lung tissue	Human	Non-PAH-9-20, IPAH3, 6-14
S6	C	qPCR	PASMCs	Human	Non-PAH-7, 8, IPAH-1, 4
S7	A-G	Immunostaining	PASMCs	Human	Non-PAH-6-8, IPAH-1, 3, 4
S7	H	qPCR	PASMCs	Human	Non-PAH-7, 8, IPAH-1, 4
S8	A-B	Immunostaining	Lung tissue	Human	Non-PAH-9-20, IPAH2, 3, 6-7, 9-14
S9	A-E	BrdU assay	PASMCs	Human	Non-PAH-7, 8
S10	A	Genotyping	Tail	Mouse	-

S10	B	qPCR	Lung tissue/PASMCs	Mouse	-
S11	-	Immunostaining	Lung tissue	Mouse	-
S12	A-F	PH model	Mouse	Mouse	-
S13	-	Tunel staining	Lung tissue	Mouse	-

Table S2. Dissolved oxygen levels under hydrostatic pressure conditions

**Condition**	**Pressure Condition**	**Dissolved O_2 (mmHg)**
Atmospheric pressure		150.0
Hydrostatic pressure	70/40 mmHg, 60 bpm	156.5

Table S3. Upregulated genes in IPAH PASMCs under pressure stimulation

Gene ID	Gene Symbol	log_2 Fold Change	log_{10} (base mean + 1)
ENSG00000255282	*WTAPP1*	0.81	3.51
ENSG00000196611	*MMP1*	0.77	4.21
ENSG00000105825	*TFPI2*	0.68	3.82
ENSG00000221866	*PLXNA4*	0.65	2.14
ENSG00000159167	*STC1*	0.64	3.52
ENSG00000073756	*PTGS2*	0.59	2.79
ENSG00000137812	*KNL1*	0.56	2.69
ENSG00000153234	*NR4A2*	0.56	2.73
ENSG00000165891	*E2F7*	0.53	3.07
ENSG00000066279	*ASPM*	0.53	3.00
ENSG00000198805	*PNP*	0.51	2.65
ENSG00000112742	*TTK*	0.49	2.45
ENSG00000118193	*KIF14*	0.49	2.55
ENSG00000223701	*RAET1E-AS1*	0.48	2.19
ENSG00000138182	*KIF20B*	0.48	2.76
ENSG00000184110	*EIF3C*	0.48	2.83
ENSG00000103226	*NOMO3*	0.47	2.90
ENSG00000175832	*ETV4*	0.47	2.13
ENSG00000137309	*HMGA1*	0.47	4.25
ENSG00000138778	*CENPE*	0.47	2.87
ENSG00000139618	*BRCA2*	0.47	2.27
ENSG00000214331	*PDPR2P*	0.46	2.03
ENSG00000110031	*LPXN*	0.45	2.70
ENSG00000214262	*ANKRD36BP1*	0.45	2.30
ENSG00000169679	*BUB1*	0.45	2.69
ENSG00000156103	*MMP16*	0.45	2.59
ENSG00000135476	*ESPL1*	0.44	2.38
ENSG00000102317	*RBM3*	0.44	3.77
ENSG00000106077	*ABHD11*	0.44	2.25
ENSG00000250033	*SLC7A11-AS1*	0.44	2.61
ENSG00000210195	*MT-TT*	0.44	2.03
ENSG00000122966	*CIT*	0.44	2.92

ENSG00000117724	*CENPF*	0.43	3.13
ENSG00000185567	*AHNAK2*	0.42	3.18
ENSG00000080986	*NDC80*	0.42	2.43
ENSG00000136492	*BRIP1*	0.42	2.41
ENSG00000110427	*KIAA1549L*	0.42	2.15
ENSG00000179922	*ZNF784*	0.41	2.10
ENSG00000203930	*LINC00632*	0.41	2.50
ENSG00000127564	*PKMYT1*	0.41	2.34
ENSG00000183474	*GTF2H2C*	0.4	2.33
ENSG00000156970	*BUB1B*	0.4	2.53
ENSG00000050438	*SLC4A8*	0.4	2.88
ENSG00000024526	*DEPDC1*	0.39	2.70
ENSG00000174791	*RIN1*	0.39	2.91
ENSG00000242265	*PEG10*	0.39	3.48
ENSG00000242193	*CRYZL2P*	0.39	2.34
ENSG00000254027	*LNMICC*	0.38	2.07
ENSG00000164087	*POC1A*	0.38	2.33
ENSG00000277161	*PIGW*	0.38	2.51
ENSG00000121621	*KIF18A*	0.38	2.42
ENSG00000152256	*PDK1*	0.38	2.88
ENSG00000144959	*NCEH1*	0.37	3.37
ENSG00000103257	*SLC7A5*	0.37	3.19
ENSG00000185875	*THNSL1*	0.37	2.11
ENSG00000241313	*WWTR1-AS1*	0.37	2.58
ENSG00000168078	*PBK*	0.37	2.59
ENSG00000154217	*PITPNC1*	0.37	2.72
ENSG00000163738	*MTHFD2L*	0.37	2.06
ENSG00000184226	*PCDH9*	0.36	2.90
ENSG00000206199	*ANKUB1*	0.36	2.10
ENSG00000237596	*PDE7B-AS1*	0.36	2.04
ENSG00000197077	*KIAA1671*	0.36	2.62
ENSG00000151012	*SLC7A11*	0.36	3.56
ENSG00000185697	*MYBL1*	0.36	3.17
ENSG00000040275	*SPDL1*	0.36	2.83
ENSG00000166851	*PLK1*	0.36	2.75

ENSG00000164109	*MAD2L1*	0.36	2.64
ENSG00000196878	*LAMB3*	0.36	2.62
ENSG00000131747	*TOP2A*	0.36	3.48
ENSG00000005436	*GCFC2*	0.36	2.60
ENSG00000090889	*KIF4A*	0.35	2.66
ENSG00000137404	*NRM*	0.35	2.37
ENSG00000112297	*CRYBG1*	0.35	3.11
ENSG00000068489	*PRR11*	0.35	2.97
ENSG00000123485	*HJURP*	0.35	2.57
ENSG00000146918	*NCAPG2*	0.35	2.89
ENSG00000144136	*SLC20A1*	0.35	3.66
ENSG00000163507	*CIP2A*	0.35	2.60
ENSG00000162415	*ZSWIM5*	0.35	2.13
ENSG00000170312	*CDK1*	0.35	2.69
ENSG00000128805	*ARHGAP22*	0.35	3.15
ENSG00000011426	*ANLN*	0.35	3.40
ENSG00000258813	*RBM25-AS1*	0.35	2.22
ENSG00000102967	*DHODH*	0.35	2.05
ENSG00000075218	*GTSE1*	0.35	2.56
ENSG00000171241	*SHCBP1*	0.34	2.89
ENSG00000162063	*CCNF*	0.34	2.44
ENSG00000171522	*PTGER4*	0.34	2.18
ENSG00000122952	*ZWINT*	0.34	2.67
ENSG00000205413	*SAMD9*	0.34	3.08
ENSG00000178695	*KCTD12*	0.34	3.64
ENSG00000271643	*PDCD6IP-DT*	0.34	2.15
ENSG00000049192	*ADAMTS6*	0.34	2.21
ENSG00000147536	*GINS4*	0.34	2.59
ENSG00000126787	*DLGAP5*	0.33	2.74
ENSG00000183840	*GPR39*	0.33	2.69
ENSG00000157456	*CCNB2*	0.33	2.56
ENSG00000184661	*CDCA2*	0.33	2.45
ENSG00000225077	*ICMT-DT*	0.33	2.21
ENSG00000169607	*CKAP2L*	0.33	2.55
ENSG00000204767	*INSYN2B*	0.33	2.65

ENSG00000176055	*MBLAC2*	0.33	2.41
ENSG00000088826	*SMOX*	0.33	2.58
ENSG00000123473	*STIL*	0.33	2.46
ENSG00000138180	*CEP55*	0.33	2.74
ENSG00000148773	*MKI67*	0.33	3.20
ENSG00000072571	*HMMR*	0.33	2.56
ENSG00000175063	*UBE2C*	0.32	2.49
ENSG00000149948	*HMGA2*	0.32	3.05
ENSG00000113356	*POLR3G*	0.32	2.32
ENSG00000107614	*TRDMT1*	0.32	2.23
ENSG00000119929	*CUTC*	0.32	2.30
ENSG00000138658	*ZGRF1*	0.32	2.11
ENSG00000080603	*SRCAP*	0.32	2.07
ENSG00000184588	*PDE4B*	0.32	2.50
ENSG00000144554	*FANCD2*	0.32	2.43
ENSG00000270629	*NBPF14*	0.32	2.60
ENSG00000138376	*BARD1*	0.32	2.31
ENSG00000170961	*HAS2*	0.32	3.16
ENSG00000138160	*KIF11*	0.32	2.80
ENSG00000076662	*ICAM3*	0.32	2.28
ENSG00000168038	*ULK4*	0.32	2.04
ENSG00000237649	*KIFC1*	0.32	2.58
ENSG00000134690	*CDCA8*	0.32	2.48
ENSG00000183856	*IQGAP3*	0.32	2.79
ENSG00000128944	*KNSTRN*	0.31	2.57
ENSG00000164611	*PTTG1*	0.31	2.70
ENSG00000185900	*POMK*	0.31	2.98
ENSG00000135318	*NT5E*	0.31	4.34
ENSG00000120256	*LRP11*	0.31	2.36
ENSG00000175592	*FOSL1*	0.31	3.37
ENSG00000113368	*LMNB1*	0.31	2.72
ENSG00000178502	*KLHL11*	0.31	2.21
ENSG00000188760	*TMEM198*	0.31	2.40
ENSG00000166446	*CDYL2*	0.31	2.58
ENSG00000153044	*CENPH*	0.31	2.20

ENSG00000179041	*RRS1*	0.31	2.82
ENSG00000117155	*SSX2IP*	0.31	3.22
ENSG00000184445	*KNTC1*	0.31	2.64
ENSG00000109452	*INPP4B*	0.31	2.80
ENSG00000058804	*NDC1*	0.31	2.84
ENSG00000102312	*PORCN*	0.31	2.34
ENSG00000134602	*STK26*	0.3	2.20
ENSG00000157193	*LRP8*	0.3	2.85
ENSG00000181450	*ZNF678*	0.3	2.06
ENSG00000180875	*GREM2*	0.3	3.17
ENSG00000233452	*STXBP5-AS1*	0.3	2.23
ENSG00000245112	*SMARCA5-AS1*	0.3	2.45
ENSG00000166016	*ABTB2*	0.3	2.40
ENSG00000197020	*ZNF100*	0.3	2.04
ENSG00000154175	*ABI3BP*	0.3	3.42
ENSG00000146592	*CREB5*	0.3	2.64
ENSG00000127824	*TUBA4A*	0.29	2.67
ENSG00000259781	*HMGB1P6*	0.29	2.46
ENSG00000144152	*FBLN7*	0.29	2.26
ENSG00000076356	*PLXNA2*	0.29	2.29
ENSG00000136826	*KLF4*	0.29	2.82
ENSG00000100228	*RAB36*	0.29	2.39
ENSG00000076382	*SPAG5*	0.29	2.66
ENSG00000224424	*PRKAR2A-AS1*	0.29	2.14
ENSG00000142945	*KIF2C*	0.29	2.63
ENSG00000185480	*PARPBP*	0.29	2.16
ENSG00000105486	*LIG1*	0.29	2.56
ENSG00000149289	*ZC3H12C*	0.29	2.74
ENSG00000165434	*PGM2L1*	0.29	2.66
ENSG00000157600	*TMEM164*	0.29	2.68
ENSG00000127920	*GNG11*	0.29	3.47
ENSG00000138434	*ITPRID2*	0.29	3.95
ENSG00000162073	*PAQR4*	0.29	2.24
ENSG00000143190	*POU2F1*	0.29	2.48
ENSG00000232024	*LSM12P1*	0.29	2.29

ENSG00000198826	*ARHGAP11A*	0.28	2.77
ENSG00000213064	*SFT2D2*	0.28	3.20
ENSG00000133119	*RFC3*	0.28	2.39
ENSG00000138675	*FGF5*	0.28	3.63
ENSG00000258441	*LINC00641*	0.28	2.19
ENSG00000124126	*PREX1*	0.28	2.65
ENSG00000096433	*ITPR3*	0.28	3.35
ENSG00000176641	*RNF152*	0.28	2.86
ENSG00000183935	*HTR7P1*	0.28	2.62
ENSG00000225614	*ZNF469*	0.28	3.06
ENSG00000147799	*ARHGAP39*	0.28	2.21
ENSG00000196247	*ZNF107*	0.28	2.33
ENSG00000117399	*CDC20*	0.28	2.80
ENSG00000129317	*PUS7L*	0.28	2.95
ENSG00000174010	*KLHL15*	0.28	2.40
ENSG00000143702	*CEP170*	0.28	3.58
ENSG00000242071	*RPL7AP6*	0.28	2.63
ENSG00000134057	*CCNB1*	0.28	3.08
ENSG00000137310	*TCF19*	0.28	2.63
ENSG00000167487	*KLHL26*	0.28	2.20
ENSG00000125968	*ID1*	0.27	2.91
ENSG00000101412	*E2F1*	0.27	2.25
ENSG00000236675	*MTX1P1*	0.27	2.27
ENSG00000168404	*MLKL*	0.27	2.26
ENSG00000258725	*PRC1-AS1*	0.27	2.88
ENSG00000204634	*TBC1D8*	0.27	2.64
ENSG00000164171	*ITGA2*	0.27	3.73
ENSG00000178209	*PLEC*	0.27	4.53
ENSG00000160570	*DEDD2*	0.27	2.46
ENSG00000227028	*SLC8A1-AS1*	0.27	2.32
ENSG00000167747	*C19orf48*	0.27	2.56
ENSG00000113389	*NPR3*	0.27	3.16
ENSG00000079616	*KIF22*	0.27	2.78
ENSG00000137760	*ALKBH8*	0.27	2.42
ENSG00000100065	*CARD10*	0.27	2.81

ENSG00000166860	*ZBTB39*	0.27	2.33
ENSG00000238278	*ALG1L6P*	0.27	2.07
ENSG00000167900	*TK1*	0.27	2.99
ENSG00000178031	*ADAMTSL1*	0.27	3.22
ENSG00000167705	*RILP*	0.26	2.16
ENSG00000158467	*AHCYL2*	0.26	2.76
ENSG00000135919	*SERPINE2*	0.26	3.70
ENSG00000186310	*NAP1L3*	0.26	2.60
ENSG00000175352	*NRIP3*	0.26	2.62
ENSG00000197321	*SVIL*	0.26	3.79
ENSG00000272933	*TRIM8-DT*	0.26	2.49
ENSG00000129038	*LOXL1*	0.26	3.36
ENSG00000157510	*AFAP1L1*	0.26	2.36
ENSG00000156313	*RPGR*	0.26	2.15
ENSG00000071539	*TRIP13*	0.26	2.65
ENSG00000064225	*ST3GAL6*	0.26	2.71
ENSG00000175040	*CHST2*	0.26	2.69
ENSG00000119969	*HELLS*	0.26	2.41
ENSG00000175548	*ALG10B*	0.26	2.21
ENSG00000272031	*ANKRD34A*	0.26	2.17
ENSG00000132570	*PCBD2*	0.26	2.42
ENSG00000075213	*SEMA3A*	0.26	3.54
ENSG00000187790	*FANCM*	0.26	2.32
ENSG00000146376	*ARHGAP18*	0.26	3.26
ENSG00000114346	*ECT2*	0.26	3.15
ENSG00000169372	*CRADD*	0.26	2.26
ENSG00000168477	*TNXB*	0.25	3.71
ENSG00000180667	*YOD1*	0.25	2.47
ENSG00000163535	*SGO2*	0.25	2.67
ENSG00000224063	*CALCRL-AS1*	0.25	2.58
ENSG00000149380	*P4HA3*	0.25	2.43
ENSG00000104738	*MCM4*	0.25	3.07
ENSG00000099377	*HSD3B7*	0.25	2.70
ENSG00000073111	*MCM2*	0.25	2.68
ENSG00000162618	*ADGRL4*	0.25	2.74

ENSG00000188486	*H2AX*	0.25	2.91
ENSG00000136158	*SPRY2*	0.25	3.05
ENSG00000109805	*NCAPG*	0.25	2.79
ENSG00000080573	*COL5A3*	0.25	3.53
ENSG00000204147	*ASAH2B*	0.25	2.14
ENSG00000177034	*MTX3*	0.25	2.89
ENSG00000259125	*LRP1-AS*	0.25	2.68

Table S4. Downregulated genes in IPAH PASMCs under pressure stimulation

Gene ID	Gene Symbol	log_2 Fold Change	log_{10} (base mean + 1)
ENSG00000133392	*MYH11*	-1.00	2.92
ENSG00000180139	*ACTA2-AS1*	-0.97	3.56
ENSG00000107796	*ACTA2*	-0.93	3.87
ENSG00000151632	*AKR1C2*	-0.86	3.18
ENSG00000210151	*MT-TS1*	-0.85	2.87
ENSG00000244716	*RPL17P7*	-0.73	2.93
ENSG00000196139	*AKR1C3*	-0.66	2.87
ENSG00000220785	*MTMR9LP*	-0.64	2.46
ENSG00000240376	*RPS8P8*	-0.62	2.25
ENSG00000054277	*OPN3*	-0.60	2.86
ENSG00000212907	*MT-ND4L*	-0.59	3.83
ENSG00000103888	*CEMIP*	-0.57	4.16
ENSG00000234287	*RPS27P4*	-0.56	2.20
ENSG00000160323	*ADAMTS13*	-0.54	2.24
ENSG00000223891	*OSER1-DT*	-0.53	2.10
ENSG00000117009	*KMO*	-0.52	2.71
ENSG00000164066	*INTU*	-0.51	2.54
ENSG00000164619	*BMPER*	-0.51	2.77
ENSG00000108691	*CCL2*	-0.50	3.41
ENSG00000075826	*SEC31B*	-0.50	2.39
ENSG00000204387	*SNHG32*	-0.50	2.49
ENSG00000158286	*RNF207*	-0.50	2.34
ENSG00000188163	*FAM166A*	-0.50	2.55
ENSG00000245849	*RAD51-AS1*	-0.49	2.21
ENSG00000276168	*RN7SL1*	-0.49	2.46
ENSG00000073605	*GSDMB*	-0.49	2.18
ENSG00000276550	*HERC2P2*	-0.47	3.32
ENSG00000152527	*PLEKHH2*	-0.47	2.84
ENSG00000182310	*SPACA6*	-0.45	2.20
ENSG00000215039	*CD27-AS1*	-0.45	2.68
ENSG00000153162	*BMP6*	-0.45	2.51
ENSG00000187134	*AKR1C1*	-0.45	3.86

ENSG00000099194	*SCD*	-0.45	3.99
ENSG00000092969	*TGFB2*	-0.44	2.83
ENSG00000154721	*JAM2*	-0.44	2.51
ENSG00000100376	*FAM118A*	-0.44	2.82
ENSG00000139193	*CD27*	-0.44	2.34
ENSG00000271425	*NBPF10*	-0.44	2.08
ENSG00000186480	*INSIG1*	-0.43	3.31
ENSG00000162496	*DHRS3*	-0.43	2.50
ENSG00000132196	*HSD17B7*	-0.43	2.18
ENSG00000178338	*ZNF354B*	-0.43	2.20
ENSG00000109107	*ALDOC*	-0.43	2.54
ENSG00000272273	*IER3-AS1*	-0.43	2.66
ENSG00000126803	*HSPA2*	-0.41	2.76
ENSG00000072195	*SPEG*	-0.41	3.55
ENSG00000120832	*MTERF2*	-0.41	2.23
ENSG00000106624	*AEBP1*	-0.41	3.35
ENSG00000244165	*P2RY11*	-0.41	2.05
ENSG00000087076	*HSD17B14*	-0.41	2.04
ENSG00000257809	*MYL6B-AS1*	-0.40	2.16
ENSG00000144029	*MRPS5*	-0.40	2.16
ENSG00000108465	*CDK5RAP3*	-0.40	2.30
ENSG00000049769	*PPP1R3F*	-0.40	2.30
ENSG00000139239	*RPL14P1*	-0.39	2.22
ENSG00000172403	*SYNPO2*	-0.39	3.93
ENSG00000104081	*BMF*	-0.39	2.35
ENSG00000154553	*PDLIM3*	-0.39	2.37
ENSG00000233762	*RPS15P4*	-0.38	2.31
ENSG00000147883	*CDKN2B*	-0.38	2.77
ENSG00000112139	*MDGA1*	-0.38	3.38
ENSG00000142102	*PGGHG*	-0.38	2.39
ENSG00000169228	*RAB24*	-0.38	2.04
ENSG00000090339	*ICAM1*	-0.38	2.36
ENSG00000213983	*AP1G2*	-0.38	2.86
ENSG00000213626	*LBH*	-0.38	4.07
ENSG00000250182	*EEF1A1P13*	-0.37	2.04

ENSG00000099957	*P2RX6*	-0.37	2.15
ENSG00000117616	*RSRP1*	-0.37	3.24
ENSG00000144134	*RABL2A*	-0.37	2.27
ENSG00000213741	*RPS29*	-0.37	3.39
ENSG00000183648	*NDUFB1*	-0.37	2.65
ENSG00000254860	*TMEM9B-AS1*	-0.37	2.01
ENSG00000264538	*SUZ12P1*	-0.36	2.03
ENSG00000258727	*AP1G2-AS1*	-0.36	2.64
ENSG00000168575	*SLC20A2*	-0.36	3.36
ENSG00000113240	*CLK4*	-0.36	2.80
ENSG00000167693	*NXN*	-0.36	3.35
ENSG00000240771	*ARHGEF25*	-0.36	2.68
ENSG00000004534	*RBM6*	-0.35	3.30
ENSG00000121310	*ECHDC2*	-0.35	3.00
ENSG00000113578	*FGF1*	-0.35	2.73
ENSG00000226328	*NUP50-DT*	-0.35	2.01
ENSG00000237973	*MTCO1P12*	-0.35	2.89
ENSG00000158201	*ABHD3*	-0.35	2.12
ENSG00000198840	*MT-ND3*	-0.35	4.07
ENSG00000084693	*AGBL5*	-0.35	3.18
ENSG00000242259	*C22orf39*	-0.34	2.40
ENSG00000198743	*SLC5A3*	-0.34	3.28
ENSG00000132635	*PCED1A*	-0.34	2.77
ENSG00000203709	*MIR29B2CHG*	-0.34	2.37
ENSG00000103485	*QPRT*	-0.34	2.81
ENSG00000234771	*SLC25A25-AS1*	-0.34	2.12
ENSG00000111364	*DDX55*	-0.34	2.63
ENSG00000149591	*TAGLN*	-0.34	4.56
ENSG00000175265	*GOLGA8A*	-0.34	2.77
ENSG00000171812	*COL8A2*	-0.34	2.33
ENSG00000071282	*LMCD1*	-0.34	2.73
ENSG00000214021	*TTLL3*	-0.33	2.89
ENSG00000196739	*COL27A1*	-0.33	2.88
ENSG00000131067	*GGT7*	-0.33	2.89
ENSG00000259431	*THTPA*	-0.33	2.11

ENSG00000132541	*RIDA*	-0.33	2.98
ENSG00000117525	*F3*	-0.33	3.91
ENSG00000204257	*HLA-DMA*	-0.33	2.50
ENSG00000279457	*WASH9P*	-0.33	2.23
ENSG00000011638	*LDAF1*	-0.33	2.98
ENSG00000213178	*RPL22P1*	-0.33	2.40
ENSG00000104892	*KLC3*	-0.33	2.35
ENSG00000196268	*ZNF493*	-0.32	2.05
ENSG00000257621	*PSMA3-AS1*	-0.32	2.75
ENSG00000154917	*RAB6B*	-0.32	2.08
ENSG00000119699	*TGFB3*	-0.32	2.22
ENSG00000149451	*ADAM33*	-0.32	3.17
ENSG00000225855	*RUSC1-AS1*	-0.32	3.25
ENSG00000136048	*DRAM1*	-0.32	3.49
ENSG00000204253	*HNRNPCP2*	-0.32	2.35
ENSG00000116157	*GPX7*	-0.32	2.35
ENSG00000269293	*ZSCAN16-AS1*	-0.32	2.09
ENSG00000156976	*EIF4A2*	-0.32	3.95
ENSG00000222009	*BTBD19*	-0.32	2.70
ENSG00000090238	*YPEL3*	-0.32	2.70
ENSG00000198046	*ZNF667*	-0.32	2.22
ENSG00000153246	*PLA2R1*	-0.32	2.66
ENSG00000035115	*SH3YL1*	-0.32	2.84
ENSG00000122085	*MTERF4*	-0.32	3.48
ENSG00000168781	*PPIP5K1*	-0.32	2.37
ENSG00000131069	*ACSS2*	-0.32	3.00
ENSG00000119280	*C1orf198*	-0.31	3.50
ENSG00000105855	*ITGB8*	-0.31	2.83
ENSG00000167508	*MVD*	-0.31	3.05
ENSG00000186567	*CEACAM19*	-0.31	2.51
ENSG00000162600	*OMA1*	-0.31	2.32
ENSG00000153048	*CARHSP1*	-0.31	3.01
ENSG00000166347	*CYB5A*	-0.31	2.93
ENSG00000178096	*BOLA1*	-0.31	2.03
ENSG00000163482	*STK36*	-0.31	2.84

ENSG00000100836	*PABPN1*	-0.31	2.62
ENSG00000013441	*CLK1*	-0.31	3.31
ENSG00000178498	*DTX3*	-0.31	2.82
ENSG00000242299	*RPS18P5*	-0.31	2.67
ENSG00000144115	*THNSL2*	-0.31	2.60
ENSG00000160752	*FDPS*	-0.30	3.41
ENSG00000139190	*VAMP1*	-0.30	2.75
ENSG00000186166	*CENATAC*	-0.30	2.41
ENSG00000137817	*PARP6*	-0.30	2.77
ENSG00000137193	*PIM1*	-0.30	2.61
ENSG00000160766	*GBAP1*	-0.30	2.21
ENSG00000163155	*LYSMD1*	-0.30	2.15
ENSG00000245532	*NEAT1*	-0.30	4.07
ENSG00000174348	*PODN*	-0.30	3.03
ENSG00000148120	*AOPEP*	-0.30	2.88
ENSG00000146574	*CCZ1B*	-0.30	2.66
ENSG00000160613	*PCSK7*	-0.30	4.24
ENSG00000134824	*FADS2*	-0.30	3.74
ENSG00000175274	*TP53I11*	-0.30	3.84
ENSG00000052802	*MSMO1*	-0.30	3.36
ENSG00000170989	*S1PR1*	-0.30	2.50
ENSG00000112972	*HMGCS1*	-0.30	3.14
ENSG00000163516	*ANKZF1*	-0.30	2.66
ENSG00000247828	*TMEM161B-DT*	-0.30	2.49
ENSG00000167863	*ATP5PD*	-0.30	3.43
ENSG00000135299	*ANKRD6*	-0.30	2.15
ENSG00000110455	*ACCS*	-0.30	2.85
ENSG00000134243	*SORT1*	-0.30	3.40
ENSG00000164932	*CTHRC1*	-0.30	2.82
ENSG00000107968	*MAP3K8*	-0.30	2.39
ENSG00000113916	*BCL6*	-0.30	2.99
ENSG00000140450	*ARRDC4*	-0.30	2.96
ENSG00000198727	*MT-CYB*	-0.30	4.72
ENSG00000214548	*MEG3*	-0.30	3.87
ENSG00000180573	*H2AC6*	-0.30	3.14

ENSG00000159403	*C1R*	-0.30	3.88
ENSG00000223799	*IL10RB-DT*	-0.30	2.02
ENSG00000164251	*F2RL1*	-0.29	2.45
ENSG00000214174	*AMZ2P1*	-0.29	2.29
ENSG00000196730	*DAPK1*	-0.29	2.75
ENSG00000109944	*JHY*	-0.29	2.13
ENSG00000134253	*TRIM45*	-0.29	2.09
ENSG00000263072	*ZNF213-AS1*	-0.29	2.43
ENSG00000154803	*FLCN*	-0.29	2.99
ENSG00000106992	*AK1*	-0.29	2.71
ENSG00000134107	*BHLHE40*	-0.29	2.71
ENSG00000214530	*STARD10*	-0.29	2.34
ENSG00000153904	*DDAH1*	-0.29	3.69
ENSG00000129116	*PALLD*	-0.29	4.16
ENSG00000168906	*MAT2A*	-0.29	4.04
ENSG00000139182	*CLSTN3*	-0.29	2.86
ENSG00000099949	*LZTR1*	-0.29	2.64
ENSG00000114988	*LMAN2L*	-0.29	2.84
ENSG00000161551	*ZNF577*	-0.29	2.26
ENSG00000162804	*SNED1*	-0.29	3.94
ENSG00000162994	*CLHC1*	-0.29	2.22
ENSG00000137098	*SPAG8*	-0.29	2.23
ENSG00000013583	*HEBP1*	-0.29	3.32
ENSG00000160469	*BRSK1*	-0.29	2.45
ENSG00000235437	*LINC01278*	-0.28	2.58
ENSG00000135315	*CEP162*	-0.28	2.17
ENSG00000219507	*FTH1P8*	-0.28	2.44
ENSG00000237714	*P4HA2-AS1*	-0.28	2.36
ENSG00000204344	*STK19*	-0.28	2.62
ENSG00000239523	*MYLK-AS1*	-0.28	3.30
ENSG00000101335	*MYL9*	-0.28	4.37
ENSG00000067445	*TRO*	-0.28	2.87
ENSG00000175198	*PCCA*	-0.28	2.55
ENSG00000111653	*ING4*	-0.28	2.65
ENSG00000176593	*ZNF606-AS1*	-0.28	2.44

ENSG00000226696	*LENG8-AS1*	-0.28	2.42
ENSG00000161281	*COX7A1*	-0.28	2.87
ENSG00000140416	*TPM1*	-0.28	4.03
ENSG00000127831	*VIL1*	-0.28	2.01
ENSG00000173918	*C1QTNF1*	-0.28	2.89
ENSG00000149485	*FADS1*	-0.28	3.63
ENSG00000105655	*ISYNA1*	-0.28	2.36
ENSG00000175183	*CSRP2*	-0.28	2.51
ENSG00000136932	*TRMO*	-0.28	2.22
ENSG00000114735	*HEMK1*	-0.28	2.80
ENSG00000139112	*GABARAPL1*	-0.28	3.52
ENSG00000231064	*THBS3-AS1*	-0.28	2.96
ENSG00000267040	*ATP8B1-AS1*	-0.28	2.56
ENSG00000048052	*HDAC9*	-0.28	2.75
ENSG00000119878	*CRIPT*	-0.28	2.71
ENSG00000198695	*MT-ND6*	-0.27	4.37
ENSG00000064687	*ABCA7*	-0.27	2.56
ENSG00000122862	*SRGN*	-0.27	3.43
ENSG00000239900	*ADSL*	-0.27	2.22
ENSG00000243970	*PPIEL*	-0.27	2.32
ENSG00000148926	*ADM*	-0.27	3.56
ENSG00000182586	*LINC00334*	-0.27	2.60
ENSG00000198467	*TPM2*	-0.27	4.49
ENSG00000137331	*IER3*	-0.27	3.05
ENSG00000042429	*MED17*	-0.27	2.04
ENSG00000135931	*ARMC9*	-0.27	3.50
ENSG00000169231	*THBS3*	-0.27	3.16
ENSG00000107372	*ZFAND5*	-0.27	3.81
ENSG00000165507	*DEPP1*	-0.27	2.72
ENSG00000143772	*ITPKB*	-0.27	2.71
ENSG00000187049	*TMEM216*	-0.27	2.04
ENSG00000256453	*DND1*	-0.27	2.24
ENSG00000204381	*LAYN*	-0.27	3.25
ENSG00000204681	*GABBR1*	-0.27	2.91
ENSG00000232656	*IDI2-AS1*	-0.26	2.43

ENSG00000241399	*CD302*	-0.26	2.82
ENSG00000232573	*RPL3P4*	-0.26	2.97
ENSG00000165996	*HACD1*	-0.26	2.73
ENSG00000184162	*NR2C2AP*	-0.26	2.30
ENSG00000223959	*AFG3L1P*	-0.26	2.41
ENSG00000281691	*RBM5-AS1*	-0.26	2.61
ENSG00000239779	*WBP1*	-0.26	2.21
ENSG00000153774	*CFDP1*	-0.26	2.78
ENSG00000132000	*PODNL1*	-0.26	2.34
ENSG00000117899	*MESD*	-0.26	3.23
ENSG00000142871	*CCN1*	-0.26	4.02
ENSG00000228474	*OST4*	-0.26	3.45
ENSG00000225377	*NRSN2-AS1*	-0.26	2.13
ENSG00000164291	*ARSK*	-0.26	2.95
ENSG00000228526	*MIR34AHG*	-0.26	2.53
ENSG00000072864	*NDE1*	-0.26	2.66
ENSG00000173706	*HEG1*	-0.26	4.10
ENSG00000176809	*LRRC37A3*	-0.26	2.13
ENSG00000139631	*CSAD*	-0.26	2.77
ENSG00000221978	*CCNL2*	-0.26	3.58
ENSG00000110446	*SLC15A3*	-0.26	2.47
ENSG00000196476	*C20orf96*	-0.26	2.18
ENSG00000197283	*SYNGAP1*	-0.26	2.95
ENSG00000142920	*AZIN2*	-0.26	2.10
ENSG00000234420	*ZNF37BP*	-0.26	2.72
ENSG00000172172	*MRPL13*	-0.26	2.73
ENSG00000170310	*STX8*	-0.26	2.54
ENSG00000107551	*RASSF4*	-0.25	2.51
ENSG00000275183	*LENG9*	-0.25	2.46
ENSG00000108848	*LUC7L3*	-0.25	3.54
ENSG00000156804	*FBXO32*	-0.25	2.80
ENSG00000167733	*HSD11B1L*	-0.25	2.05
ENSG00000161647	*MPP3*	-0.25	2.14
ENSG00000122707	*RECK*	-0.25	3.71
ENSG00000026652	*AGPAT4*	-0.25	2.52

ENSG00000172893	*DHCR7*	-0.25	3.17
ENSG00000163795	*ZNF513*	-0.25	2.67
ENSG00000100342	*APOL1*	-0.25	3.26
ENSG00000198625	*MDM4*	-0.25	2.72
ENSG00000130584	*ZBTB46*	-0.25	2.07
ENSG00000130164	*LDLR*	-0.25	3.85
ENSG00000133624	*ZNF767P*	-0.25	2.39
ENSG00000132801	*ZSWIM3*	-0.25	2.02
ENSG00000132793	*LPIN3*	-0.25	2.59
ENSG00000220323	*H2BC19P*	-0.25	2.18
ENSG00000143624	*INTS3*	-0.25	3.36
ENSG00000204227	*RING1*	-0.25	3.11
ENSG00000067836	*ROGDI*	-0.25	2.16

Table S5. Characteristics of mice exposed to normoxia or hypoxia

**Group**	**Genotype**	**Sex**	**Exposure**	**Age (mo)**	**Body Weight (g)**	**Tibia Length (mm)**	***n***
Normoxia	WT	Male	Normoxia	3.8 ± 0.1	27.2 ± 2.4	20.8 ± 1.0	5
Normoxia	WT	Female	Normoxia	3.8 ± 0.1	18.8 ± 0.8	18.6 ± 0.4	5
Normoxia	$Stc1^{-/-}$	Male	Normoxia	3.6 ± 0.3	29.4 ± 1.5	20.0 ± 0.8	5
Normoxia	$Stc1^{-/-}$	Female	Normoxia	3.9 ± 0.1	22.4 ± 3.2	18.8 ± 0.3	5
Hypoxia	WT	Male	Hypoxia	3.5 ± 0.2	28.5 ± 2.5	20.0 ± 1.2	5
Hypoxia	WT	Female	Hypoxia	3.6 ± 0.2	19.8 ± 1.1	18.7 ± 0.3	5
Hypoxia	$Stc1^{-/-}$	Male	Hypoxia	3.7 ± 0.3	25.7 ± 2.5	19.7 ± 0.5	5
Hypoxia	$Stc1^{-/-}$	Female	Hypoxia	3.9 ± 0.1	20.0 ± 1.6	19.2 ± 0.3	5

Table S6. Two-way ANOVA analysis assessing sex-specific effects on experimental parameters

Hypoxia

Parameter	Male		Female	
	WT	*Stc1*$^{-/-}$	WT	*Stc1*$^{-/-}$
RVSP (mmHg)	26.7 ± 2.1	39.6 ± 5.9	28.8 ± 1.6	45.1 ± 4.8
Medial wall thickness (%)	26.6 ± 3.7	37.7 ± 7.0	26.4 ± 5.0	40.6 ± 9.2

Two-way ANOVA Parameter	Effect	F (df1, df2)	*P*
RVSP	Genotype	F (1, 16) = 66.16	<0.0001
	Sex	F (1, 16) = 4.460	0.0508
	Interaction	F (1, 16) = 0.9139	0.3533
Medial thickness	Genotype	F (1, 16) = 18.44	0.0006
	Sex	F (1, 16) = 0.2169	0.6477
	Interaction	F (1, 16) = 0.2566	0.6194

WT

Parameter	Male		Female	
	Normoxia	Hypoxia	Normoxia	Hypoxia
RVSP (mmHg)	19.22 ± 1.8011	26.67 ± 2.1	19.7 ± 3.7	28.8 ± 1.6
Medial wall thickness (%)	17.96 ± 2.0	26.56 ± 3.7	15.88 ± 5.1	26.44 ± 5.0
Fulton index	0.212 ± 0.0075	0.314 ± 0.015	0.216 ± 0.0014	0.303 ± 0.021

Two-way ANOVA

Parameter	Effect	F (df1, df2)	*P*

RVSP	Oxygen	F (1, 16) = 57.68	<0.0001
	Sex	F (1, 16) = 1.397	0.254
	Interaction	F (1, 16) = 0.5420	0.472
Medial thickness	Oxygen	F (1, 16) = 26.57	<0.0001
	Sex	F (1, 16) = 0.3503	0.562
	Interaction	F (1, 16) = 0.2780	0.605
Fulton index	Oxygen	F (1, 16) = 217.0	<0.0001
	Sex	F (1, 16) = 0.9231	0.351
	Interaction	F (1, 16) = 2.564	0.129

Stc1 $^{-/-}$

Parameter	Male		Female	
	Normoxia	Hypoxia	Normoxia	Hypoxia
RVSP (mmHg)	18.96 ± 2.5897	39.6 ± 5.9	19.5 ± 2.1	45.1 ± 4.8
Medial wall thickness (%)	16.52 ± 2.2	37.7 ± 7.0	16.2 ± 2.9	40.56 ± 9.2
Fulton index	0.227 ± 0.0261	0.299 ± 0.020	0.216 ± 0.0167	0.314 ± 0.035

Two-way ANOVA

Parameter	Effect	F (df1, df2)	*P*
RVSP	Oxygen	F (1, 16) = 154.9	<0.0001
	Sex	F (1, 16) = 2.696	0.120
	Interaction	F (1, 16) = 1.777	0.201
Medial thickness	Oxygen	F (1, 16) = 70.45	<0.0001
	Sex	F (1, 16) = 0.2192	0.646
	Interaction	F (1, 16) = 0.3435	0.566
Fulton index	Oxygen	F (1, 16) = 56.24	<0.0001
	Sex	F (1, 16) = 0.000	>0.9999
	Interaction	F (1, 16) = 1.490	0.240

Supplemental Figures and Legends

Figure S1

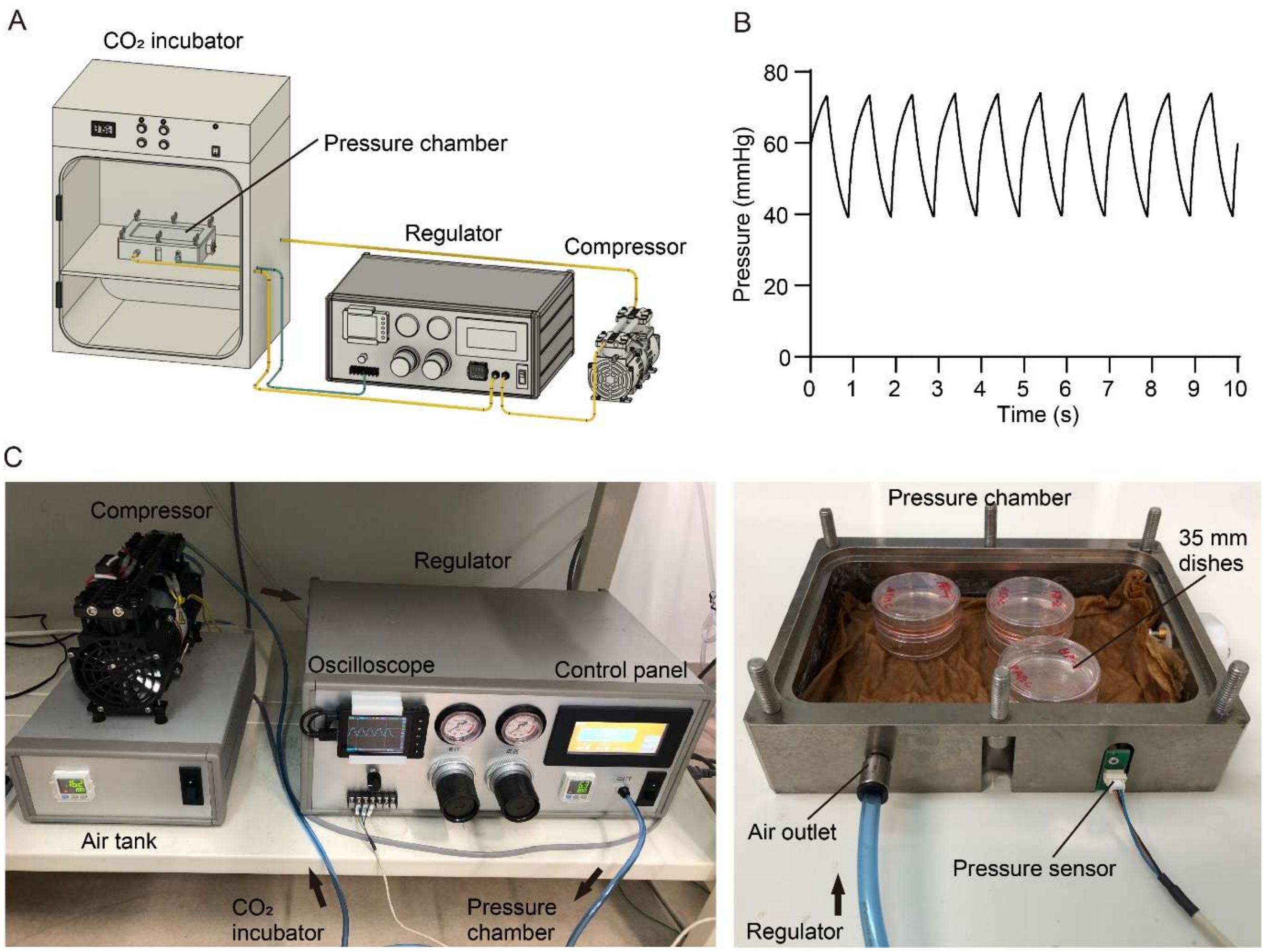

Figure S1. Overview of the cyclic hydrostatic pressure system.

A, Schematic diagram of the cyclic hydrostatic pressure apparatus. **B**, Representative waveform of cyclic hydrostatic pressure. **C,** Photographs of the actual apparatus. The left panel shows the regulator and compressor, and the right panel shows the pressure chamber. Arrows indicate the direction of airflow.

Figure S2

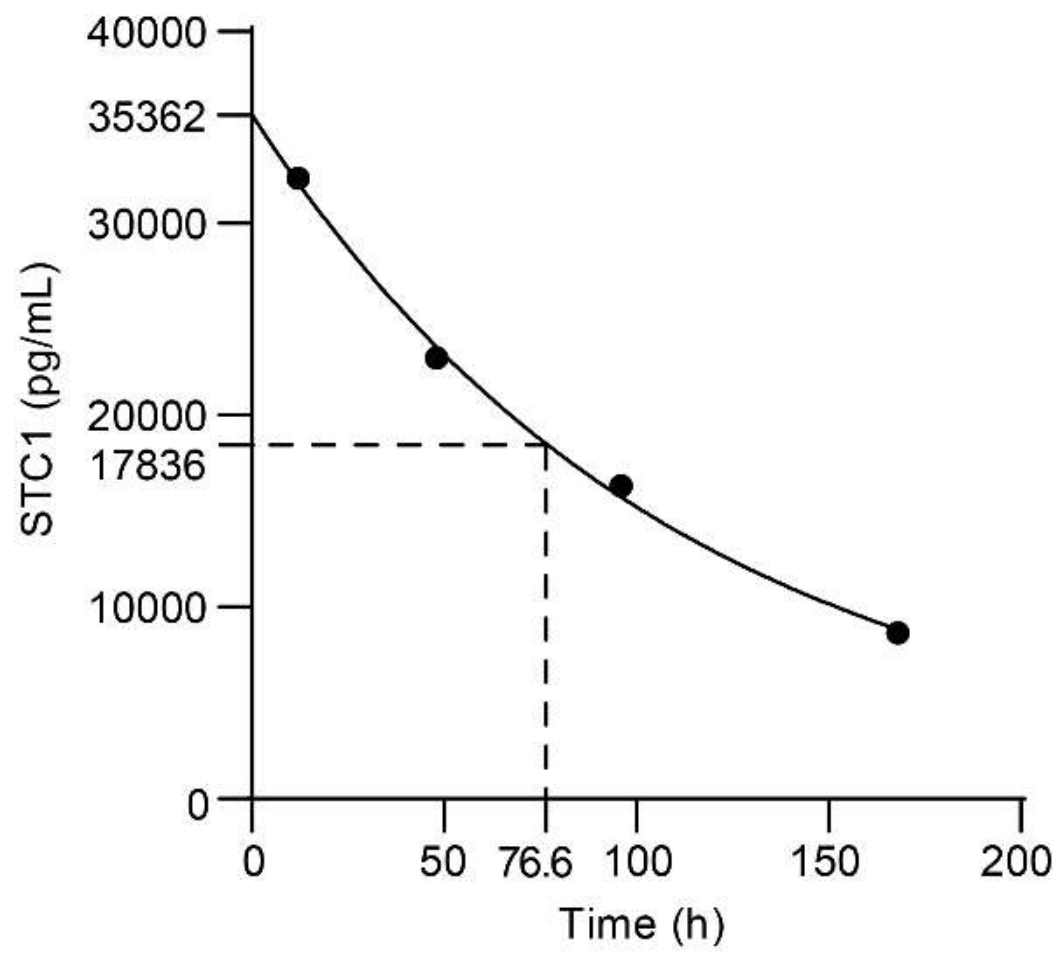

Figure S2. Pulmonary stanniocalcin-1 (STC1) concentration after intratracheal administration of recombinant human STC1 (rhSTC1).

Stc1$^{-/-}$ mice received a single intratracheal dose of rhSTC1 (2 μg in 50 μL phosphate-buffered saline [PBS]). At 12, 48, 96, and 168 hours after administration, whole lungs were collected and homogenized in 2.5 mL of carbonate buffer. Pulmonary STC1 levels were quantified by ELISA, and the half-life was calculated from the regression curve.

Figure S3

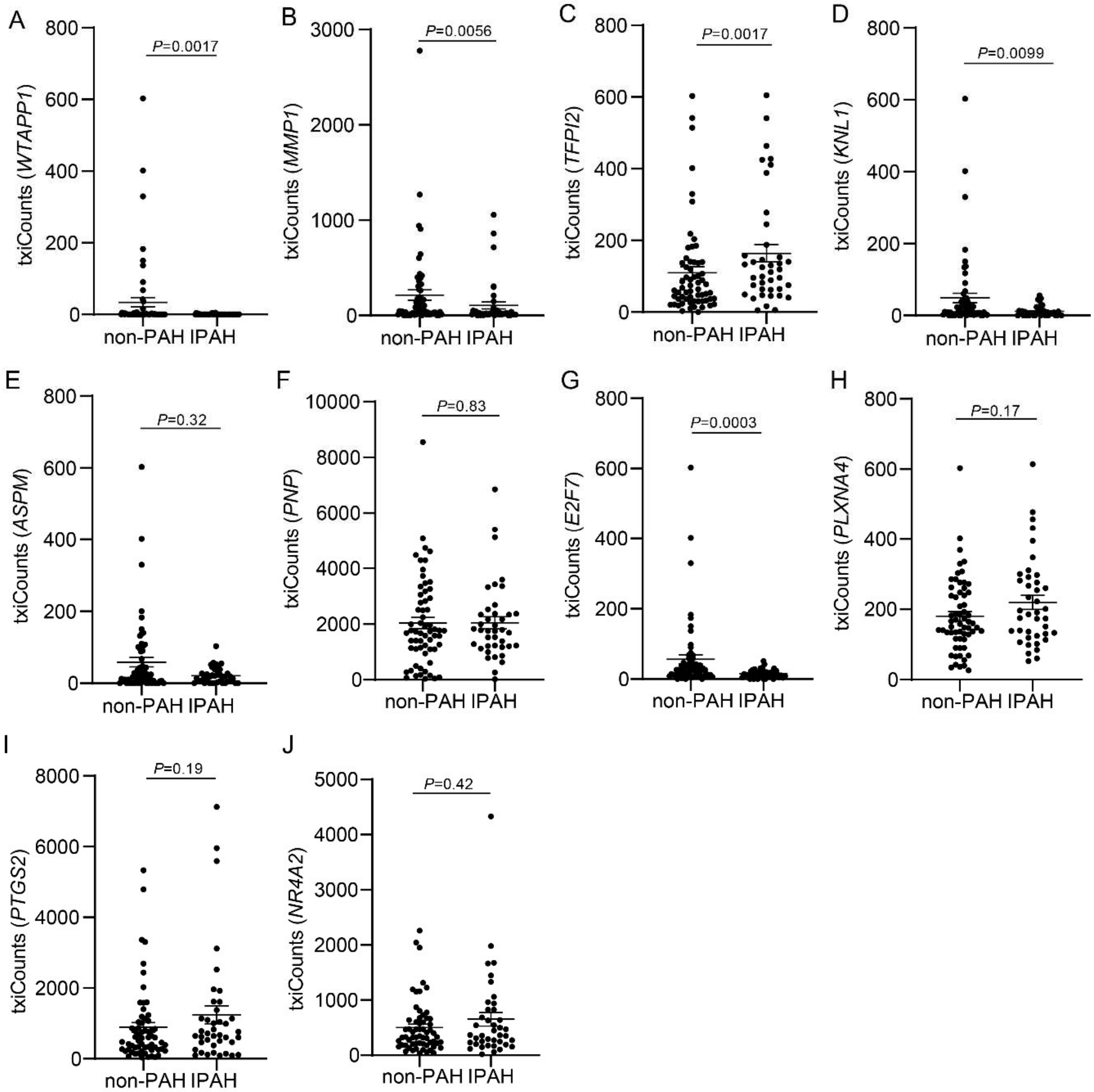

Figure S3. Gene expression in human lung RNA-seq datasets. A-J, Transcript-level counts (txiCounts) of genes upregulated by hydrostatic pressure (log_2 fold change >0.5 in the MA plot in Figure 1E) were extracted from a previously published RNA-seq dataset comparing non-PAH lungs (n=40) and IPAH lungs (n=52). Data are presented as

individual samples with mean ± SEM. Statistical significance was determined using the Mann–Whitney U test, and *P* values are indicated.

Figure S4

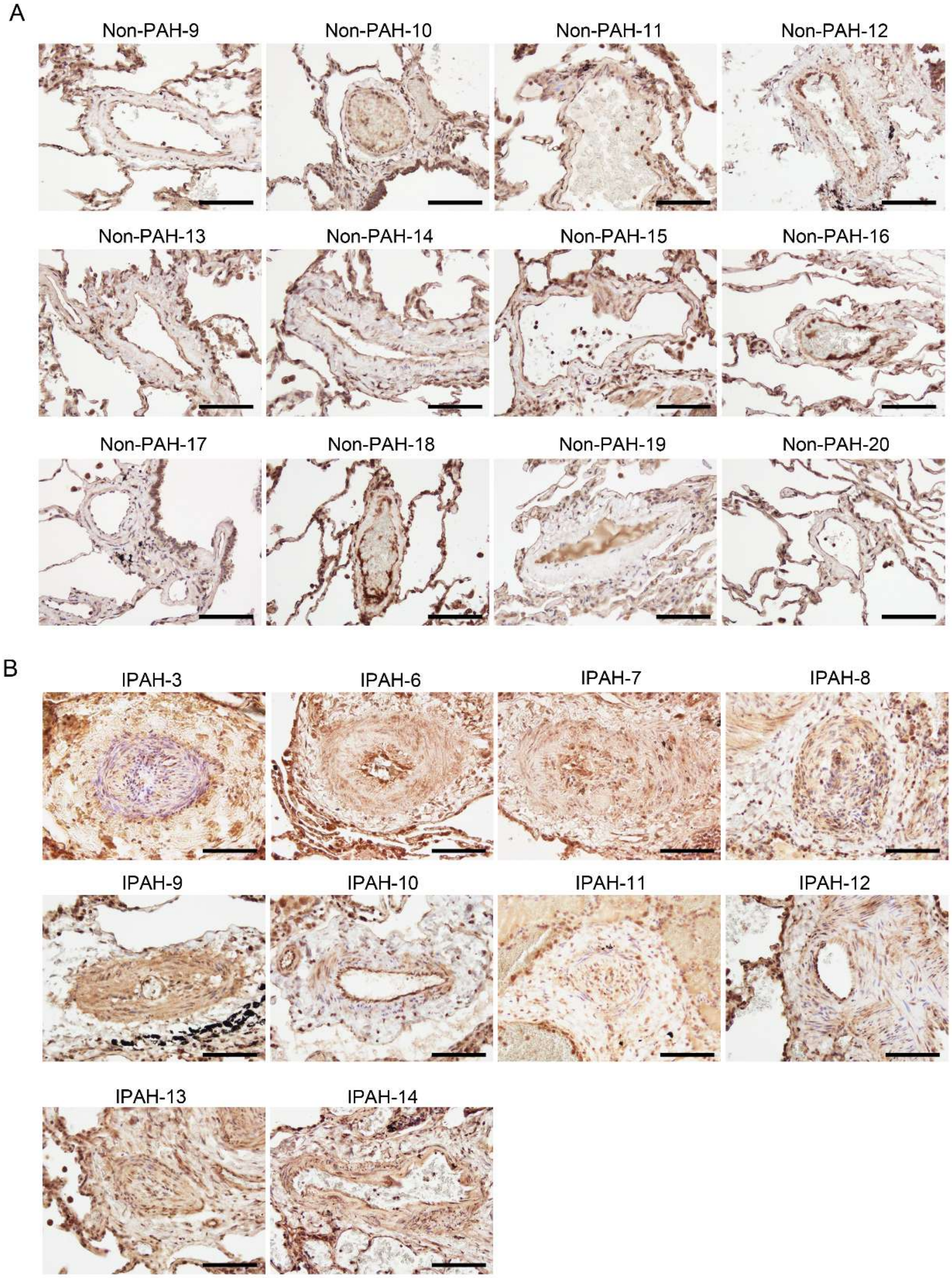

Figure S4. STC1 expression in lung tissues from non-PAH and IPAH patients.

A, Representative immunohistochemistry (IHC) images of STC1 in lung tissues from

individual non-PAH patients (n=12). **B**, Representative IHC images of STC1 in lung tissues from individual IPAH patients (n=10). Images are shown for each patient. Scale bars, 100 μm.

Figure S5

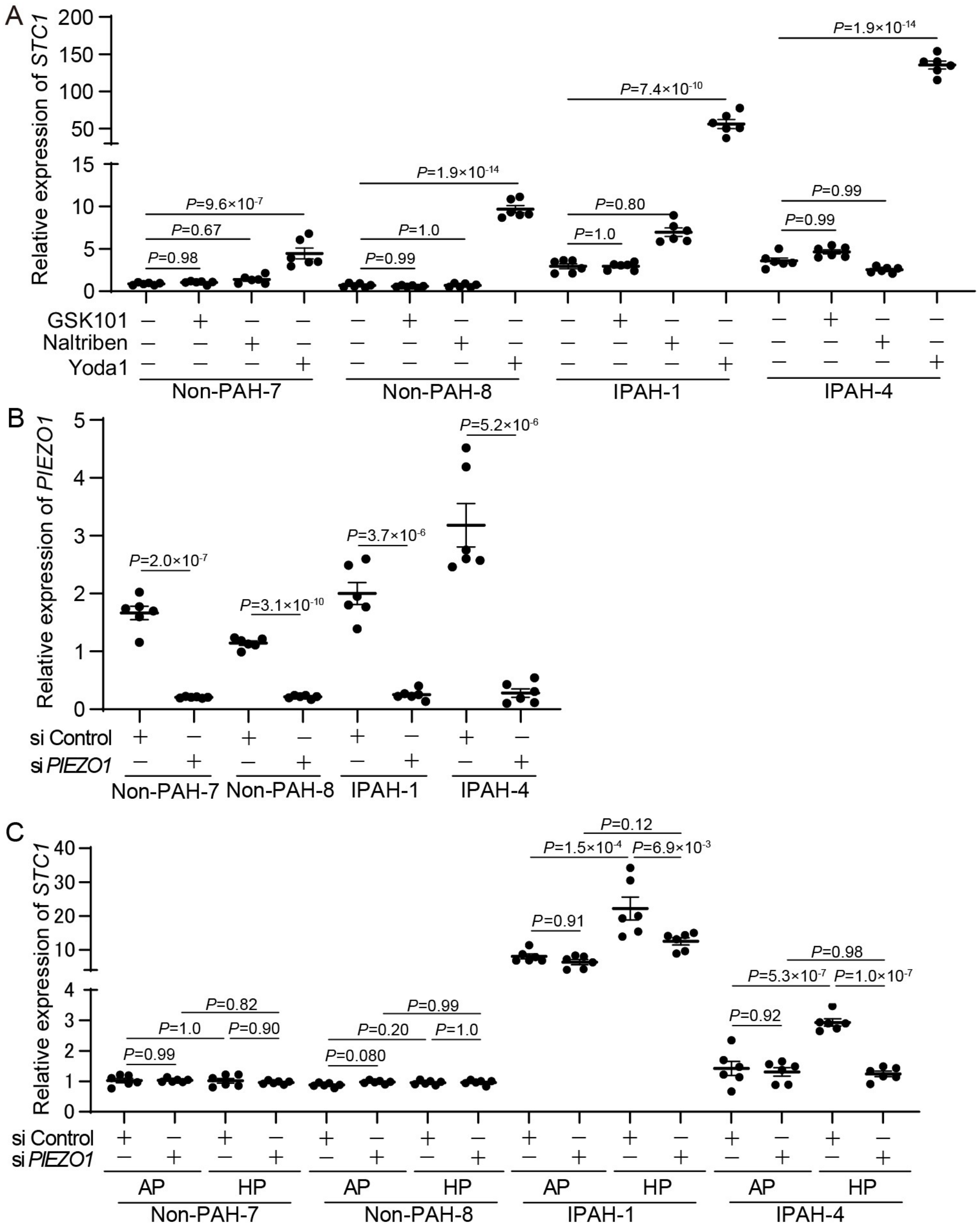

Figure S5. Mechanotransduction-induced *STC1* expression in PASMCs from independent patients.

Results obtained using PASMCs derived from independent patients not used in Figure 3.

A, Effects of mechanosensitive receptor activation on *STC1* expression. Relative

expression of *STC1* mRNA following stimulation of mechanosensitive receptors in pulmonary artery smooth muscle cells (PASMCs) from non-PAH (Non-PAH-7 and -8) and IPAH patients (IPAH-1 and -4). Cells were stimulated with GSK1016790A (GSK101, 10 nmol/L), an agonist of transient receptor potential vanilloid 4 (TRPV4); naltriben (20 μmol/L), an agonist of transient receptor potential melastatin 7 (TRPM7); or Yoda1 (20 μmol/L), an agonist of PIEZO1. *STC1* mRNA expression was quantified by quantitative polymerase chain reaction (qPCR). **B**, Efficiency of *PIEZO1* knockdown. PASMCs from non-PAH and IPAH patients (Non-PAH-7 and -8, IPAH-1 and -4) were transfected with small interfering RNA targeting PIEZO1 (si *PIEZO1*) or control siRNA (si Control), and knockdown efficiency was assessed by qPCR. **C**, Effect of *PIEZO1* knockdown on *STC1* expression under cyclic hydrostatic pressure. PASMCs from non-PAH (Non-PAH-7 and -8) and IPAH patients (IPAH-1 and -4) were exposed to cyclic hydrostatic pressure (70/40 mmHg, 60 bpm) for 72 h with or without *PIEZO1* knockdown. Relative expression of *STC1* mRNA was measured by qPCR. AP, atmospheric pressure; HP, hydrostatic pressure. Data are presented as mean ± SEM; n=6 independent experiments. Comparisons between two groups and among multiple groups were performed using linear mixed-effects models with post hoc pairwise

comparisons using the *emmeans* with Tukey adjustment. *P* values are indicated

Figure S6

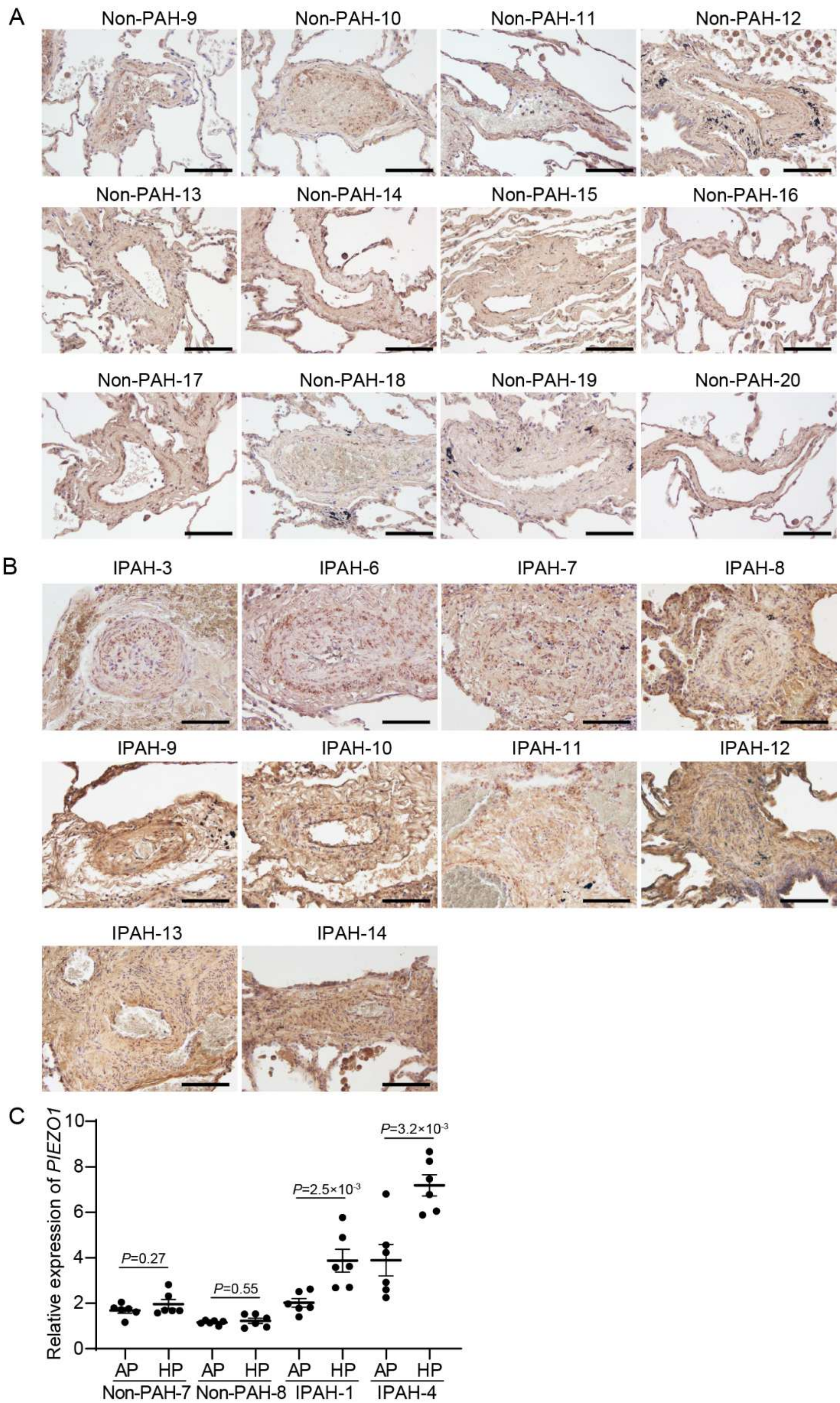
A
Non-PAH-9
Non-PAH-10
Non-PAH-11
Non-PAH-12
Non-PAH-13
Non-PAH-14
Non-PAH-15
Non-PAH-16
Non-PAH-17
Non-PAH-18
Non-PAH-19
Non-PAH-20
B
IPAH-3
IPAH-6
IPAH-7
IPAH-8
IPAH-9
IPAH-10
IPAH-11
IPAH-12
IPAH-13
IPAH-14
C
Relative expression of PIEZO1
10
8
6
4
2
0
P=0.27
P=0.55
P=2.5×10-3
P=3.2×10-3
AP HP AP HP AP HP AP HP
Non-PAH-7 Non-PAH-8 IPAH-1 IPAH-4

Figure S6. PIEZO1 expression in lung tissues and its regulation by hydrostatic pressure.

A, B, Representative immunohistochemical (IHC) images of PIEZO1 in lung tissues from non-PAH (**A**; n=12) and IPAH patients (**B**; n=10). Scale bars, 100 μm. **C**, Changes in *PIEZO1* expression in PASMCs following exposure to cyclic hydrostatic pressure. Pulmonary artery smooth muscle cells (PASMCs) from non-PAH (Non-PAH-7 and -8) and IPAH patients (IPAH-1 and -4) were exposed to cyclic hydrostatic pressure for 72 h. Relative expression of *PIEZO1* was assessed by quantitative polymerase chain reaction (qPCR). Data are presented as mean ± SEM (n=6). Comparisons between two groups were performed using linear mixed-effects models with post hoc pairwise comparisons using the *emmeans* with Tukey adjustment. *P* values are indicated.

Figure S7

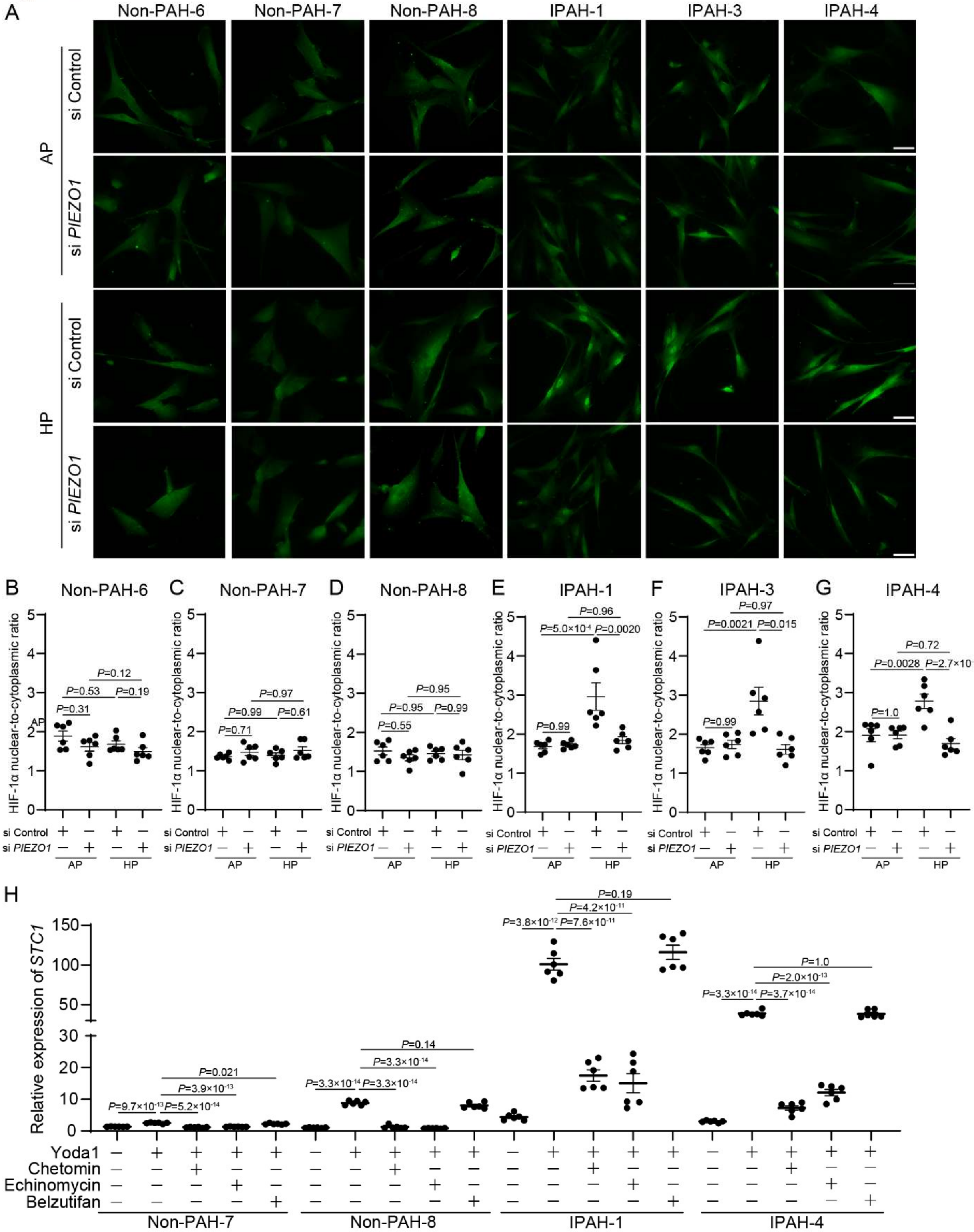

Figure S7. HIF-1α nuclear translocation and STC1 regulation downstream of

PIEZO1.

A, Representative immunocytochemistry (ICC) images of HIF-1α localization in PASMCs from non-PAH (Non-PAH-6, -7, and -8) and IPAH patients (IPAH-1, -3, and -4), with or without cyclic hydrostatic pressure (70/40 mmHg, 60 bpm) and *PIEZO1* knockdown. Scale bars, 50 μm. **B–G**, Quantification of HIF-1α nuclear-to-cytoplasmic ratio in PASMCs from non-PAH (**B**–**D**; Non-PAH-6, -7, and -8) and IPAH patients (**E**–**G**; IPAH-1, -3, and -4). **H**, Effect of hypoxia-inducible factor (HIF) inhibition on PIEZO1-mediated STC1 upregulation. PASMCs from non-PAH (Non-PAH-7 and -8) and IPAH patients (IPAH-1 and -4) were treated with HIF inhibitors during PIEZO1 activation. HIF-1α was inhibited using chetomin (100 nmol/L) or echinomycin (30 nmol/L), and HIF-2α was inhibited using belzutifan (10 nmol/L). PIEZO1 was activated using Yoda1. Relative expression of *STC1* mRNA was quantified by quantitative polymerase chain reaction (qPCR). Data are presented as mean ± SEM (n=6). Comparisons among multiple groups were performed using linear mixed-effects models, and post hoc pairwise comparisons were conducted using the *emmeans* with Tukey adjustment. *P* values are indicated.

Figure S8

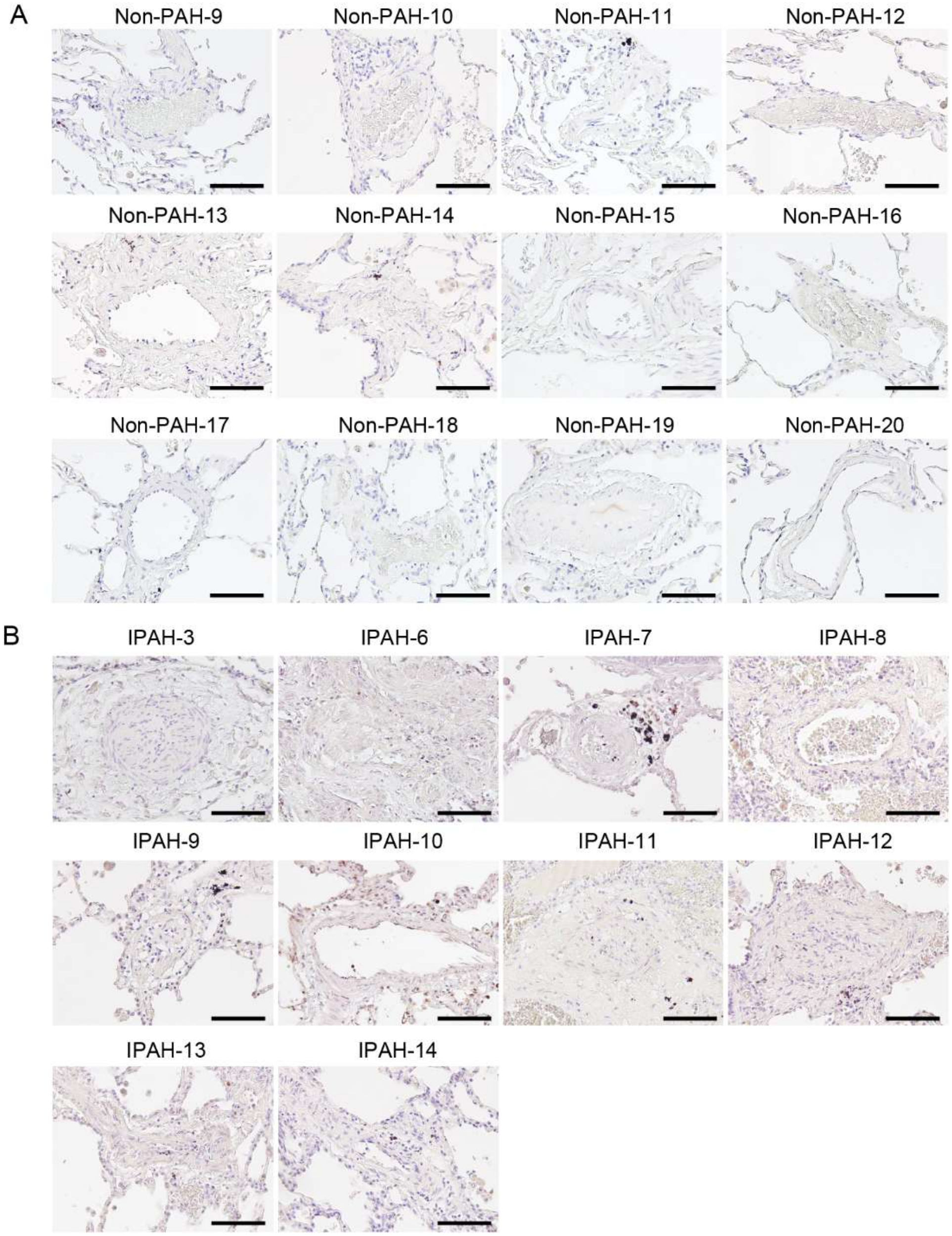

Figure S8. Ki-67 expression in lung tissues.

A, B, Representative immunohistochemical (IHC) images of Ki-67 in lung tissues from non-PAH (**A**; n=12) and IPAH patients (**B**; n=10). Scale bars, 100 μm.

Figure S9

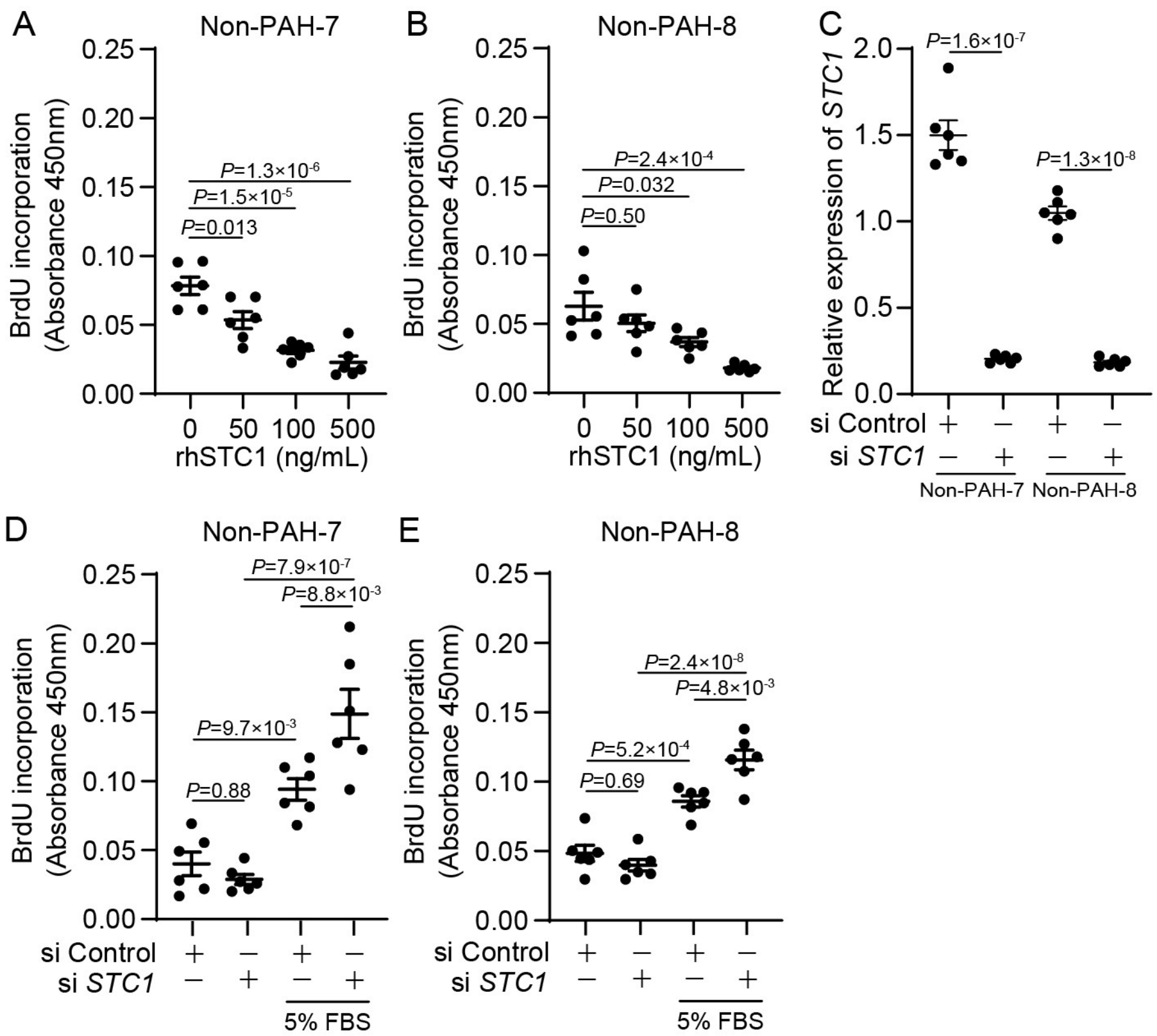

Figure S9. Reproducibility of STC1-mediated regulation of PASMC proliferation in independent non-PAH lots.

A–B, Effect of recombinant human STC1 (rhSTC1) on cell proliferation. Pulmonary artery smooth muscle cells (PASMCs) from independent non-PAH patients (Non-PAH-7 and -8) were analyzed. Cell proliferation was assessed by 5-bromo-2′-deoxyuridine (BrdU) incorporation assay and quantified by ELISA. **C**, Efficiency of *STC1* knockdown. PASMCs from non-PAH patients (Non-PAH-7 and -8) were transfected with small interfering RNA targeting *STC1* (si *STC1*) or control siRNA (si Control), and knockdown

efficiency was assessed by quantitative polymerase chain reaction (qPCR).

D-E, Effect of STC1 knockdown on cell proliferation. Cell proliferation following siRNA-mediated knockdown of STC1 was assessed by BrdU incorporation assay with ELISA in PASMCs from non-PAH patients (Non-PAH-7 and -8). Data are presented as mean ± SEM (n=6). Comparisons among multiple groups were performed using linear mixed-effects models, and post hoc pairwise comparisons were conducted using the *emmeans* with Tukey adjustment. *P* values are indicated.

Figure S10

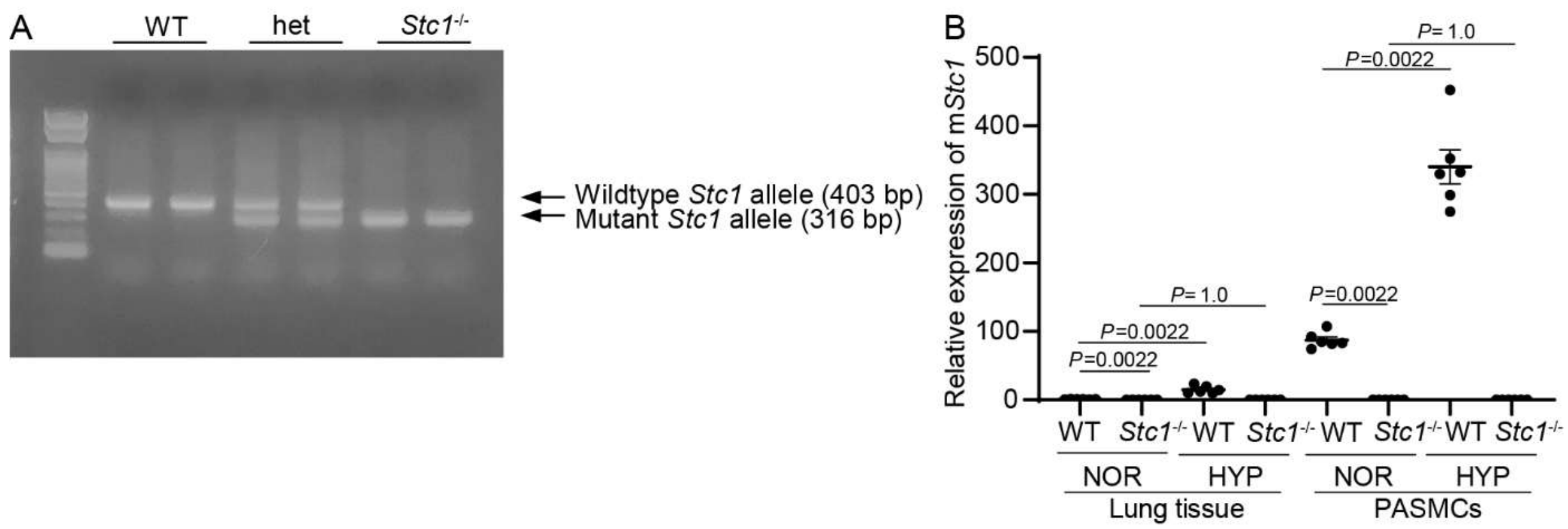

Figure S10. Genotyping of *Stc1*$^{-/-}$ mice and validation of *Stc1* deficiency.

A, Representative agarose gel electrophoresis image of genotyping results. Wild-type and mutant alleles are indicated by arrows. WT, wild-type; het, heterozygous; *Stc1*$^{-/-}$, homozygous knockout. **B**, Expression of *Stc1* mRNA in lung tissues and primary cultured PASMCs from WT and *Stc1*$^{-/-}$ mice following exposure to chronic hypoxia (FiO_2 10% for 4 weeks). Relative expression of *Stc1* mRNA was quantified by quantitative polymerase chain reaction (qPCR). Data are presented as mean ± SEM (n=6 per group). For comparisons among multiple groups, statistical significance was assessed using the Kruskal–Wallis test followed by *post hoc* Mann–Whitney U tests. *P* values are indicated.

Figure S11

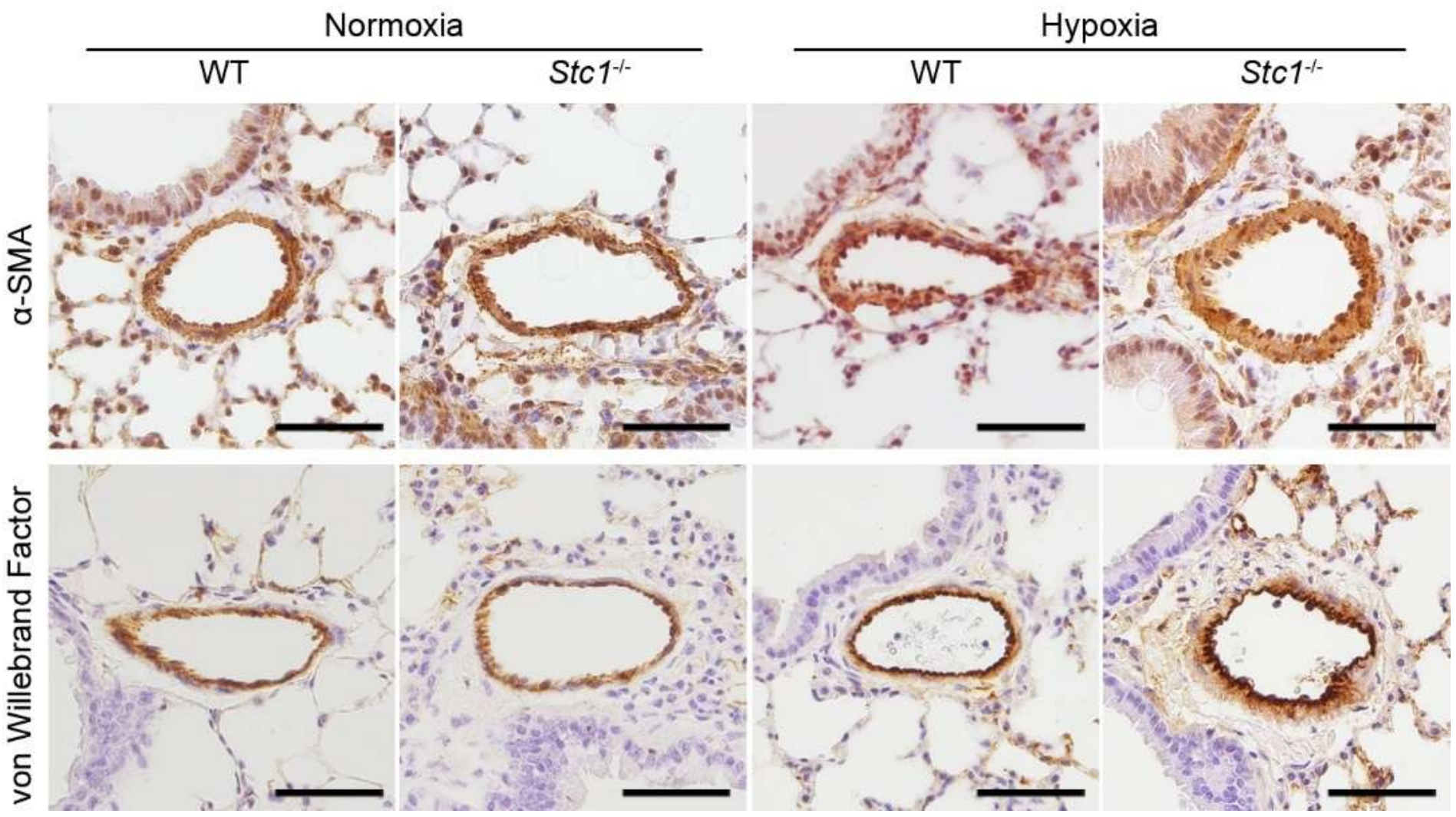

Figure S11. Immunostaining of lung tissue from wild-type (WT) and *Stc1*$^{-/-}$ mice with experimental pulmonary arterial hypertension (PAH).

Representative images of lung sections from WT and *Stc1*$^{-/-}$ mice subjected to the PAH model. The upper panels show staining for α-smooth muscle actin (α-SMA) to visualize smooth muscle cells, and the lower panels show staining for von Willebrand factor to identify endothelial cells. Scale bars: 50 μm.

Figure S12

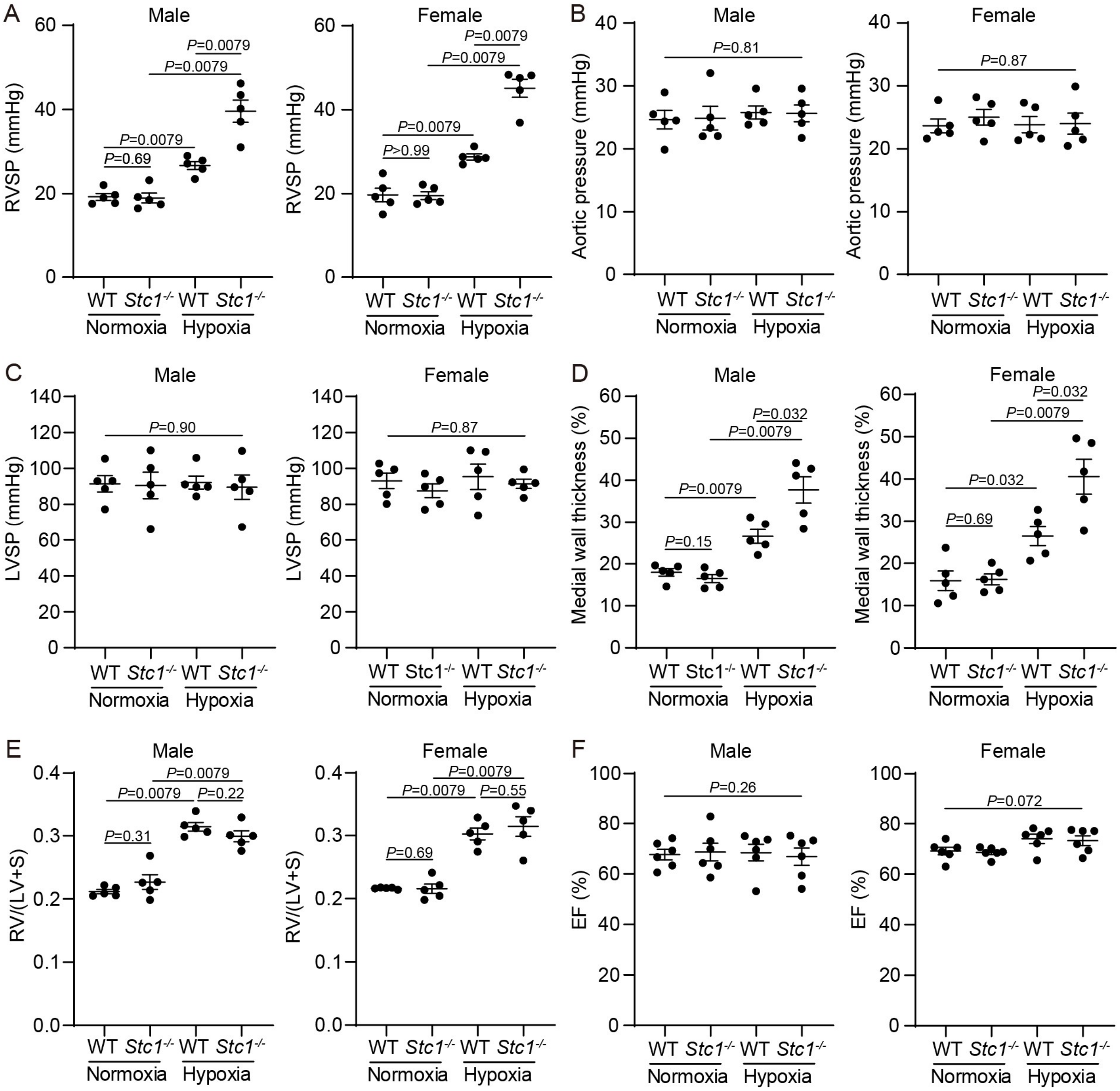

Figure S12. Sex-specific differences in hemodynamic and cardiac functional parameters in wild-type (WT) and *Stc1*$^{-/-}$ mice with experimental pulmonary arterial hypertension (PAH).

A, Right ventricular systolic pressure (RVSP). **B**, Aortic pressure. **C**, Left ventricular

systolic pressure (LVSP). **D**, Medial wall thickness of pulmonary arteries (external diameter 15–100 μm) assessed by histological analysis. **E**, Fulton index (right ventricle/[left ventricle + septum]; RV/[LV+S]) as a measure of right ventricular hypertrophy. **F**, Left ventricular ejection fraction (EF) assessed by echocardiography. For each panel, data from male mice are shown on the left and data from female mice on the right. Each group included 5 mice. Data are presented as mean ± SEM (n=6). Statistical analysis was performed using Kruskal–Wallis test followed by Mann–Whitney U test. *P* values are indicated.

Figure S13

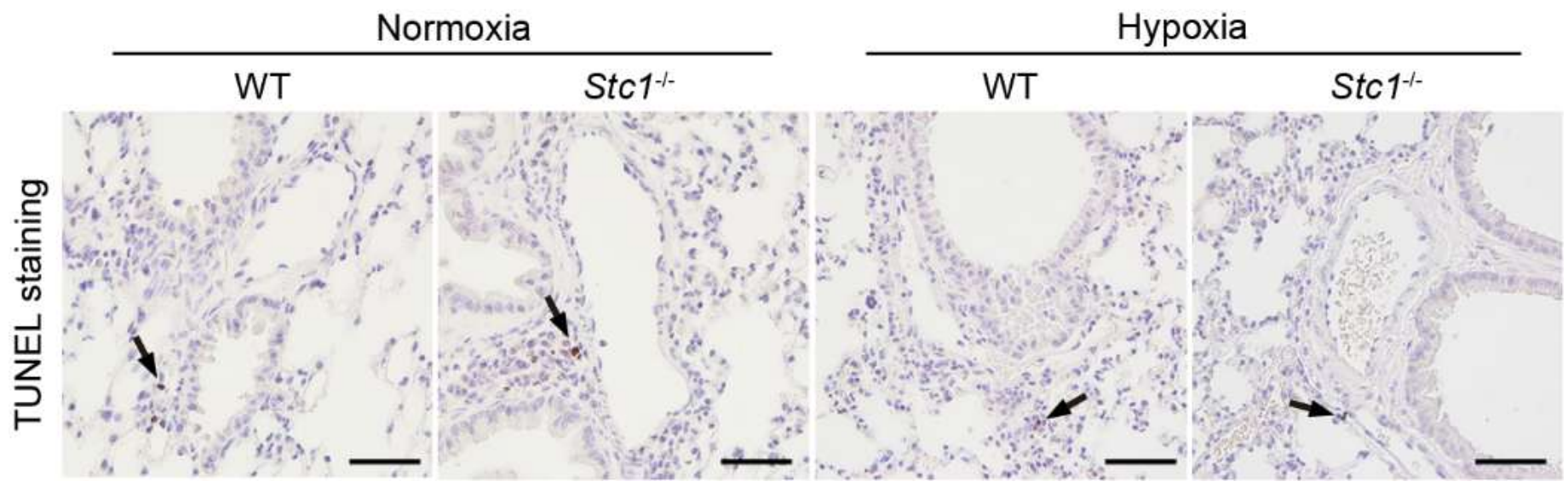

Figure S13. TUNEL staining of lung tissue from wild-type (WT) and *Stc1*$^{-/-}$ mice with experimental pulmonary arterial hypertension (PAH).

Representative images of lung sections from WT and *Stc1*$^{-/-}$ mice subjected to the PAH model and stained for terminal deoxynucleotidyl transferase dUTP nick end labeling (TUNEL) to detect apoptotic cells. Arrows indicate TUNEL-positive cells. Scale bars: 50 μm.

Major Resources Table

Animals (*in vivo* studies)

Mouse strain	Vendor or Source	Background Strain	Sex
Stc1$^{-/-}$	University of Sydney	C57BL/6J	F/M

Human samples

	Age (yr)	Sex	Diagnosis	Specimen Type	Vendor or Source	Passage
Non-PAH-1	47	M	Pulmonary cryptococcosis	Lung tissue	Tokyo Medical University	
Non-PAH-2	67	M	Lung cancer	Primary human Pulmonary arterial smooth muscle cells (PASMCs)	Okayama University	3-10
Non-PAH-3	58	M	Lung cancer	Primary human PASMCs	Okayama University	3-10
Non-PAH-4	30	F	Lymphangioleiomyomatosis	Primary human PASMCs	Okayama University	3-10
Non-PAH-5	54	M	Lung cancer	Primary human PASMCs	Okayama University	3-10
Non-PAH-6	56	F	Mast cell leukemia	Primary human PASMCs	Lonza (CC-2581, #21TL347546)	3-10
Non-PAH-7	61	M	Skin cancer (removed)	Primary human PASMCs	Lonza (CC-2581, #23T223980)	3-10

Non-PAH-8	64	M	Trauma	Primary human PASMCs	Lonza (CC-2581, #24TL007152)	3-10
Non-PAH-9	35	M	Pulmonary fibrosis	Lung tissue	Tokyo Medical University	
Non-PAH-10	20	M	Pulmonary sequestration	Lung tissue	Tokyo Medical University	
Non-PAH-11	32	M	Necrotizing epithelioid granuloma	Lung tissue	Tokyo Medical University	
Non-PAH-12	43	M	Pulmonary mycosis	Lung tissue	Tokyo Medical University	
Non-PAH-13	40	M	Epithelioid granuloma	Lung tissue	Tokyo Medical University	
Non-PAH-14	39	F	Pulmonary hamartoma	Lung tissue	Tokyo Medical University	
Non-PAH-15	34	F	Pulmonary sequestration	Lung tissue	Tokyo Medical University	
Non-PAH-16	34	F	Pulmonary fibrosis	Lung tissue	Tokyo Medical University	
Non-PAH-17	47	F	Necrotizing epithelioid granuloma	Lung tissue	Tokyo Medical University	
Non-PAH-18	26	F	Pulmonary sequestration	Lung tissue	Tokyo Medical University	
Non-	46	F	Necrotizing epithelioid	Lung	Tokyo Medical	

PAH-19			granuloma	tissue	University	
Non-PAH-20	48	F	Necrotizing epithelioid granuloma	Lung tissue	Tokyo Medical University	
IPAH-1	8	F	IPAH	Primary human PASMCs	Okayama University	3-10
IPAH-2	11	M	IPAH	Primary human PASMCs and lung tissue	Okayama University	3-10
IPAH-3	10	M	IPAH	Primary human PASMCs and lung tissue	Okayama University	3-10
IPAH-4	37	F	IPAH	Primary human PASMCs	Okayama University	3-10
IPAH-5	12	M	IPAH	Primary human PASMCs	Okayama University	3-10
IPAH-6	25	M	IPAH	Lung tissue	Okayama University	
IPAH-7	23	F	IPAH	Lung tissue	Okayama University	
IPAH-8	8	F	IPAH	Lung tissue	Okayama University	
IPAH-9	29	M	IPAH	Lung tissue	Okayama University	
IPAH-10	18	M	IPAH	Lung tissue	Okayama University	

IPAH-11	14	M	IPAH	Lung tissue	Okayama University	
IPAH-12	37	F	IPAH	Lung tissue	Okayama University	
IPAH-13	27	F	IPAH	Lung tissue	Okayama University	
IPAH-14	32	F	IPAH	Lung tissue	Okayama University	

Antibodies

Antibody	Vendor or Source	Catalog #	Working Concentration	Lot #	Persistent ID / URL
STC1	Abcam	ab229477	1:1000 (WB) 1:500 (IHC-P)	GR3292203-5	https://www.abcam.com /en-us/products/Primary-antibodies/ stanniocalcin-1-stc-antibody-ab229477
p38	Cell Signaling Technology	#8690	1:1000 (WB)	9	RRID: AB_10999090
phospho-p38	Cell Signaling Technology	#4511	1:1000 (WB)	13	RRID: AB_2139682
p53	Cell Signaling Technology	#2524	1:300 (WB)	22	RRID: AB_331743
phospho-p53	Cell Signaling Technology	#9284	1:500 (WB)	17	RRID: AB_331464
p21	Cell Signaling Technology	#2947	1:1000 (WB)	14	RRID: AB_823586
p27	Cell Signaling Technology	#3686	1:1000 (WB)	5	RRID: AB_2077850
CDK2	Cell Signaling Technology	#2546	1:1000 (WB)	10	RRID: AB_2276129
α-Smooth muscle actin	Sigma	A2547	1:500 (IHC-P)	125M4797V	RRID: AB_476701
von Willebrand factor	DAKO	A0082	1:1000 (IHC-P)	111(101)	RRID: AB_2315602
CD68	Bio-Rad	MCA1957	1:1000 (IHC-P)	1708	RRID: AB_322219
CD86	R and D Systems	AF-141-NA	1:1000 (IHC-P)	AAF0324051	https://www.rndsystems.com/ products/human-b7-2-cd86-antibody_af-141-na
CD206	Abcam	ab64693	1:20000 (IHC-P)	GR3408725-2	RRID: AB_1523910
PIEZO1	ProteinTech	15939-1-AP	1:200 (IHC-P)	147827	RRID: AB_2231460

HIF-1α	Novus Biologicals	NB100-105	1:100 (ICC)	D142032-4	RRID: AB_350048
Ki-67	Abcam	ab16667	1:500 (IHC-P)	1129957-8	RRID: AB_302459
Alexa Fluor 488 anti-Rabbit IgG	Invitrogen	A11034	1:250 (IF)	1423009	RRID: AB_143165
Biotinylated anti-Rabbit IgG	Vector Laboratories	BA-1000	1:400 (IHC-P)	WOVUS34995	RRID: AB_2313606
Biotinylated anti-Rat IgG	Vector Laboratories	BA-4000	1:400 (IHC-P)	ZF1002	RRID: AB_2336200
Biotinylated anti-Goat IgG	Vector Laboratories	BA-5000	1:400 (IHC-P)	ZF0307	RRID: AB_2336126
HRP-linked Anti-Mouse IgG	Cell Signaling Technology	7076	1:500 (WB)	38	RRID: AB_330924
HRP-linked Anti-Rabbit IgG	Cell Signaling Technology	7074	1:500 (WB)	33	RRID: AB_2099233

siRNA sequences

	Vendor or Source	Accession Number	Sequence (5′-3′)
Control-1 - Forward	Horizon Discovery	N/A	UGGUUUACAUGUCGACUAAUU
Control-1 - Reverse	Horizon Discovery	N/A	UUAGUCGACAUGUAAACCAUU
Control-2 - Forward	Horizon Discovery	N/A	UGGUUUACAUGUUGUGUGAUU
Control-2 - Reverse	Horizon Discovery	N/A	UCACACAACAUGUAAACCAUU
Control-3 - Forward	Horizon Discovery	N/A	UGGUUUACAUGUUUUCUGAUU
Control-3 - Reverse	Horizon Discovery	N/A	UCAGAAAACAUGUAAACCAUU
Control-4 - Forward	Horizon Discovery	N/A	UGGUUUACAUGUUUUCCUAUU
Control-4 - Reverse	Horizon Discovery	N/A	UAGGAAAACAUGUAAACCAUU
STC1-1 - Forward	Horizon Discovery	NM_003155.3	AAACGCACAUCCCAUGAGAUU
STC1-1 - Reverse	Horizon Discovery	NM_003155.3	UCUCAUGGGAUGUGCGUUUUU
STC1-2 - Forward	Horizon Discovery	NM_003155.3	GGGAAAAGCAUUCGUCAAAUU
STC1-2 - Reverse	Horizon Discovery	NM_003155.3	UUUGACGAAUGCUUUUCCCUU
STC1-3 - Forward	Horizon Discovery	NM_003155.3	GUACAGCGCUGCUAAAUUUUU
STC1-3 - Reverse	Horizon Discovery	NM_003155.3	AAAUUUAGCAGCGCUGUACUU
STC1-4 - Forward	Horizon Discovery	NM_003155.3	CAACAGAUACUAUAACAGAUU
STC1-4 - Reverse	Horizon Discovery	NM_003155.3	UCUGUUAUAGUAUCUGUUGUU
PIEZO1-1 - Forward	Horizon Discovery	NM_001142864.4	GCAGCAUGACAGACGACAUUU
PIEZO1-1 - Reverse	Horizon Discovery	NM_001142864.4	AUGUCGUCUGUCAUGCUGCUU
PIEZO1-2 - Forward	Horizon Discovery	NM_001142864.4	UGGAGUAUGCCAACGAGAAUU
PIEZO1-2 - Reverse	Horizon Discovery	NM_001142864.4	UUCUCGUUGGCAUACUCCAUU
PIEZO1-3 - Forward	Horizon Discovery	NM_001142864.4	UGGCUGAUGUUGUCGACUUUU
PIEZO1-3 - Reverse	Horizon Discovery	NM_001142864.4	AAGUCGACAACAUCAGCCAUU
PIEZO1-4 - Forward	Horizon Discovery	NM_001142864.4	GCGCAUCAGUCUACGUUUUUU
PIEZO1-4 - Reverse	Horizon Discovery	NM_001142864.4	AAAACGUAGACUGAUGCGCUU

qPCR primer sequences

	Vendor or Source	Accession number	Sequence (5′-3′)	Amplicon size (bp)
STC1 - Forward	Eurofins Genomics	NM_003155.3	AGGTGCAGGAAGAGTGCTACA	80

STC1 - Reverse	Eurofins Genomics	NM_003155.3	GACGACCTCAGTGATGGCTT	
PIEZO1 - Forward	Eurofins Genomics	NM_001142864.4	GCCCAACTACTGCCTCTTCC	263
PIEZO1 - Reverse	Eurofins Genomics	NM_001142864.4	GCTGCCACTCCTCTGTGC	
mStc1 - Forward	Eurofins Genomics	NM_009285.3	CTTGCCATTCGGAGGTGTTC	175
mStc1 - Reverse	Eurofins Genomics	NM_009285.3	GGCTTCGGACAAGTCTGTTG	
VEGFA primer site – Forward	Eurofins Genomics	-	AGACTCCACAGTGCATACGTG	245
VEGFA primer site – Reverse	Eurofins Genomics	-	AGTGTGTCCCTCTGACAATG	
STC1 primer site – Forward	Eurofins Genomics	-	GATAGTAAGTGAGCAAAATAAGAA	109
STC1 primer site - Reverse	Eurofins Genomics	-	CTCCGTTGCTCTCACCAGAA	

ARRIVE GUIDELINES

The ARRIVE guidelines (https://arriveguidelines.org/) are a checklist of recommendations to improve the reporting of research involving animals. Key elements of the study design should be included below to better enable readers to scrutinize the research adequately, evaluate its methodological rigor, and reproduce the methods or findings.

Study Design

**Groups**	**Sex**	**Age**	**Number (prior to experiment)**	**Number (after termination)**	**Littermates (Yes/No)**
WT-normoxia	F/M	3 months	10 (F; n=5, M; n=5)	10 (F; n=5, M; n=5)	Yes
WT-hypoxia	F/M	3 months	10 (F; n=5, M; n=5)	10 (F; n=5, M; n=5)	Yes
Stc1$^{-/-}$-normoxia	F/M	3 months	10 (F; n=5, M; n=5)	10 (F; n=5, M; n=5)	Yes
Stc1$^{-/-}$-hypoxia	F/M	3 months	10 (F; n=5, M; n=5)	10 (F; n=5, M; n=5)	Yes
WT-hypoxia-vehicle	F/M	3 months	6 (F; n=4, M; n=2)	6 (F; n=4, M; n=2)	Yes
WT-Hypoxia-rhSTC1	F/M	3 months	6 (F; n=4, M; n=2)	6 (F; n=4, M; n=2)	Yes
Stc1$^{-/-}$-hypoxia-vehicle	F/M	3 months	6 (F; n=5, M; n=1)	6 (F; n=5, M; n=1)	Yes
Stc1$^{-/-}$-hypoxia-rhSTC1	F/M	3 months	6 (F; n=4, M; n=2)	6 (F; n=4, M; n=2)	Yes

Sample Size

Six to ten mice were used per group (total 64 mice). Sample sizes were determined based on previous hypoxia-induced pulmonary hypertension studies that showed sufficient power to detect changes in right ventricular systolic pressure (RVSP) and medial wall thickness. No formal *a priori* power calculation was performed.

Inclusion Criteria

Mice of confirmed genotype (WT or *Stc1*$^{-/-}$), aged 3 months, maintained under specific-pathogen-free conditions, were included in the study.

Exclusion Criteria

Animals were excluded only in cases of technical failure during right-heart catheterization. No mice

were excluded after randomization.

Randomization

Randomization was not applied because experimental groups were predetermined by genotype (WT or *Stc1*$^{-/-}$) and exposure condition (normoxia or hypoxia). To minimize bias, the order of hemodynamic and histological analyses was alternated among groups.

Blinding

Investigators performing hemodynamic and histological analyses were blinded to group allocation

during data analysis, although blinding was not feasible during the experimental procedures.
