# Supplementary material for: Mechanosensitive Stanniocalcin-1 Suppresses Smooth Muscle Cell Proliferation and Attenuates Experimental Pulmonary Hypertension
Source: Circ Res. 2026 Sep 9;139(9):e327869. doi: 10.1161/CIRCRESAHA.125.327869 (PMC13649279; doi:10.1161/CIRCRESAHA.125.327869)
Supplement: Supplementary file 2 [file res-139-e327869-s002.pdf]

Uncropped image for Figures 2C

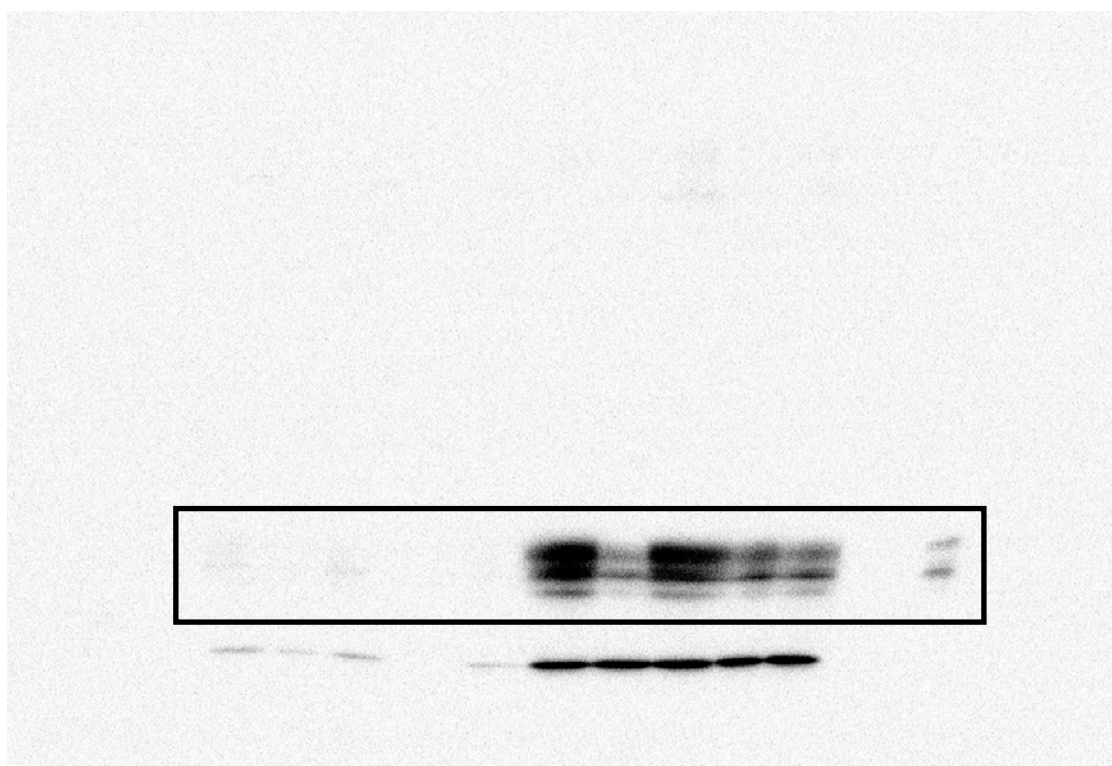

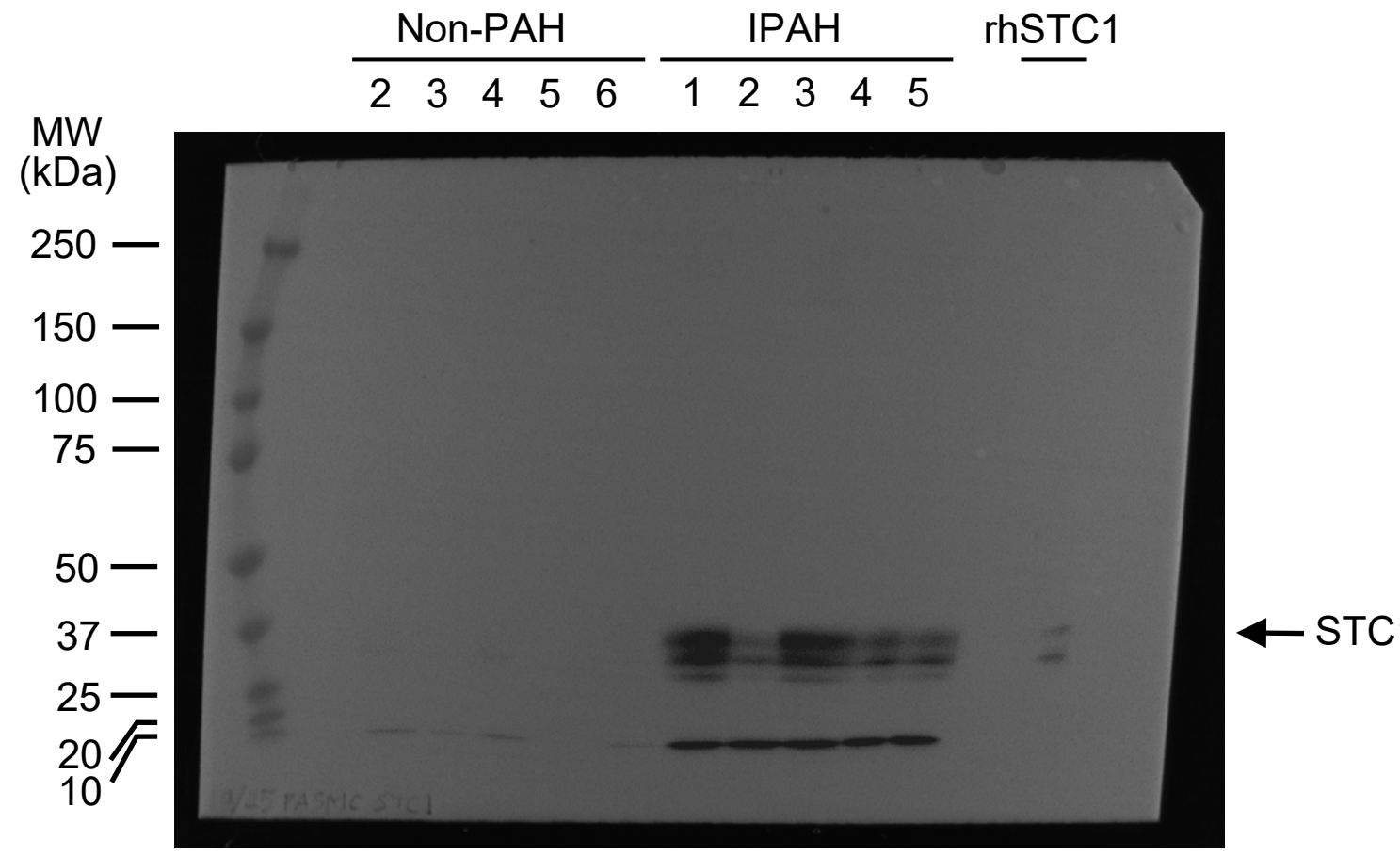

Left: Uncropped chemiluminescence image of STC1 (signal only). The cropped region displayed in the main figure is indicated by a box. Right: Merged chemiluminescence signal and visible-light marker image of the same blot.

Uncropped image for Figures 5A

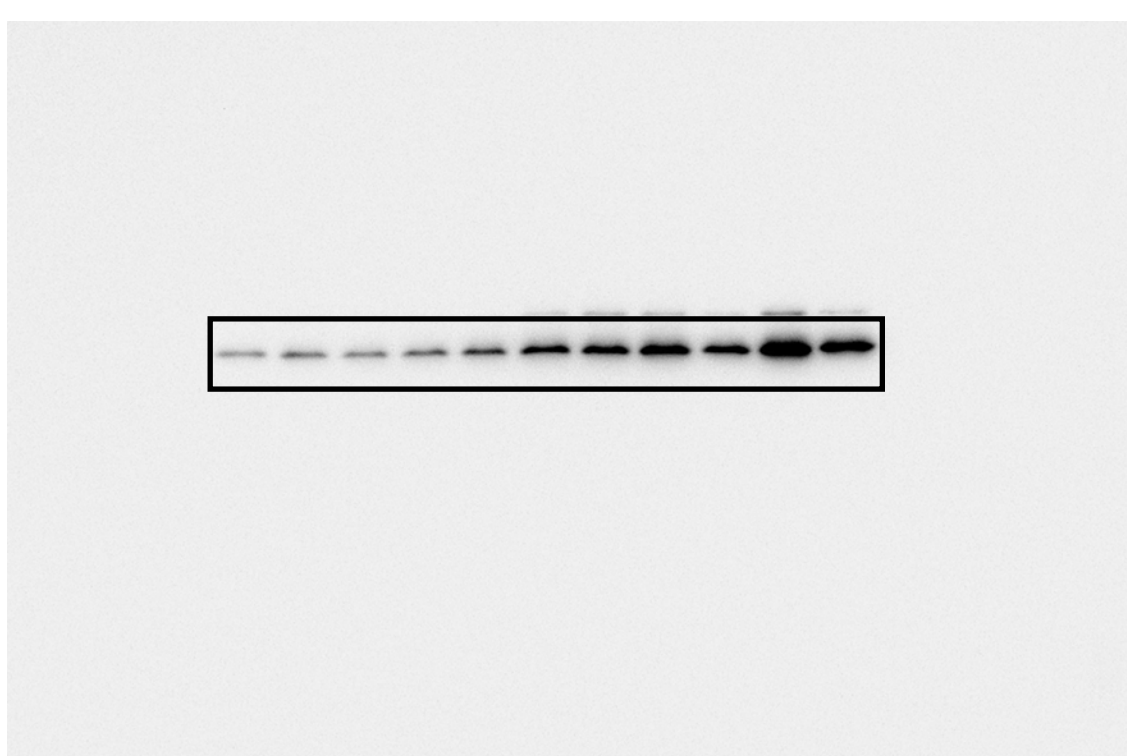

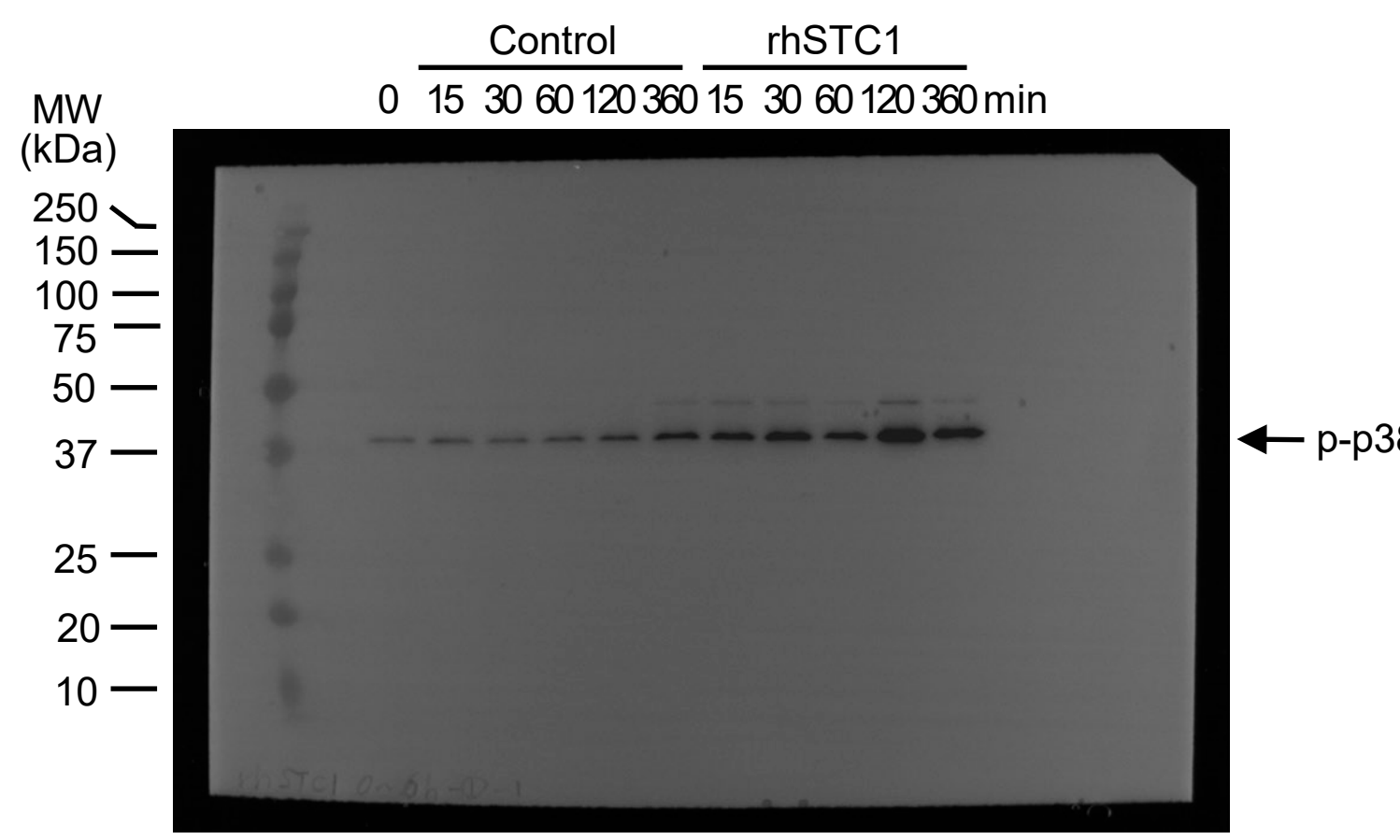

Left: Uncropped chemiluminescence image of phospho-p38 (p-p38, signal only). The cropped region displayed in the main figure is indicated by a box. Right: Merged chemiluminescence signal and visible-light marker image of the same blot.

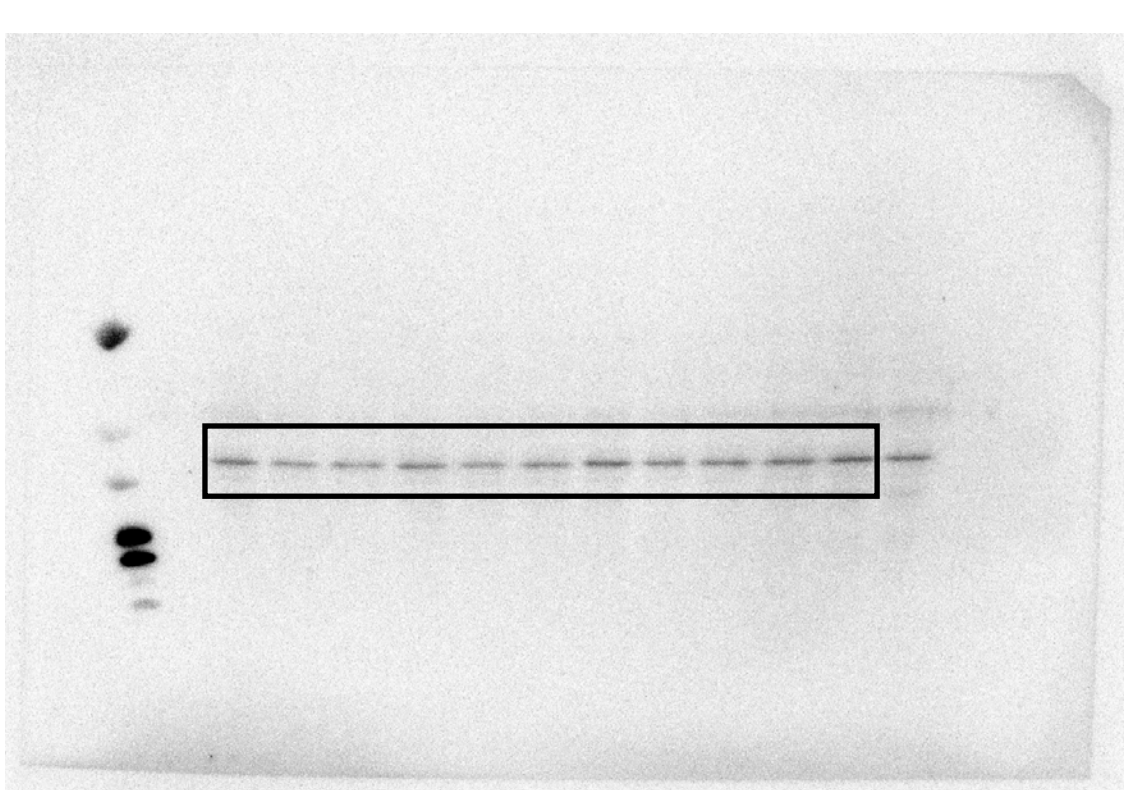

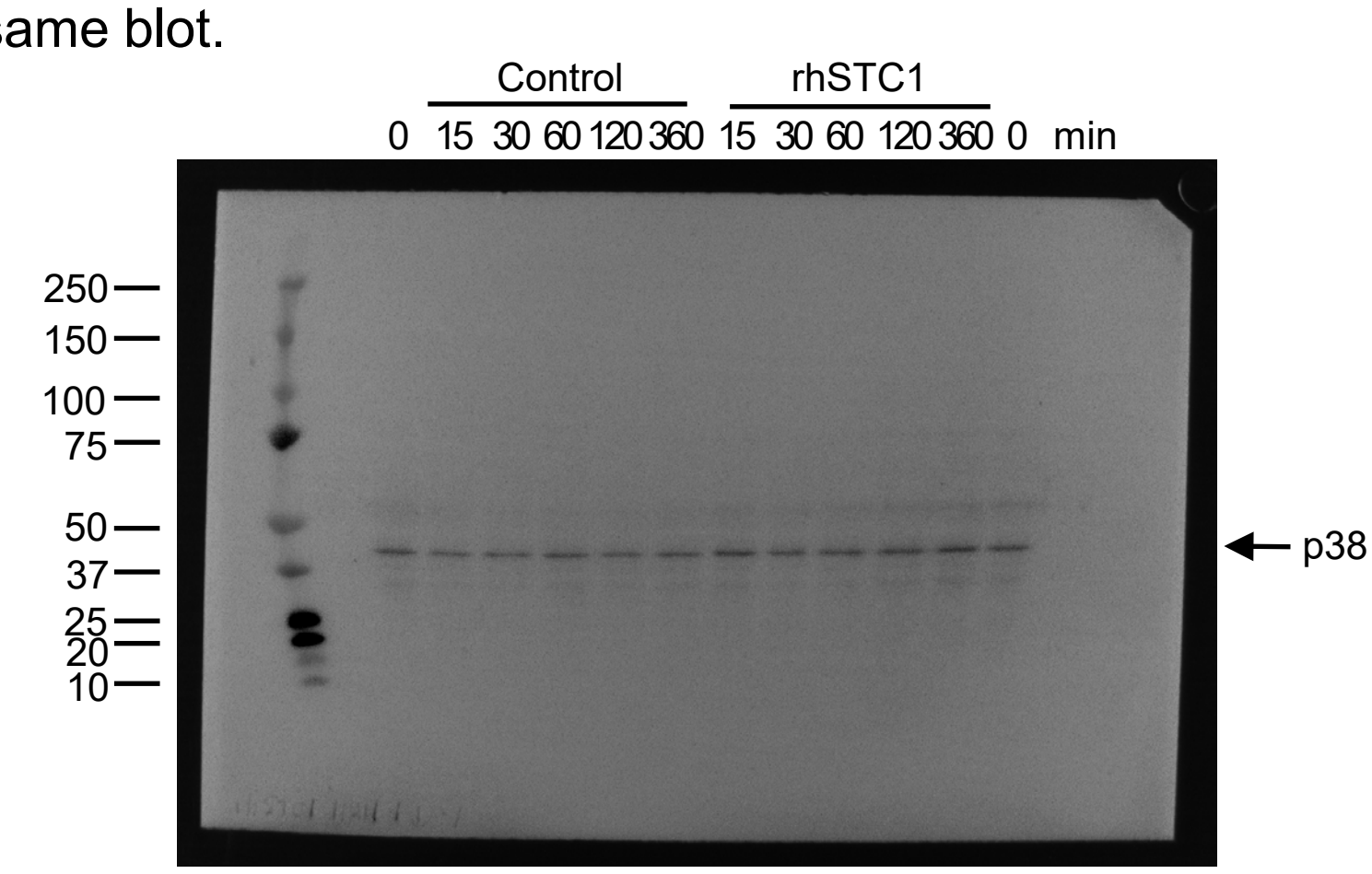

Left: Uncropped chemiluminescence image of p38 (signal only). The cropped region displayed in the main figure is indicated by a box. Right: Merged chemiluminescence signal and visible-light marker image of the same blot.

Uncropped images for Figures 5B

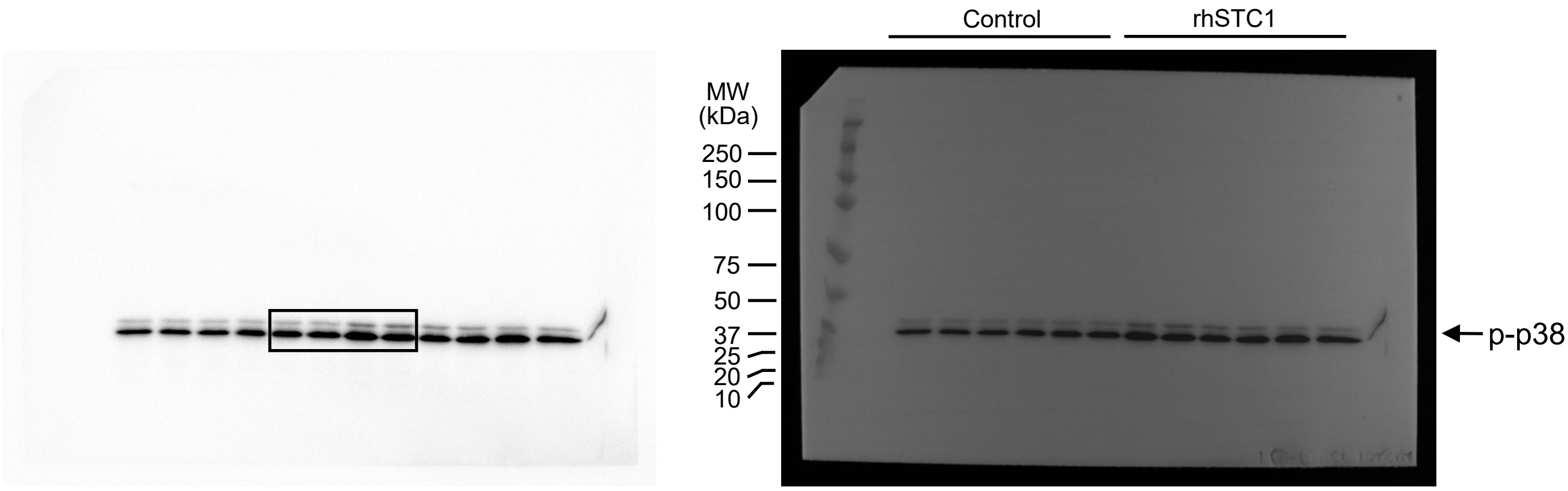

Left: Uncropped chemiluminescence image of p-p38 (signal only). The cropped region displayed in the main figure is indicated by a box. Right: Merged chemiluminescence signal and visible-light marker image of the same blot.

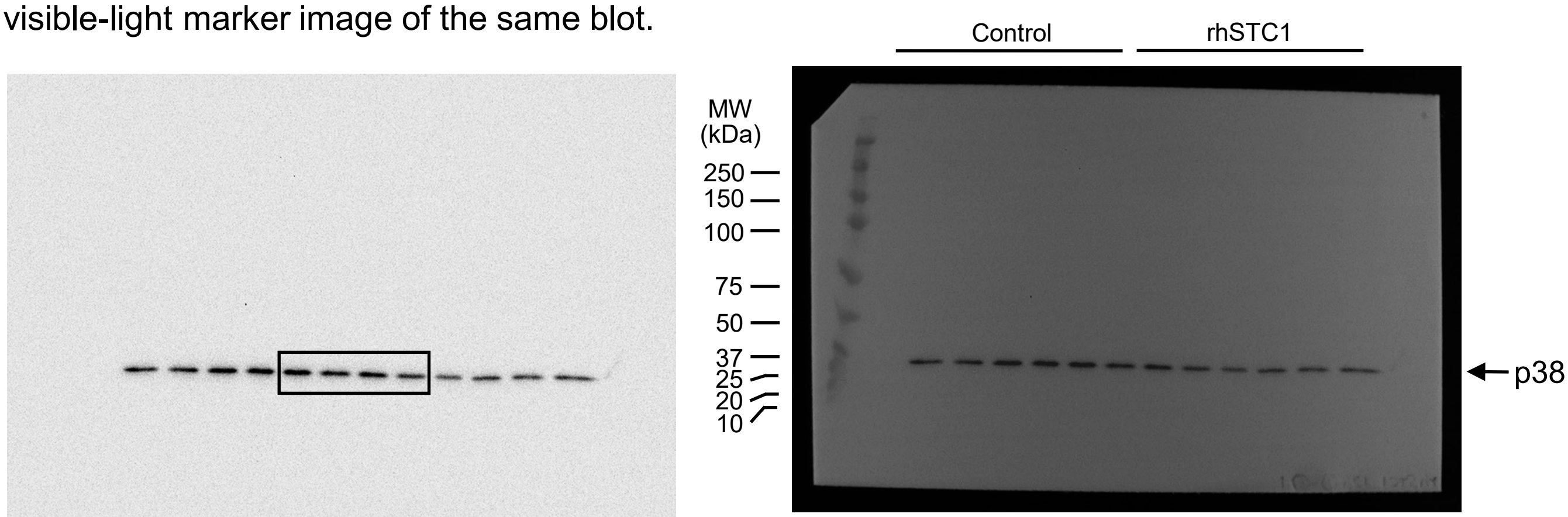

Left: Uncropped chemiluminescence image of p38 (signal only). The cropped region displayed in the main figure is indicated by a box. Right: Merged chemiluminescence signal and visible-light marker image of the same blot.

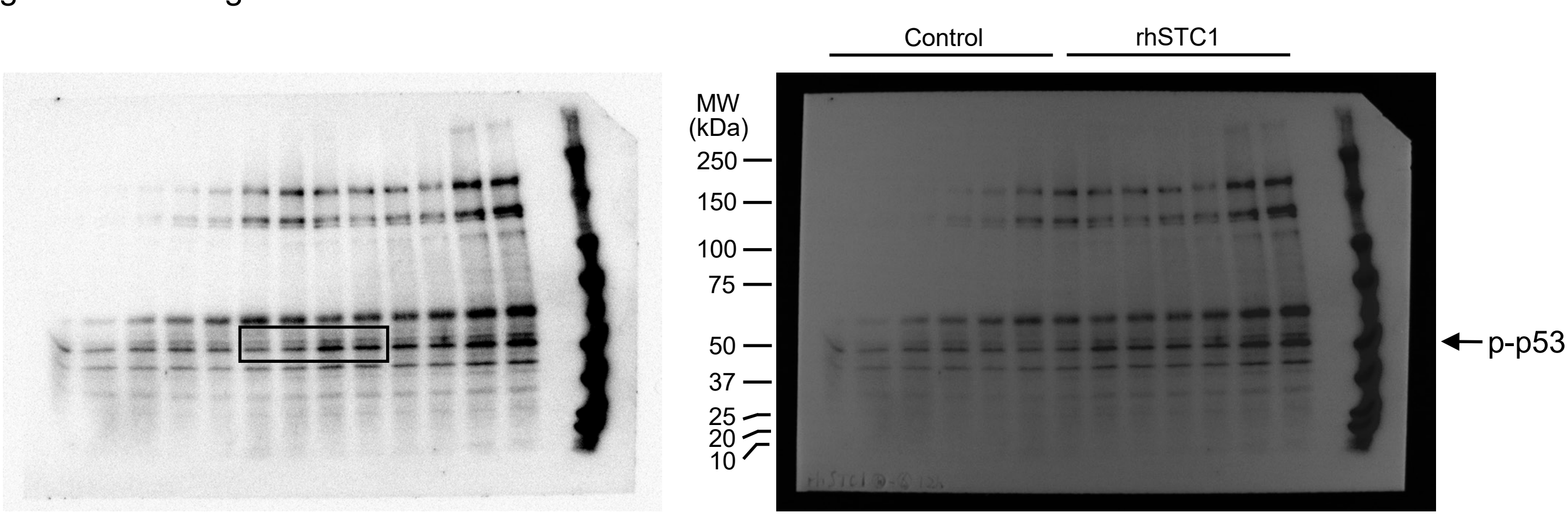

Left: Uncropped chemiluminescence image of phospho-p53 (p-p53, signal only). The cropped region displayed in the main figure is indicated by a box. Right: Merged chemiluminescence signal and visible-light marker image of the same blot.

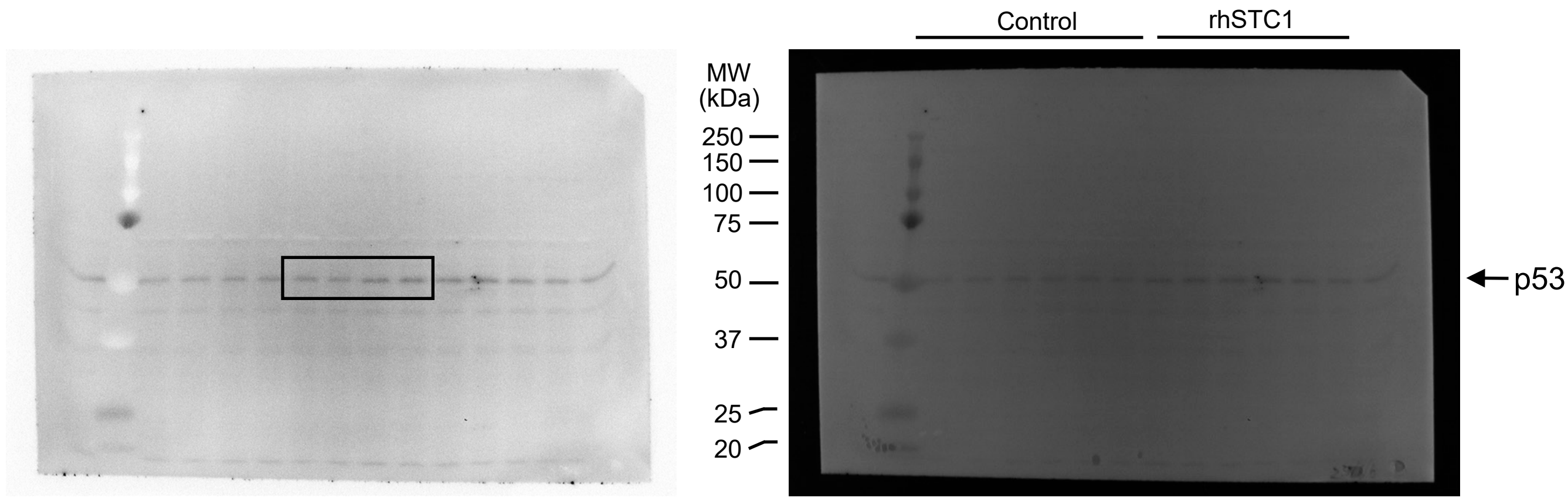

Left: Uncropped chemiluminescence image of p53 (signal only). The cropped region displayed in the main figure is indicated by a box. Right: Merged chemiluminescence signal and visible-light marker image of the same blot.

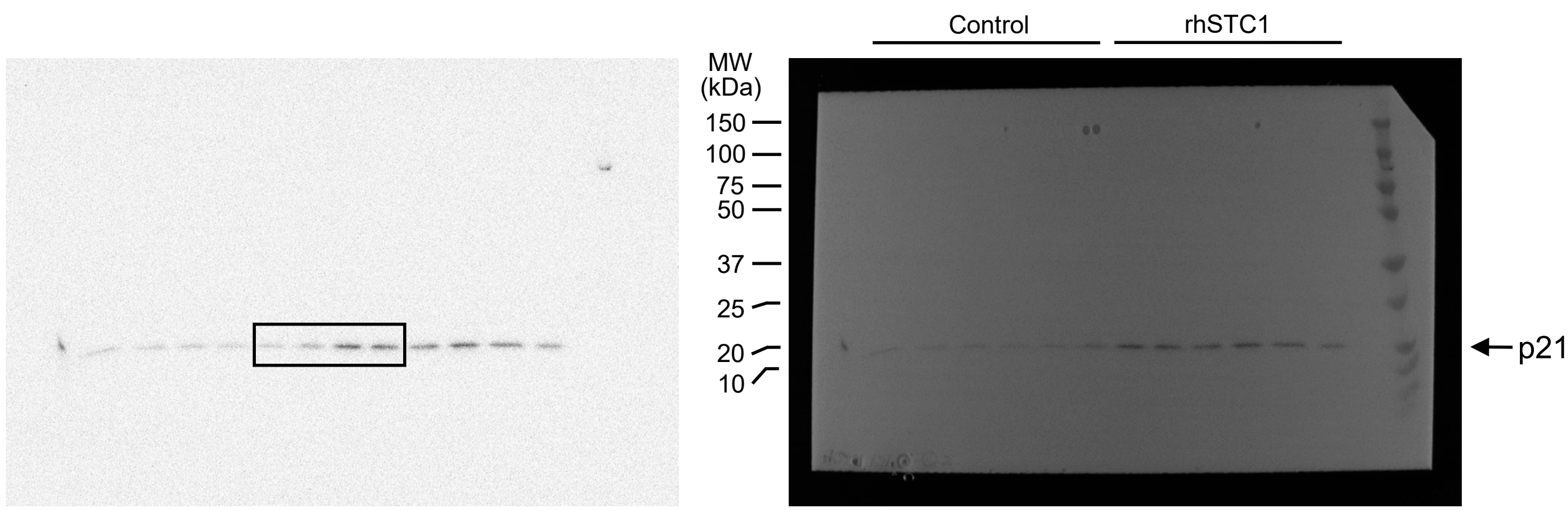

Left: Uncropped chemiluminescence image of p21 (signal only). The cropped region displayed in the main figure is indicated by a box. Right: Merged chemiluminescence signal and visible-light marker image of the same blot.

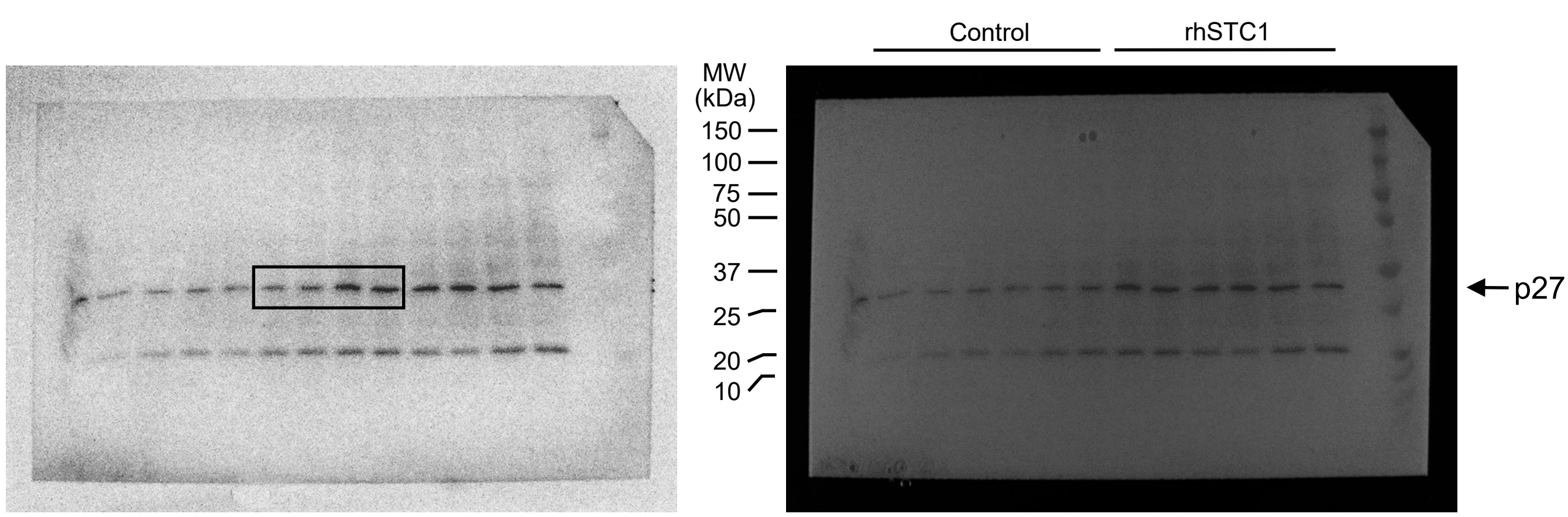

Left: Uncropped chemiluminescence image of p27 (signal only). The cropped region displayed in the main figure is indicated by a box. Right: Merged chemiluminescence signal and visible-light marker image of the same blot.

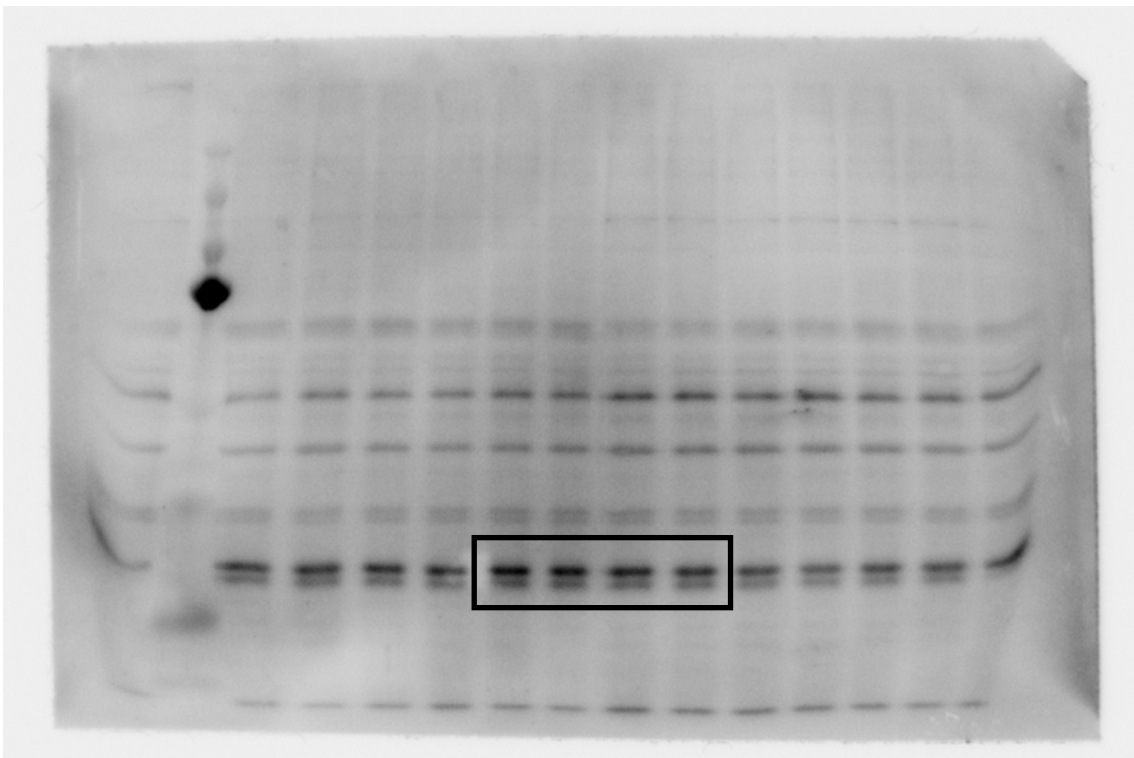

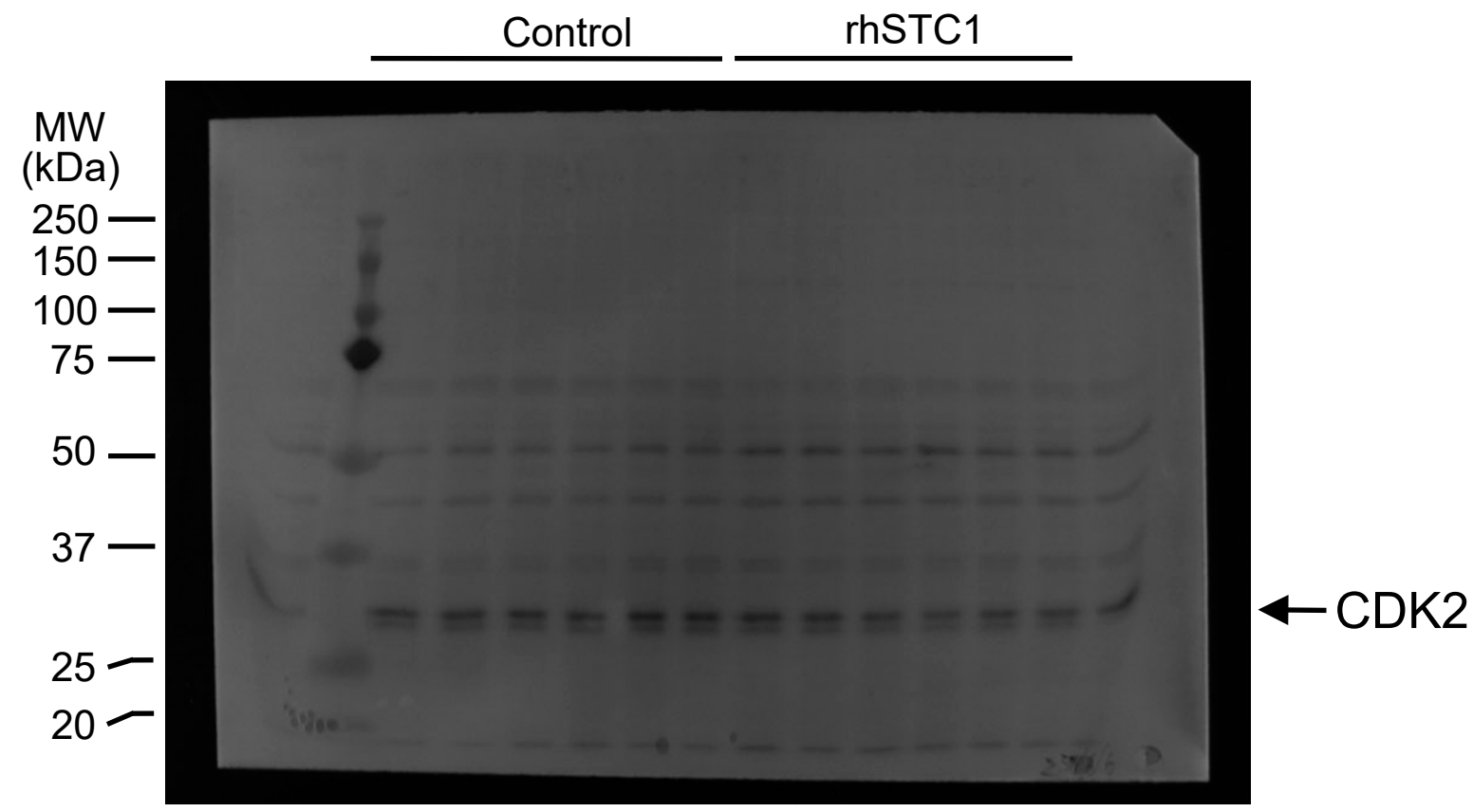

Left: Uncropped chemiluminescence image of CDK2 (signal only). The cropped region displayed in the main figure is indicated by a box. Right: Merged chemiluminescence signal and visible-light marker image of the same blot.

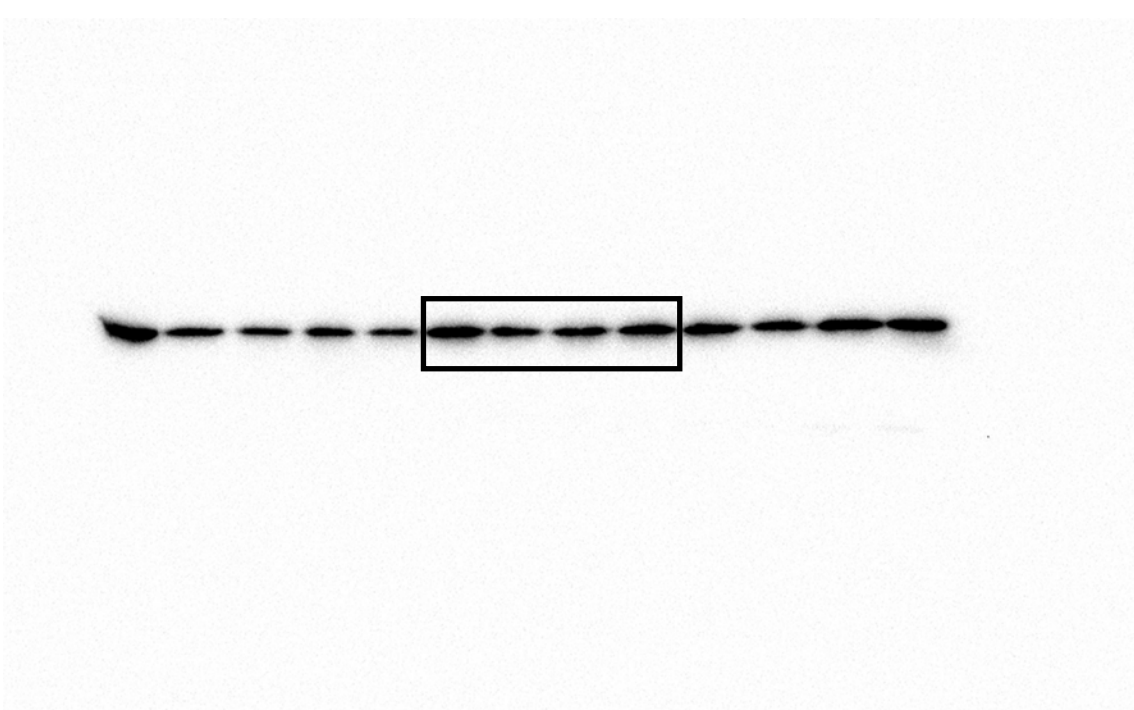

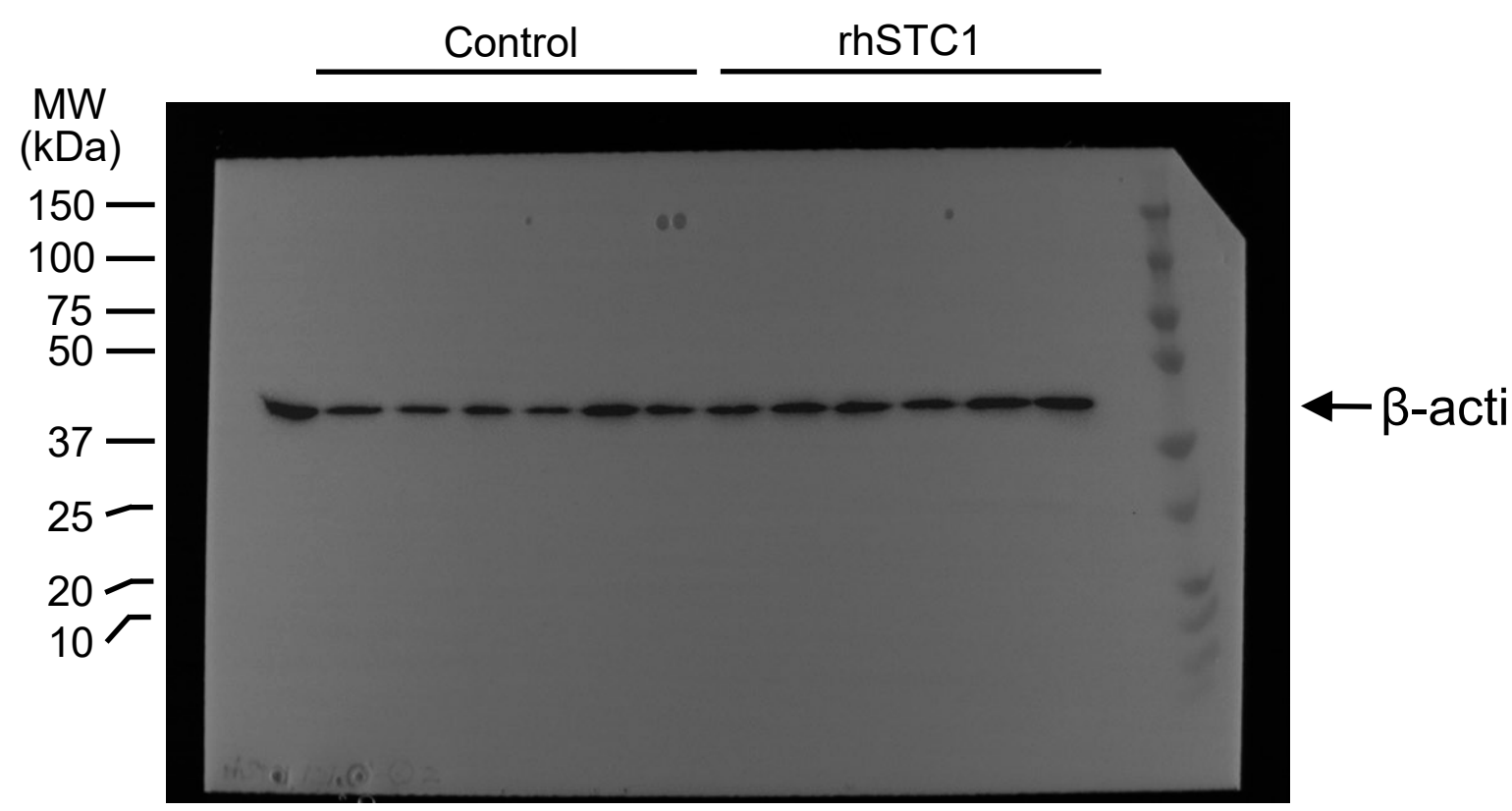

Left: Uncropped chemiluminescence image of β-actin (signal only). The cropped region displayed in the main figure is indicated by a box. Right: Merged chemiluminescence signal and visible-light marker image of the same blot.
